# Causally Informative Entropic Inequalities within Families of Distributions with Shared Marginals

**DOI:** 10.3390/e28040472

**Published:** 2026-04-20

**Authors:** Daniel Chicharro

**Affiliations:** Department of Computer Science, City St George’s, University of London, Northampton Square, London EC1V 0HB, UK; chicharro31@yahoo.es

**Keywords:** causality, directed acyclic graphs, causal discovery, structure learning, marginal scenarios, hidden variables, mutual information, entropic inequalities, data processing inequalities, maximum entropy, minimum information, instrumental inequality, shannon entropy cone, information causality, 62H22, 62D20, 94A15, 94A17

## Abstract

The joint probability distribution of observable variables from a system is constrained by the underlying causal structure. In the presence of hidden variables, untestable independencies that involve hidden variables lead to testable causally-imposed inequality constraints for observable variables, whose violation can reject the compatibility of a causal structure with data. One type of causally informative inequalities is entropic inequalities, which appear in the space of entropic terms associated with the distribution of observable variables. We derive a new type of minimum information (minInf) entropic inequalities that substantially increases causal inference power. These new entropic inequalities appear when considering the constraints that the causal structure imposes on entropic terms determined by information minimization within families of distributions that preserve sets of marginals shared with the original distribution. We introduce a new family of minInf data processing inequalities and a procedure to recursively combine different types of data processing inequalities to create tighter testable entropic inequalities. We extensively illustrate the applicability of this procedure in the instrumental causal scenario, integrating the new inequalities with standard instrumental entropic inequalities constructed with multivariate instrumental sets. We also provide additional examples with other types of entropic inequalities, such as the Information Causality and Groups-Decomposition inequalities.

## 1. Introduction

Understanding which causal structures are compatible with a set of observational data is a common question in science. The underlying causal structure of a system creates constraints on the probability distribution of variables generated from it [1,2,3], which helps to reversely infer which causal structures are compatible with the data. Causal learning algorithms based on conditional independencies [1,2,4] reconstruct a partially oriented graph [5] that represents the equivalence class of all causal structures compatible with the set of conditional independencies present in the distribution of the observable variables (the so-called Markov equivalence class). However, in most real-world scenarios, the components of a system are only partially observed, and the presence of hidden variables creates dependencies among the observable variables that limit the degree to which Markov equivalence classes narrow down the set of causal structures compatible with the data.

Beyond statistical independencies in the joint distribution, the underlying causal structure can also be reflected in other equality constraints imposed to the observable variables. These constraints comprise functional equality constraints [6,7] and independencies that originate from further assumptions about the functional form of the generative mechanisms of the variables [3,8,9,10,11,12,13]. Additionally, nonverifiable conditional independencies that involve hidden variables can manifest themselves through inequality constraints that only involve observable variables [14,15,16]. Unlike equality constraints, inequality constraints provide necessary but not sufficient conditions for the compatibility of data with a certain causal structure. Data violations of inequalities enforced by a causal structure allow discarding that causal structure as the one generating the data. Accordingly, causal inference power is increased when deriving tighter inequalities. Causally informative inequalities comprise inequalities derived in the probability space, such as Bell-type inequalities [17,18], instrumental inequalities [19,20], and interventional inequalities [21], as well as entropic inequalities derived in the space of the entropic terms associated with the observable variables [14,22].

Causally informative entropic inequalities can be derived with two alternative approaches. One approach is to derive a specific entropic inequality departing from a concrete equality that involves hidden variables and then using the conditional independencies associated with the causal structure to derive for the two sides of the equality upper and lower bounds which do not contain hidden variables [23,24,25,26]. In order to derive a testable inequality that only contains observable variables, this approach relies on the data processing (DP) inequality [27] to replace hidden variables by less informative observable variables. In a second approach, all the testable causally informative inequalities imposed by a causal structure are derived reducing the set of equalities and inequalities that characterize the whole system –comprising observable and hidden variables– to the marginal scenario associated with the observable variables [14,24]. This approach combines the inequalities that define the Shannon entropic cone [28], i.e., associated with the nonnegativity, monotonicity, and submodularity properties of entropy, and all additional independence constraints related to all the variables in the causal structure. Subsequently, variable elimination is performed to extract the resulting constraints that only involve observable variables. While this marginalization problem is algorithmically solvable [14], its implementation for large systems is challenging and furthermore does not provide an explicit constructive recipe that allows tracing the resulting inequalities in terms of the existing conditional independencies.

These methods to construct causally informative entropic inequalities traditionally rely on entropic terms associated with the original joint distribution of the observable variables. However, specifically for the so-called Groups-Decomposition inequalities [25,26], it has been shown [26] that new as well as tighter more informative inequalities can be derived if the entropic terms of the original joint distribution are combined with maximum entropy entropic terms. Chicharro and Nguyen [26] introduced a DP inequality for the maximum entropy measure of *unique information *[29], a measure originally proposed to decompose mutual information into redundant, unique, and synergistic components [30]. The maximum entropy unique information is defined by an information minimization within a family of distributions constrained to share some marginals of the original distribution of the observable variables. In this work, we capitalize on an extended combination of the original entropic terms and additional entropic terms defined by information minimization within a broader set of different families. We introduce novel procedures to obtain new and tighter entropic inequalities incorporating these additional entropic terms.

To motivate our derivations, we first proceed with the first approach to derive causally informative entropic inequalities. That is, we focus on concrete causal structures and apply an explicit procedure that involves DP inequalities. Specifically, we focus on instrumental entropic inequalities [24], which appear in the causal scenario of instrumental variables [19]. We derive new instrumental entropic inequalities comprising maximum entropy unique information terms. This allows us to characterize a procedure to recursively combine different DP inequalities to create tighter instrumental entropic inequalities. We then introduce a much wider family of DP inequalities for information terms determined by constrained minimization. These minimum information (minInf) terms are defined within families of distributions that share sets of marginals of the original joint distribution. MinInf DP inequalities are then used to derive tighter instrumental inequalities. Subsequently, we indicate how new entropic inequalities can be derived not only from the sequential application of new minInf DP inequalities, but also as a marginalization problem.

Overall, the minInf DP inequalities that we develop, and the procedure to sequentially combine them, provide a general tool to derive new types of entropic inequalities and to extend existing ones thanks to the incorporation of additional information terms to obtain tighter lower bounds. To illustrate the generality of these tools, we finally also examine how other well-known types of causally informative entropic inequalities [23,25,26] can equally be extended into inequalities with an increased causal inference power. While entropic inequalities have also been formulated for quantum systems [23,31], in this work we restrict our derivation to classical Shannon entropy measures. The Discussion section comments on potential extensions.

This paper is organized as follows. In Section 2, we review existing results relevant for our work. In Section 3.1, we derive instrumental entropic inequalities with unique information terms. In Section 3.2, we compare the new inequalities to standard instrumental inequalities with multivariate instrumental sets, identifying conditions in which the new inequalities provide additional causal inference power. In Section 3.3, we show that causal inference power is increased not only using the DP inequality of unique information instead of the standard DP inequality, but also iteratively combining them. In this way, we identify a procedure to iteratively combine multiple DP inequalities. This procedure is further developed in Section 3.4, where we introduce a general type of DP inequalities for minInf information terms and combine them recursively to add observable minInf information terms as lower bounds of information terms with hidden variables. In Section 3.5, we apply this procedure specifically to build more causally informative instrumental entropic inequalities. Section 3.6 reframes the use of minInf information terms for causal learning with the optics of a marginalization problem. We indicate how to extend the Shannon entropy cone to minInf Shannon entropy cones that jointly characterize minInf families. Marginalization of the hidden variables within this joint space produces also the causally informative entropic inequalities that contain minInf entropic terms. Finally, to provide broader examples of applicability of our methods, in Section 3.7 we show how to extend two other types of causally informative entropic inequalities, namely Groups-Decompositions inequalities [25,26] and the Information Causality inequality [23,31].

## 2. Methods

In this section we review the relation between causal graphs and conditional independencies, the standard data processing inequality, the standard instrumental entropic inequality, as well as the formulation of minimum mutual information quantities, comprising a measure of maximum entropy unique information.

### 2.1. Causal Graphs and Conditional Independencies

We review Directed Acyclic Graphs (DAGs) and the relation between causal structures and dependencies. A DAG $G=(\bar{V};E)$ associated with a set of random variables $\bar{V}=\left\{{\bar{V}}_{1},\ldots ,{\bar{V}}_{n}\right\}$ consists of nodes $\bar{V}$ and edges E between the nodes, where $\bar{V}$ refers both to a variable and its corresponding node. Note that in general $\bar{V}$ can comprise both observable and hidden variables; we will later use specifically letter *U* for hidden variables. The set of edges E contains $({\bar{V}}_{i};{\bar{V}}_{j})\in E$ for each arrow ${\bar{V}}_{i}\to {\bar{V}}_{j}$ , which indicates a causal connection in the system generating the variables. The structure of edges in the graph removing arrowheads is called the skeleton of the graph. The graph is acyclic because we consider causal mechanisms not to be instantaneous and any causal cycle spans in time.

A path in *G* is a sequence of (at least two) distinct nodes ${\bar{V}}_{1},\ldots ,{\bar{V}}_{m},$ such that there is an edge between ${\bar{V}}_{k}$ and ${\bar{V}}_{k+1}$ for all k=1,…,m−1 . If all edges are directed as ${\bar{V}}_{k}\to {\bar{V}}_{k+1}$ the path is a causal or directed path. A node ${\bar{V}}_{i}$ is a collider in a path if it has incoming arrows ${\bar{V}}_{i-1}\to {\bar{V}}_{i}\leftarrow {\bar{V}}_{i+1}$ and is a noncollider otherwise. If there is an arrow ${\bar{V}}_{i}\to {\bar{V}}_{j}$ , then ${\bar{V}}_{i}$ is a parent of ${\bar{V}}_{j}$ , and ${\bar{V}}_{j}$ is a child of ${\bar{V}}_{i}$ . A node ${\bar{V}}_{i}$ is called an ancestor of ${\bar{V}}_{j}$ if there is a directed path from ${\bar{V}}_{i}$ to ${\bar{V}}_{j}$ . Conversely, in this case ${\bar{V}}_{j}$ is a descendant of ${\bar{V}}_{i}$ . We use bidirected arcs ${\bar{V}}_{i}↔{\bar{V}}_{j}$ to indicate the presence of a nondirected path between ${\bar{V}}_{i}$ and ${\bar{V}}_{j}$ consisting only of hidden noncolliders.

A causal graph accurately represents the generative mechanisms of a system when a variable ${\bar{V}}_{i}$ is a parent of another variable ${\bar{V}}_{j}$ if and only if it is an argument of an underlying functional equation that captures the mechanisms that generate ${\bar{V}}_{j}$ . This creates a relation between the conditional independencies that hold between variables in the system and a graphical criterion of separability between the nodes, called *d-separation* [32]. The criterion of d-separation states that two nodes *X* and *Y* are *d-separated* given a set of nodes S if and only if no S -active paths exist between *X* and *Y*. A path is active given the conditioning set S ( S -active) if no noncollider in the path belongs to S and every collider in the path either is in S or has a descendant in S . A causal structure *G* and a generated probability distribution $p(\bar{V})$ are *faithful* [1,2] to one another when a conditional independence between *X* and *Y* given S –denoted by $X{⊥}_{P}Y|S$ – holds if and only if *X* and *Y* are d-separated given S –denoted by $X{⊥}_{G}Y|S$ .

The inference of the causal structure of a system from data generated from the system relies on this link between the causal structure and independencies. Causal learning algorithms that use conditional independencies to reconstruct a partially oriented graph [1,2,4] rely on the assumption of faithfulness in order to determine the skeleton of the graph and to apply rules of orientation of the edges. On the other hand, in the case that causally informative inequalities are used to rule out causal structures [14,24,26], it is only required to assume the substantially weaker assumption that d-separability implies conditional independence. Under this assumption, if a causal structure implies the set of independencies that lead to the fulfillment of the inequality, its violation allows discarding that causal structure. The assumption of faithfulness is not required because if unfaithful independencies are present in the data, which do not follow from the causal structure, this may decrease the power to reject causal structures, but does not lead to incorrect rejections.

Note that the assumption that graphical separability implies statistical conditional independence is substantially weaker than the converse assumption that statistical conditional independence implies graphical separability. A counterexample of the latter is the X-OR logical gate. On the other hand, if the causal graph reflects the underlying structure of mechanisms involved in generating the variables, all statistical dependencies need to originate from some paths of influence between the variables. If some variables are conditionally dependent while the graph indicates that they are d-separated, then the graph must be misrepresenting the paths that create the observed dependence.

### 2.2. The Data Processing Inequality

The data processing inequality (DP inequality) of mutual information indicates that information cannot be increased in a Markov chain [27].

**Lemma 1 ** 
(Data processing inequality of conditional mutual information). *Let $\bar{Z}$ , D , $D^{\prime }$ , and E be four nonoverlapping sets of variables. If $\bar{Z}⊥D^{\prime }|DE$ , then it follows that $I\left(\bar{Z};D,D^{\prime }|E\right)=I\left(\bar{Z};D|E\right)\geq I\left(\bar{Z};D^{\prime }|E\right)$ .*

While the DP inequality is often used only to refer to the inequality between the information carried by D and $D^{\prime }$ , we will also apply the equality of the information carried by $\left\{D,D^{\prime }\right\}$ and D alone.

### 2.3. The Instrumental Entropic Inequality

**Figure 1:**
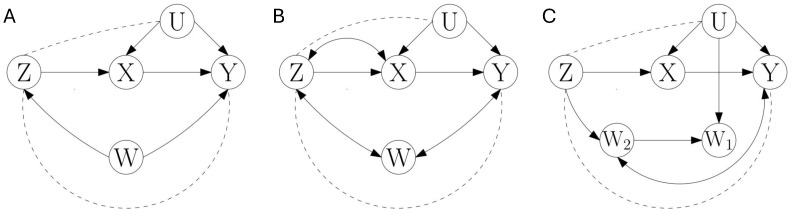
Examples of causal structures within the instrumental inequality scenario. All variables are observable except *U* hidden. Each graph represents several causal structures, corresponding to instantiations of the dashed edges under the constraints of acyclic graphs. Instantiations comprise arrows in one or the other direction, a bidirectional arc indicating the presence of a hidden parent, a combination of an arrow and bidirectional arc, or the removal of the dashed edge, corresponding to the lack of a direct connection. (**A**) Example of the standard instrumental entropic inequality (Section 2.3). (**B**) Example of the instrumental entropic inequality with a term of unique information (Section 3.1). (**C**) Example in which an instrumental entropic inequality with unique information provides causal inference power additional to the one obtainable from standard instrumental entropic inequalities (Section 3.2).

We here revise the instrumental entropic inequality [24]. We provide its full derivation because this helps to identify how new entropic inequalities can be derived. Consider the causal structures of Figure 1A, with all variables observable except *U* hidden. We use a notation of the variables consistent with the role of *Z*, *X*, *Y*, and *U* in the work that introduced the instrumental inequality [19] and its entropic formulation [24]. The diagram represents several causal structures, depending on how the dashed edges are instantiated (or removed) as additional causal connections. These additional connections are constrained by the acyclic nature of the causal graph, for example Z→Y or Z↔Y are valid, while Z←Y is not, since it would lead to the existence of a cycle. For all the causal structures of Figure 1A, no conditional independencies between variables in {X,Y,Z} exist that involve conditioning only on observable variables. Accordingly, the reconstruction of the causal structure based on conditional independencies, for example using the PC algorithm of Spirtes et al. [1], results in all cases in a reconstructed graph in which nodes {X,Y,Z} are all connected. In particular, also for the causal structure in which the dashed edges are removed, a reconstructed edge Z−Y is obtained, even if not present in the actual skeleton of that causal structure. This is due to the fact that blocking the path Z→X→Y by conditioning on *X* activates the path Z→X←U→Y .

The instrumental entropic inequality provides a causally informative test to reject the compatibility of a data set with this causal structure in which the dashed edges are not present. Even if no independencies between {Z,X,Y} exist in the marginal scenario in which *U* is hidden, this causal structure contains untestable independencies that involve the hidden variable *U*, comprising Z⊥U|W and Z⊥Y|UXW . These untestable independencies impose additional constraints that manifest themselves in an inequality between information terms, namely the instrumental entropic inequality. For later convenience, we formulate the standard instrumental entropic inequality allowing for a multivariate Z :

**Proposition 1 ** 
(Instrumental entropic inequality). *Consider the variables Z , X, Y, $B_{0}$ , and U, all observable except U a hidden variable. Consider that the causal structure is such that, for all $Z_{i}\in Z$ , no pair from $\left\{Z_{i},X,Y\right\}$ is separable given that U is hidden. Consider that the causal structure imposes the existence of the nontestable independencies $Z⊥U|B_{0}$ and $Z⊥Y|UXB_{0}$ . These independencies result in the testable inequality*

(1) $$H\left(X|B_{0}\right)\geq I\left(Z;X|B_{0}\right)+I\left(Z;Y|B_{0},X\right).$$

**Proof. **  The mutual information $I(Z;U,X|B_{0})$ can be decomposed applying the chain rule in two alternative orders. If information with *U* is considered first

(2) $$\begin{array}{c} I\left(Z;U,X|B_{0}\right)\overset{\left(a\right)}{=}I\left(Z;U|B_{0}\right)+I\left(Z;X|B_{0},U\right)\overset{\left(b\right)}{\leq }H\left(X|B_{0}\right). \end{array}$$

Equality (a) applies the chain rule of mutual information. Inequality (b) holds because $I(Z;U|B_{0})=0$ , given the independence $Z⊥U|B_{0}$ , and by definition $I(Z;X|B_{0},U)$ is smaller than or equal to $H(X|B_{0},U)$ , which by monotonicity of entropy under conditioning is smaller than or equal to $H(X|B_{0})$ . Considering now the chain rule with *X* first,

(3) $$\begin{array}{c} I\left(Z;U,X|B_{0}\right)\overset{\left(a\right)}{=}I\left(Z;X|B_{0}\right)+I\left(Z;U|B_{0},X\right)\overset{\left(b\right)}{\geq }I\left(Z;X|B_{0}\right)+I\left(Z;Y|B_{0},X\right). \end{array}$$

Equality (a) applies the chain rule of mutual information. Inequality (b) holds because $Z⊥Y|UXB_{0}$ implies the DP inequality $I\left(Z;U|B_{0},X\right)\geq I\left(Z;Y|B_{0},X\right)$ . Combining the upper bound $H(X|B_{0})$ and the lower bound $I\left(Z;X|B_{0}\right)+I\left(Z;Y|B_{0},X\right)$ proves the testable inequality. □

The instrumental entropic inequality holds in Figure 1A with, Z=Z and $B_{0}=W$ . It equally holds with changes in Z−W−Y , as long as *W* is a noncollider. Note that while the instrumental inequality gets its name from the possibility to use *Z* as a *causal intervention instrument* that can be manipulated (intervened) to estimate the causal effect that *X* has on *Y* [20,33,34] in fact the inequality is equally fulfilled with Z↔X , since this does not alter the independence Z⊥U|W . That is, the instrumental inequality also holds for causal structures where *Z* is not a causal intervention instrument. Since in this work we study causal structure learning and not the identification of causal effects, we will refer to a set of variables as a *causal discovery instrumental set* purely based on the fulfillment of the independence $Z⊥U|B_{0}$ . The reason not to involve the independence $Z⊥Y|UXB_{0}$ in this criterion will become clear in Section 3.2. Furthermore, since there is no possible confusion within this work, we will abbreviate *causal discovery instrumental set* simply as *instrumental set*.

Proposition 1 states a straightforward extended version of the basic instrumental entropic inequality in the sense that it comprises a multivariate Z . We will refer to this inequality as the *standard instrumental entropic inequality*. This multivariate version will be needed for comparison with the new types of instrumental entropic inequalities we will introduce. Note that we purposely have excluded further straightforward generalizations, such as a multivariate X , Y , and U . We will add more generalizations in the Results section, but this simple version is suited to identify the key components of the inequality and its relation to the causal structure, as will be examined in Section 3.2.

Importantly, a causal structure that fulfills the nontestable independencies $Z⊥U|B_{0}$ and $Z⊥Y|UXB_{0}$ imposes the fulfillment of the inequality of Proposition 1 to any data set generated from that causal structure. In that case, we will say that the inequality is *causally* fulfilled. On the other hand, for a data set generated with another causal structure, the entropic inequality may equally be fulfilled, even if its fulfillment was not imposed by the causal structure. In that case, we will say that the inequality is *statistically* fulfilled. The causal inference power of an inequality emanates from the possibility to discard a causal structure that imposes the causal-fulfillment of the inequality when the violation of the inequality is verified from data. As mentioned in Section 2.1, in order to be able to reject causal structures based on the violation of causally informative inequalities, we will work under the assumption that causal separability (d-separation) implies statistical conditional independence. That is, we assume that for a causal structure that causally imposes an inequality, the inequality indeed is fulfilled because the causal structure creates the independencies that lead to the inequality.

### 2.4. Constrained Minimum Mutual Information and Maximum Entropy Unique Information

Problems of constrained optimization of information-theoretic quantities within families that share marginal distributions often appear in the study of communication channels [35]. Furthermore, minimum information methods have been proposed for machine learning and signal processing tasks [36,37] as a generalization of the maximum entropy principle [38]. As developed in the Results below, our extension of causally informative entropic inequalities relies on the use of minimum information (minInf) terms that are defined within families of distributions that share sets of marginals with the original joint distribution.

In general, minimization constraints can comprise both inequality and equality equations that are imposed to joint distributions of variables. A set of constraints determines a family ΔP of probability distributions within which the mutual information term of interest is to be minimized, namely among all distributions compatible with the fulfillment of the constraints. In this work, we focus on minInf terms defined within families of distributions that preserve sets of marginals of the joint distribution associated with a data set. In general, given a joint distribution $P(\bar{V})$ for $\bar{V}$ variables, a minInf term is defined as

(4) $$\min_{Q\in \Delta P}I_{Q}\left({\bar{V}}_{1};{\bar{V}}_{2}|{\bar{V}}_{3}\right),$$

where ${\bar{V}}_{i}$ , i=1,…,3 are subsets of $\bar{V}$ and ΔP is the family of distributions determined by the set of constraints imposed to the distributions. Accordingly, the minimization within the family corresponds to a constrained minimization subject to the constraints that define the family. When the constraints impose the preservation of a set of marginal distributions of the original $P(\bar{V})$ , that is, when Q∈ΔP is subject to $Q\left({\bar{V}}_{S}\right)=P\left({\bar{V}}_{S}\right)$ for a certain number of subsets *S* of $\bar{V}$ , then the constraints constitute a set of affine linear equality constraints on the joint distribution $Q(\bar{V})$ .

In this work we prove some general properties of minInf information terms that render them useful for the derivation of more powerful causally informative testable entropic inequalities. These properties include a data processing inequality for minInf terms (Proposition 5) and a procedure to iteratively combine minInf data processing inequalities (Theorem 1). We then use these properties for the construction of some specific entropic inequalities, such as extended instrumental entropic inequalities. To the best of our knowledge, although the use of minimum information quantities appears in the study of communication channels and has been formulated in machine learning problems as mentioned above, the properties we introduce have not been derived before, and the use of minInf terms to derive causally informative entropic inequalities is new.

As a precedent to this work, Chicharro and Nguyen [26] showed how to apply to causal structure learning a measure of maximum entropy unique information previously introduced in [29]. Maximum entropy measures correspond to a specific subcase of minInf terms of the form of Equation (4). Concretely, when $P({\bar{V}}_{1},{\bar{V}}_{3})$ is among the preserved marginals in ΔP , then the entropy $H_{Q}\left({\bar{V}}_{1}|{\bar{V}}_{3}\right)$ is fixed, and the minimization of $I_{Q}\left({\bar{V}}_{1};{\bar{V}}_{2}|{\bar{V}}_{3}\right)$ is equivalent to the maximization of $H_{Q}\left({\bar{V}}_{1}|{\bar{V}}_{2},{\bar{V}}_{3}\right)$ .

We now revise the definition of the maximum entropy unique information [29] and the relevant properties derived in Chicharro and Nguyen [26]. This will serve as reference to generalize more general data processing inequalities for minInf terms. The concept of *unique information* was originally introduced [30] as part of a nonnegative decomposition of the joint mutual information that a set of *predictor* variables has about a *target* variable $\bar{Z}$ . In the simplest scenario with two (possibly multivariate) predictors $\left\{D_{1},D_{2}\right\}$ , the unique information of predictor $D_{i}$ with respect to the *reference* predictor $D_{j}$ quantifies the exclusive information about $\bar{Z}$ obtained from $D_{i}$ and not from $D_{j}$ . The other components of the decomposition quantify redundant and synergistic information terms. Alternative formulations of this decomposition of mutual information have been introduced, e.g., [29,39,40,41], using definitions alternative to the maximum entropy formulation of unique information of [29]. However, the application of the maximum entropy unique information measure to causal inference [26] does not rely on its embedding within the framework of [30], but only on certain properties that render it useful to derive testable causally informative information inequalities. We here revise its definition and these properties. In general, we will use *unique information* to refer concretely to the maximum entropy unique information.

While the measure was originally introduced in the bivariate unconditional case [29], we here revise the conditional unique information measure as presented in [26]. For sets of variables $\bar{Z}$ , $D_{1}$ , $D_{2}$ , and $O_{1}$ , the unique information of *predictor* $D_{1}$ with respect to the *reference* $D_{2}$ about the *target* $\bar{Z}$ , conditioning on $O_{1}$ , is defined as

(5) $$I(\bar{Z};D_{1}\backslash \backslash D_{2}\left|O_{1}\right)\equiv \min_{Q\in \Delta P}I_{Q}\left(\bar{Z};D_{1}|E\right),$$

where $E\equiv \{D_{2},O_{1}\}$ , and ΔP is the family of distributions on $\left\{\bar{Z},D_{1},D_{2},O_{1}\right\}$ that preserve the marginals $P(\bar{Z},D_{1},O_{1})$ and $P(\bar{Z},D_{2},O_{1})$ of the original $P(\bar{Z},D_{1},D_{2},O_{1})$ . The notation $I_{Q}$ is used to indicate that the mutual information is calculated on the probability distribution *Q*. We use $I(\bar{Z};D_{1}\backslash \backslash D_{2}|O_{1})$ to refer to the unique information of $D_{1}$ with reference $D_{2}$ , conditioning on $O_{1}$ , compared to $I(\bar{Z};D_{1}|D_{2},O_{1})$ , which is the standard conditional information of $D_{1}$ conditioning on $\left\{D_{2},O_{1}\right\}$ . Note that the constraints on ΔP are such that they divide the conditioning set E into the variables $O_{1}$ included in $P(\bar{Z},D_{1},O_{1})$ and the variables $D_{2}$ , that appear only in the marginal $P(\bar{Z},D_{2},O_{1})$ , where $D_{1}$ is excluded. We enumerate $O_{1}$ with subindex 1 because when dealing with more general minInf terms in Section 3.4 we will distinguish multiple subsets of E preserved in different marginal distributions. We use the notation $D_{1}\backslash \backslash D_{2}$ instead of the notation $D_{1}\backslash D_{2}$ originally introduced by [29] to differentiate it from the set notation $D_{1}\backslash D_{2}$ , which indicates the subset of variables in $D_{1}$ that is not contained in $D_{2}$ . The measure defined in Equation (5) is equivalently a maximum entropy measure because the information minimization can equally be formulated as an entropy maximization, since all distributions within ΔP preserve the conditional entropy $H(\bar{Z}|D_{2},O_{1})$ .

Several properties are important for its use into causally informative inequalities. First, by construction [29], the conditional unique information is bounded as

(6) $$\min\left\{I\left(\bar{Z};D_{1}|O_{1}\right),I\left(\bar{Z};D_{1}|D_{2},O_{1}\right)\right\}\geq I(\bar{Z};D_{1}\backslash \backslash D_{2}\left|O_{1}\right)\geq 0.$$

Second, the unique information is monotonic in the predictor argument:

**Lemma 2 ** 
(Monotonicity of unique information in the predictor argument). *The maximum entropy conditional unique information is monotonic on its second argument, corresponding to the non-referent predictor:*

(7) $$I(\bar{Z};D,D^{\prime }\backslash \backslash D_{2}|O_{1})\geq I(\bar{Z};D\backslash \backslash D_{2}\left|O_{1}\right).$$

In relation to Equation (5), $D_{1}=\left\{D,D^{\prime }\right\}$ . This property was derived in Lemma 3 of [42] for the unconditional case and extended to the conditional case in Lemma 2 of [26]. The proof is provided in Appendix A.

Third, a DP inequality was derived in Chicharro and Nguyen [26] for the maximum entropy unique information:

**Lemma 3 ** 
(Conditional unique information data processing inequality). *Let $\bar{Z}$ , D , $D^{\prime }$ , $D_{2}$ , and $O_{1}$ be five nonoverlapping sets of variables. If $I(\bar{Z};D^{\prime }|D,O_{1})=0$ , then $I(\bar{Z};D,D^{\prime }\backslash \backslash D_{2}|O_{1})=I(\bar{Z};D\backslash \backslash D_{2}|O_{1})\geq I(\bar{Z};D^{\prime }\backslash \backslash D_{2}\left|O_{1}\right)$ .*

Again, the proof is provided in Appendix A to serve as guidance for posterior extensions. This DP inequality is analogous to the standard DP inequality of the mutual information (Lemma 1), except that it requires the independence $\bar{Z}⊥D^{\prime }|DO_{1}$ instead of $\bar{Z}⊥D^{\prime }|DE$ , with $E=\{D_{2},O_{1}\}$ . Note that all the variables involved in the independence $\bar{Z}⊥D^{\prime }|DO_{1}$ are included in the marginal $P(\bar{Z},D^{\prime },D,O_{1})$ preserved in the family ΔP that defines $I(\bar{Z};D,D^{\prime }\backslash \backslash D_{2}|O_{1})$ .

## 3. Results

To develop how minInf terms can be used in causally informative entropic inequalities, we start from the scenario of the standard instrumental entropic inequality (Figure 1A) and consider changes in the causal structure. We first derive (Section 3.1) an instrumental entropic inequality that applies the DP inequality of unique information. We then address the embedding of this new type of inequalities together with standard instrumental inequalities derived with multivariate instrumental sets and we illustrate that they can provide additional causal inference power (Section 3.2). In Section 3.3, we examine instrumental entropic inequalities in which the DP inequality of conditional mutual information and unique information are combined. This analysis reveals how different types of DP inequalities can recursively be applied. In Section 3.4, we introduce a type of DP inequalities for minInf terms, which encompasses as subcases the DP inequalities of conditional mutual information and unique information. We show how to recursively apply these DP inequalities to obtain sums of observable information terms as lower bounds of unobservable information terms. In Section 3.5, we apply this procedure to construct more powerful instrumental entropic inequalities. In Section 3.6, we reexamine more broadly the derived minInf inequalities from a geometrical perspective, in connection with Shannon entropy cones. Finally, in Section 3.7, we apply the procedures developed in Section 3.4 to other types of entropic inequalities beyond the instrumental inequality scenario.

### 3.1. Instrumental Entropic Inequalities with Maximum Entropy Unique Information Terms: The Case with One Data Processing Inequality Applied

We start considering how to construct instrumental entropic inequalities with the causal structures of Figure 1B. Again, the graph displays several causal structures depending on the instantiation of the dashed edges. Similar to the case of Figure 1A, a requirement for any instrumental entropic inequalities to be causally fulfilled is that the dashed edges between *Z* and *Y* as well as between *Z* and *U* are removed. Therefore, we focus on this case with no edges between *Z* and *Y* and between *Z* and *U*. A difference between Figure 1A,B is that *W* is a noncollider in Figure 1A, leading to Z⊥Y|UX and Z⊥Y|UXW while *W* is a collider in Figure 1B, such that Z⊥Y|UX and Z⊥Y|UXW . For Figure 1B, the instrumental inequality of Proposition 1 can be applied with Z=Z and $B_{0}=\emptyset$ . The required independencies $Z⊥U|B_{0}$ and $Z⊥Y|UXB_{0}$ are fulfilled, namely they correspond to Z⊥U and Z⊥Y|UX . This leads to

(8) H(X)≥I(Z;X)+I(Z;Y|X).

On the contrary, the fact that *W* is a collider leads to a dependence Z⊥Y|UXW , and hence in Figure 1B Proposition 1 cannot be applied selecting $B_{0}=W$ . Note that being able to condition on $B_{0}=W$ would be advantageous because, following the derivation of Proposition 1, it would lead to a tighter upper bound H(X|W) instead of H(X) . Since what prevents deriving an instrumental entropic inequality with $B_{0}=W$ is that Z⊥Y|UXW , as opposed to Z⊥Y|UX , we can consider if using the unique information DP inequality is useful in this case. This is because the unique information has a DP inequality (Lemma 3) that differs from the one of conditional mutual information (Lemma 1) in that it is associated with a conditional independence that excludes the reference variables from the conditioning set. This type of exclusion is precisely what is needed to use Z⊥Y|UX instead of Z⊥Y|UXW . We first state a general formulation of an instrumental entropic inequality that uses the unique information and we will then go back to the example of Figure 1B.

**Proposition 2 ** 
(Instrumental entropic inequality with maximum entropy unique information). *Consider the variables Z , X, Y, $B_{0}$ , and U, all observable except U a hidden variable. Consider that the causal structure is such that, for all $Z_{i}\in Z$ , no pair from $\left\{Z_{i},X,Y\right\}$ is separable given that U is hidden. Consider an exclusive partition $B_{0}=\left\{B_{1},B_{2}\right\}$ . Consider that the causal structure imposes the nontestable independencies $Z⊥U|B_{0}$ and $Z⊥Y|UXB_{1}$ . These independencies result in the testable inequality*

(9) $$H\left(X|B_{0}\right)\geq I\left(Z;X|B_{0}\right)+I(Z;Y\backslash \backslash B_{2}\left|B_{1},X\right).$$

**Proof. **  The proof is analogous to the one of Proposition 1. Again, the departing quantity is $I(Z;U,X|B_{0})$ . Using the chain rule to decompose $I(Z;U,X|B_{0})$ as the sum of $I(Z;U|B_{0})$ and $I(Z;X|B_{0},U)$ , the independence $Z⊥U|B_{0}$ allows deriving $H(X|B_{0})$ as upper bound, as in Equation (2). For the lower bound, instead of Equation (3) that applies the DP inequality of conditional information, the DP inequality of unique information is applied:

(10) $$\begin{array}{cc} I\left(Z;U,X|B_{0}\right)\overset{\left(a\right)}{=} & I\left(Z;X|B_{0}\right)+I\left(Z;U|B_{0},X\right)\overset{\left(b\right)}{\geq }I\left(Z;X|B_{0}\right)+I(Z;U\backslash \backslash B_{2}\left|B_{1},X\right)\overset{\left(c\right)}{\geq }\\ & I\left(Z;X|B_{0}\right)+I(Z;Y\backslash \backslash B_{2}\left|B_{1},X\right). \end{array}$$

Equality (a) applies the chain rule of mutual information. Inequality (b) applies the definition of the unique information as a contribution smaller than or equal to the conditional mutual information (Equation (6)). Inequality (c) holds because $Z⊥Y|UXB_{1}$ by Lemma 3 implies the DP inequality of the unique information $I(Z;U\backslash \backslash B_{2}|B_{1},X)\geq I(Z;Y\backslash \backslash B_{2}\left|B_{1},X\right)$ . Combining the upper bound $H(X|B_{0})$ and the lower bound $I\left(Z;X|B_{0}\right)+I(Z;Y\backslash \backslash B_{2}\left|B_{1},X\right)$ proves the testable inequality. □

In the example of Figure 1B, Proposition 2 applies with Z=Z , $B_{1}=\emptyset$ , and $B_{2}=W$ and results in

(11) H(X|W)≥I(Z;X|W)+I(Z;Y\\W|X).

The inequality of Equation (11) is causally imposed when the causal structure creates the independencies Z⊥U|W and Z⊥Y|UX . These independencies would also exist if in Figure 1B variables *X* and *W* were connected by an arc X↔W . On the other hand, W→X would produce Z⊥Y|UX , and X→W would produce Z⊥U|W .

Comparing Equations (8) and (11), the first is derived with $B_{0}=\emptyset$ and the second with $B_{0}=W$ , $B_{1}=\emptyset$ , and $B_{2}=W$ . To better appreciate the factors that determine their power, we can rewrite them passing the first term at the r.h.s. to the l.h.s.:

(12a) $$\begin{array}{cc} \; & H(X|Z)\geq I(Z;Y|X) \end{array}$$

(12b) $$\begin{array}{cc} \; & H(X|Z,W)\geq I(Z;Y\backslash \backslash W|X). \end{array}$$

In general, these inequalities are complementary. For example, consider that it is to be tested the compatibility of a data set with the causal structure in Figure 1B with no edge Z−U and no edge Z−Y . This causal structure creates independencies Z⊥U , Z⊥U|W , and Z⊥Y|UX , and hence causally imposes both inequalities of Equations (8) and (11). Therefore, the violation of any of the two inequalities suffices to discard the causal structure. Comparing their form, Equation (12b) has a smaller or equal upper bound than Equation (12a), given the monotonicity of entropy under conditioning. However, it also has a smaller or equal lower bound, since the unique information is upper-bounded by the unconditional mutual information (Equation (6)). This means that, for a concrete data set that has been generated from another causal structure that does not impose the fulfillment of the inequalities, any of the two inequalities can be violated while the other is not, so that their use is complementary for causal inference. Using Z⊥U|W instead of Z⊥U allows decreasing the upper bound, but when *W* is a collider between *Z* and *Y* such that Z⊥Y|UXW , a smaller observable lower bound is derived with the unique information and Z⊥Y|UX .

### 3.2. Instrumental Entropic Inequalities with Multivariate Instrumental Sets

So far, the example of Figure 1B presented an application of Proposition 2 with a univariate instrument Z=Z . However, to establish that the new type of inequalities of Proposition 2 contributes additional causal inference power to the standard instrumental entropic inequalities, we also need to examine the standard instrumental inequalities with multivariate instrumental sets that exist for the same causal structure. To do so, we first highlight three key elements of the structure of instrumental entropic inequalities.

The derivation of both Propositions 1 and 2 departs from the quantity $I(Z;U,X|B_{0})$ . The first key element is that the two required types of independencies play separate roles in the derivation of instrumental inequalities: $Z⊥U|B_{0}$ is used to derive the observable upper bound, while the other independence is used to derive the observable lower bound. In more detail, $Z⊥U|B_{0}$ is used after $I(Z;U,X|B_{0})$ is separated into $I(Z;U|B_{0})$ and $I(Z;X|B_{0},U)$ . The other required independence is used to derive the observable lower bound thanks to a DP inequality, which is applied to $I(Z;U|B_{0},X)$ . The different DP inequality applied is what differentiates Propositions 1 and 2.

The second key element is better appreciated rewriting the inequalities of Propositions 1 and 2 passing the the first term of the r.h.s. to the l.h.s.:

(13a) $$\begin{array}{cc} \; & H\left(X|Z,B_{0}\right)\geq I\left(Z;Y|B_{0},X\right) \end{array}$$

(13b) $$\begin{array}{cc} \; & H\left(X|Z,B_{0}\right)\geq I(Z;Y\backslash \backslash B_{2}\left|B_{1},X\right). \end{array}$$

Written like this, the two inequalities have upper bound $H(X|Z,B_{0})$ , with a conditioning set $\left\{Z,B_{0}\right\}$ that does not differentiate between Z and $B_{0}$ , which appear in different arguments of $Z⊥U|B_{0}$ . Therefore, regarding the upper bound, there is an invariance under the exchange of variables between the *causal discovery instrumental set* Z and the conditioning set $B_{0}$ . Alternative instrumental entropic inequalities that would require independencies $\left\{Z,B_{4}\right\}⊥U|B_{3}$ , with $B_{0}=\left\{B_{3},B_{4}\right\}$ , would all lead to the same upper bound $H(X|Z,B_{0})$ . Accordingly, instrumental inequalities with multivariate instrumental sets obtained under this invariance need to be considered in order to assess the additional causal inference power provided by the new type of inequalities introduced in Proposition 2.

We can see an example of multivariate instrumental set in Figure 1B, again focusing on the causal structure with no connections Z−U and Z−Y . Variable *W*, which in the derivation of Equation (12b) is assigned to $B_{0}=W$ , can be assigned to the instrumental set, leading to the bivariate instrumental set Z={Z,W} and to $B_{0}=\emptyset$ . The set {Z,W} fulfills $Z⊥U|B_{0}$ , namely {Z,W}⊥U . On the other hand, {Z,W} does not fulfill the other independence condition $Z⊥Y|UXB_{0}$ required in Proposition 1, since {Z,W}Y|UX due to the direct connection between *W* and *Y*.

This leads us to the third key element of the construction of instrumental entropic inequalities. In Propositions 1 and 2, there is a single set Z that appears in both independencies, namely $Z^{\prime }=Z$ in either $Z⊥U|B_{0}$ and $Z^{\prime }⊥Y|UXB_{0}$ , or in $Z⊥U|B_{0}$ and $Z^{\prime }⊥Y|UXB_{1}$ . However, this constraint is not necessary and can be relaxed. In the derivation of an observable lower bound, a DP inequality could be applied to any subset $Z^{\prime }\subseteq Z$ . This is captured in the following Proposition. For later convenience, we now also consider multivariate variables X , Y , and U :

**Proposition 3 ** 
(Chainlike instrumental entropic inequalities with multivariate instrumental sets). *Consider variables Z , X , Y , $B_{0}$ , and U , all observable except U hidden variables. Consider that the causal structure is such that for at least a $Z_{i}\in Z$ there is a nonempty subset $X_{i}\in X$ and $Y_{i}\in Y$ such that no pair in $\left\{Z_{i},X_{i},Y_{i}\right\}$ is separable with U hidden. Consider an exclusive partition in r parts of the multivariate instrumental set Z given by $Z^{\left[r\right]}=\left\{Z_{0},Z_{1},\ldots ,Z_{r}\right\}$ , with $Z_{0}=\emptyset$ . Consider that the causal structure imposes the nontestable independence $Z⊥U|B_{0}$ . This independence creates a nontestable instrumental entropic inequality*

(14) $$\begin{array}{c} H\left(X|B_{0}\right)\geq I\left(Z;X|B_{0}\right)+I\left(Z;U|B_{0},X\right)=I\left(Z;X|B_{0}\right)+\sum_{j=1}^{r}I\left(Z_{j};U|B_{0},X,Z^{\left[j-1\right]}\right), \end{array}$$

*with $Z^{\left[j-1\right]}=\left\{Z_{0},Z_{1},\ldots ,Z_{j-1}\right\}$ , where nontestability is due to the nonestimable components of the lower bound. A nontrivial testable instrumental entropic inequality exists if for at least one term $I(Z_{j};U|B_{0},X,Z^{\left[j-1\right]})$ at least one conditional independence exists that enables a data processing inequality to substitute that term by an estimable lower bound that contains some variables in Y and does not contain U .*

**Proof. **  The derivation of the upper bound is the same as in Propositions 1 and 2. Starting from $I(Z;U,X|B_{0})$ the chain rule is applied and the upper bound derived with $I(Z;U|B_{0})=0$ thanks to $Z⊥U|B_{0}$ . The nonestimable lower bound follows from a direct application of the chain rule equality of conditional mutual information to separate {U,X} into the terms $I(Z;X|B_{0})$ and $I(Z;U|B_{0},X)$ , followed by the chain rule to separate Z with the partition $Z^{\left[r\right]}$ . Finally, the proposition states that it suffices that at least one term of the sum in the lower bound can be replaced by at least one observable information term so that a nontrivial testable inequality is obtained, dropping all remaining terms in the lower bound that contain hidden variables. This replacement is possible when applying at least one DP inequality to at least a term $I(Z_{j};U|B_{0},X,Z^{\left[j-1\right]})$ . Of course, a testable instrumental inequality is also obtained if more than one term can be replaced by observable lower bounds. □

Note that Proposition 3 does not specify the form of the conditional independencies and associated DP inequalities applied to obtain observable lower bounds. New procedures to do so are to be specified in Section 3.4. The advantage of this formulation is that it distinguishes between the variables Z that appear in the condition $Z⊥U|B_{0}$ and the subsets $Z_{j}$ that are involved in the independencies associated with DP inequalities to derive observable terms in the lower bound. This explains why in Section 2.3 we defined *causal discovery instrumental sets* based only on $Z⊥U|B_{0}$ .

We can now reconsider the multivariate instrumental set of Figure 1B that assigns Z={Z,W} and $B_{0}=\emptyset$ . As mentioned, {Z,W}⊥U allows deriving an upper bound H(X|Z,W) . The reason why Proposition 1 could not be applied is that {Z,W}Y|UX due to the direct connection between *W* and *Y*. However, Proposition 3 can be applied to the example of Figure 1B selecting a partition with r=2 , $Z^{\left[2\right]}=\left\{\emptyset ,\left\{Z\right\},\left\{W\right\}\right\}$ . Then I(Z,W;U|X) is decomposed into I(Z;U|X)+I(W;U|X,Z) . It now suffices to apply the DP inequality based on Z⊥Y|UX to obtain a lower bound I(Z;U|X)≥I(Z;Y|X) , and hence to obtain the instrumental entropic inequality

(15) H(X)≥I(Z,W;X)+I(Z;Y|X).

The test with this inequality subsumes both tests of Equations (8) and (11), derived from Propositions 1 and 2, respectively. This is because it can be rewritten as H(X|Z,W)≥I(Z;Y|X) , and hence combines the upper bound of Equation (12b) and the lower bound of Equation (12a).

The consideration of instrumental inequalities with multivariate instrumental sets discards that the new type instrumental entropic inequality of Proposition 2 provides additional causal inference power in the case of Figure 1B. More generally, we show in Appendix J that for a multivariate instrumental set Z that fulfills an independence $Z⊥U|B_{0}$ , no additional power can be gained from tests that use only a subset of Z as instrumental set, removing the rest of variables or transferring some variables of Z into the conditioning set $B_{0}$ . However, a causal structure may be such that for $Z=\{Z_{1},Z_{2}\}$ it holds $Z_{2}⊥U|B_{0}Z_{1}$ and yet Z⊥U|B0 . Or with the opposite perspective, using the notation of Proposition 2 with $B_{0}=\left\{B_{1},B_{2}\right\}$ , the causal structure may be such that $Z⊥U|B_{0}$ , but $\left\{Z,B_{2}\right\}U|B_{1}$ . In this case, Proposition 2 can add causal inference power.

An example of this is illustrated in Figure 1C. With no direct connection between *Z* and *U*, the independence $Z⊥U|W_{1}W_{2}$ holds. On the other hand, $\left\{Z,W_{1}\right\}U|W_{2}$ , $\left\{Z,W_{2}\right\}U|W_{1}$ , and $\{Z,W_{1},W_{2}\}U$ . If any of these independencies existed, the upper bound $H(X|Z,W_{1},W_{2})$ would be equally obtained from the corresponding multivariate instrumental set, because of the invariance of the upper bound to exchanges between Z and $B_{0}$ . In those cases, the variables of $\left\{W_{1},W_{2}\right\}$ included in the instrumental set could be marginalized instead of requiring the use of a unique information with both variables in the reference argument. However, since these other independencies do not exist, the instrumental inequality constructed from Proposition 2 using $Z⊥U|W_{1}W_{2}$ and Z⊥Y|UX adds additional causal inference power with the test

(16) $$H\left(X|W_{1},W_{2}\right)\geq I\left(Z;X|W_{1},W_{2}\right)+I(Z;Y\backslash \backslash \left\{W_{1},W_{2}\right\}\left|X\right).$$

In this section, we have addressed the issue of whether the new type of entropic inequalities with unique information terms can add causal inference power when tested together with standard instrumental inequalities that use related multivariate instrumental sets. Our objective was to ensure that the new type of inequalities is not trivially subsumed. To do so, we have contemplated two factors that determine when a new inequality test adds causal inference power. First, that there is some hypothesized causal structure of interest that causally imposes the new type of inequality, possibly together with other inequalities. Second, that the form of the new inequality is such that a probability distribution can exist for which the new test is rejected while no other causally-imposed inequality test is simultaneously rejected. On the other hand, if the new inequality is such that when violated also another inequality is always violated, then it does not add additional power. In Appendix B, we provide a full formal statement of the if and only if conditions under which a new entropic inequality test adds additional power to a set of other tests.

By examining multivariate instrumental sets, we have provided an example in Figure 1C for which Proposition 2 provides additional causal inference power. More broadly, we have seen in Proposition 3 that a nontestable entropic inequality is associated with each instrumental set. The identification of a set of variables as an instrumental set implies the existence of an observable upper bound, and the lower bound can be decomposed as a sum of nonobservable information terms. Nontrivial testable entropic inequalities are obtained when finding observable lower bounds of some of these terms. Proposition 3 accommodates the inequalities derived in Propositions 1 and 2. Compared to those, it relaxes the condition of Propositions 1 and 2 that requires that no pair from $\left\{Z_{i},X,Y\right\}$ is separable when *U* is hidden, for all $Z_{i}\in Z$ . This is because Proposition 3 does not cover only the specific application of a type of DP inequality associated with a specific conditional independence, contrarily to Propositions 1 and 2 that are linked to $Z⊥Y|UXB_{0}$ and $Z⊥Y|UXB_{1}$ , respectively. Section 3.3, Section 3.4 and Section 3.5 will generalize procedures to convert nontestable instrumental entropic inequalities into testable ones.

### 3.3. Instrumental Entropic Inequalities with Mutual Information and Maximum Entropy Unique Information Terms: The Case with Two Data Processing Inequalities Applied

We have seen above that, when a single conditional independence is used to derive a lower bound, the unique information DP inequality only increases causal inference power when the variables in the reference argument of the unique information are not part of a valid instrumental set (i.e., no transfer from $B_{2}$ as specified in Proposition 2 to Z creates a valid instrumental set). We now show that this limitation does not occur when two DP inequalities are used to add terms in the lower bound. We show that unique information terms can be added in the lower bound not only instead of conditional information terms, but in addition to them, resulting in an increase of causal inference power. We use this scenario to illustrate the procedure that will then be generalized to combine an arbitrary number of DP inequalities, comprising DP inequalities of conditional mutual information, unique information, and of minInf information terms, as will be introduced in Section 3.4.

**Proposition 4 ** 
(Instrumental entropic inequalities with conditional mutual information and unique information terms). *Consider variables Z , X , $Y=\{Y_{1},Y_{2}\}$ , $B_{0}=\left\{B_{1},B_{2}\right\}$ , and U , all observable except U hidden variables. Consider that the causal structure is such that for at least a $Z_{i}\in Z$ there is a nonempty subset $X_{i}\in X$ and $Y_{i}\in Y$ such that no pair in $\left\{Z_{i},X_{i},Y_{i}\right\}$ is separable when U is hidden. Consider that the causal structure imposes the nontestable independencies $Z⊥U|B_{0}$ , $Z⊥Y_{1}|UXB_{0}$ , and $Z⊥Y_{2}|UXB_{1}Y_{1}\backslash \bar{Y}$ , with $\bar{Y}\subseteq Y_{1}$ . These independencies result in the testable inequality*

(17) $$H\left(X|B_{0}\right)\geq I\left(Z;X|B_{0}\right)+I\left(Z;Y_{1}|B_{0},X\right)+I(Z;Y_{2}\backslash \backslash \left\{B_{2},\bar{Y}\right\}|B_{1},X,Y_{1}\backslash \bar{Y}).$$

**Proof. **  The upper bound is derived as in Proposition 1, given the independence $Z⊥U|B_{0}$ . To derive the lower bound, start with $I(Z;U|B_{0},X)$ after extracting $I(Z;X|B_{0})$ with the chain rule of mutual information:

(18) $$\begin{array}{cc} \; & I\left(Z;U|B_{0},X\right)\overset{\left(a\right)}{=}I\left(Z;U,Y_{1}|B_{0},X\right)\overset{\left(b\right)}{=}I\left(Z;Y_{1}|B_{0},X\right)+I\left(Z;U|B_{0},X,Y_{1}\right)\overset{\left(c\right)}{\geq }\\ \; & I\left(Z;Y_{1}|B_{0},X\right)+I(Z;U\backslash \backslash \left\{B_{2},\bar{Y}\right\}|B_{1},X,Y_{1}\backslash \bar{Y})\overset{\left(d\right)}{\geq }I\left(Z;Y_{1}|B_{0},X\right)+\\ & I(Z;Y_{2}\backslash \backslash \left\{B_{2},\bar{Y}\right\}|B_{1},X,Y_{1}\backslash \bar{Y}). \end{array}$$

Equality (a) applies the DP inequality of conditional mutual information (Lemma 1) thanks to $Z⊥Y_{1}|UXB_{0}$ . Equality (b) applies the chain rule equality of mutual information. Inequality (c) holds from the definition of unique information, which has conditional mutual information as an upper bound (Equation (6)). Inequality (d) applies the DP inequality of unique information with $Z⊥Y_{2}|UXB_{1}Y_{1}\backslash \bar{Y}$ . □

**Figure 2:**
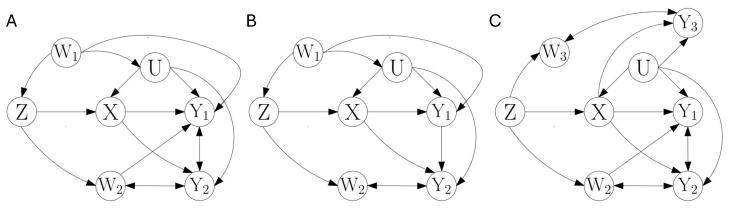
Examples of causal structures that causally impose instrumental entropic inequalities derived by the combination of several types of DP inequalities. All variables are observable except *U* hidden. (**A**,**B**) Examples of causal structures that impose inequalities of the type of Proposition 4, containing conditional mutual information and unique information terms in the lower bound. (**C**) Example in which an instrumental entropic inequality is causally imposed following Proposition 6, containing a sum of minInf terms in its lower bound.

The examples of Figure 2A,B illustrate how the two types of DP inequalities are combined. For simplicity of the explanations, in contrast to Figure 1, in Figure 2 we only represent individual causal structures (no dashed connections). Our objective here is not to derive all existing inequalities in these graphs, but to illustrate the procedure to construct inequalities combining the two types of DP inequalities and to examine the additional causal inference power they can provide. In these causal structures, select the instrumental set Z=Z and conditioning set $B_{0}=\left\{W_{1},W_{2}\right\}$ . With this selection, both causal structures impose inequalities of the type of Proposition 4.

In the example of Figure 2A, given $B_{0}=\left\{W_{1},W_{2}\right\}$ , the upper bound $H(X|W_{1},W_{2})$ is derived with $Z⊥U|W_{1}W_{2}$ . Following Proposition 4, the term with $Y_{1}$ in the r.h.s., $I(Z;Y_{1}|W_{1},W_{2},X)$ , is obtained with $Z⊥Y_{1}|UXW_{1}W_{2}$ , from the mutual information DP inequality. The term with $Y_{2}$ , $I(Z;Y_{2}\backslash \backslash \left\{W_{2},Y_{1}\right\}|W_{1},X)$ , is obtained with $Z⊥Y_{2}|UXW_{1}$ , from the unique information DP inequality. The derivation corresponds to the assignment in Equation (17) of $B_{1}=W_{1}$ , $B_{2}=W_{2}$ , and $\bar{Y}=Y_{1}$ . Note that it is necessary to exclude $\left\{W_{2},Y_{1}\right\}$ from the conditioning set because Z⊥Y2|UXW1S , for any nonempty $S\subseteq \{W_{2},Y_{1}\}$ . In Figure 2B, the upper bound and the term with $Y_{1}$ are derived in the same way. On the other hand, the term $I(Z;Y_{2}\backslash \backslash W_{2}|W_{1},X,Y_{1})$ results from $Z⊥Y_{2}|UXW_{1}Y_{1}$ , with $\bar{Y}=\emptyset$ . The unique information is chosen with reference variable $W_{2}$ as opposed to the reference variable $\left\{W_{2},Y_{1}\right\}$ for Figure 2A. This difference is due to $W_{2}\to Y_{1}$ in Figure 2A, which renders $Y_{1}$ a descendant of the collider $W_{2}$ in $Z-W_{2}-Y_{2}$ , which needs to be removed from conditioning to create an independence. Note that while for simplicity Figure 2A,B show specific causal structures, Proposition 4 also applies under certain changes of these examples. The same inequalities apply in any graph in which arrows are assigned such that $W_{1}$ continues to be a noncollider in $Z-W_{1}\to U$ and $Z-W_{1}\to Y_{1}$ , or if either $W_{1}-U$ or $W_{1}-Y_{1}$ is not present. The graphs could also comprise $W_{1}\to W_{2}$ or $Z-W_{1}\to Y_{2}$ , with $W_{1}$ a noncollider.

We will now illustrate that instrumental inequalities of the type of Proposition 4 can add extra causal inference power. To do so, we show that, for example in Figure 2A, this type of instrumental entropic inequalities is not subsumed by any standard instrumental entropic inequality that uses a multivariate instrumental set. We here justify the additional causal inference power verifying that no standard instrumental inequality can jointly use the DP inequalities that introduce variables $Y_{1}$ and $Y_{2}$ . This guarantees that it does not happen that the new type of instrumental inequality is only violated in cases in which already a standard instrumental inequality is violated. To complement this reasoning, in Appendix D we examine concrete numerical examples in which no rejection occurs for tests based on standard instrumental inequalities, while rejections are obtained when incorporating unique information terms as in Proposition 4.

Consider the multivariate instrumental sets that can be used as an alternative given the selection of the instrumental set Z=Z and conditioning set $B_{0}=\left\{W_{1},W_{2}\right\}$ in Figure 2A,B. We know from Section 3.2 that the same upper bound would hold for any valid instrumental set obtained from a transfer between these sets, namely with Z={Z,S} , and $B_{0}=\left\{W_{1},W_{2}\right\}\backslash S$ , for $S\subseteq \{W_{1},W_{2}\}$ . Given the direct connection between $W_{1}$ and *U*, no set containing $W_{1}$ fulfills the criterion $Z⊥U|B_{0}$ to be an instrumental set. On the other hand, $\left\{Z,W_{2}\right\}⊥U|W_{1}$ , so that $Z=\{Z,W_{2}\}$ is a valid instrumental set with $B_{0}=W_{1}$ .

We now focus specifically on Figure 2A. Starting from $I(Z,W_{2};U|W_{1},X)$ , we examine different partitions of $Z=\{Z,W_{2}\}$ that can be selected to apply Proposition 3. One partition is $Z^{\left[2\right]}=\left\{\emptyset ,\left\{Z\right\},\left\{W_{2}\right\}\right\}$ , which results in terms $I(Z;U|W_{1},X)$ and $I(W_{2};U|W_{1},X,Z)$ . Given that $W_{2}$ has direct connections to $Y_{1}$ and $Y_{2}$ , and hence it is nonseparable from them, no DP inequality can be applied to $I(W_{2};U|W_{1},X,Z)$ . For $I(Z;U|W_{1},X)$ , the DP inequality associated with $Z⊥Y_{2}|UXW_{1}$ can be applied. However, it is not possible to introduce $Y_{1}$ , which requires conditioning on $W_{2}$ in $Z⊥Y_{1}|UXW_{1}W_{2}$ . The opposite partition $Z^{\left[2\right]}=\left\{\emptyset ,\left\{W_{2}\right\},\left\{Z\right\}\right\}$ results in terms $I(W_{2};U|W_{1},X)$ and $I(Z;U|W_{1},X,W_{2})$ . Again, no DP inequality is applicable to $I(W_{2};U|W_{1},X)$ . We see that the remaining term $I(Z;U|W_{1},X,W_{2})$ corresponds to the term used as starting point when applying Proposition 4. This shows that in this example starting from the multivariate instrumental set comes back to Z=Z , and $B_{0}=\left\{W_{1},W_{2}\right\}$ .

The key element that leads the combination of the mutual information and unique information DP inequalities to add causal inference power is the intertwined requirements in the independencies $Z⊥Y_{1}|UXW_{1}W_{2}$ and $Z⊥Y_{2}|UXW_{1}$ . In Figure 2A, conditioning on $W_{2}$ is necessary to separate *Z* from $Y_{1}$ , since $W_{2}$ is a noncollider in $Z-W_{2}-Y_{1}$ . At the same time, $W_{2}$ cannot appear in the conditioning set to separate *Z* and $Y_{2}$ , since it is a collider in $Z-W_{2}-Y_{2}$ . This means that $W_{2}$ cannot simply be marginalized to exploit jointly the two independencies $Z⊥Y_{1}|UXW_{1}W_{2}$ and $Z⊥Y_{2}|UXW_{1}$ . It needs to first appear in the conditioning set (when applying the DP inequality of conditional mutual information) and then be excluded from the conditioning set (leading to the application of the unique information DP inequality).

This analysis of Figure 2A highlights the difference with the scenario addressed in Section 3.1, in which a single DP inequality was applied. With a single DP inequality, the unique information DP inequality can only contribute to increase causal inference power when the variables that appear in the reference argument of the unique information cannot be part of a valid instrumental set. On the other hand, when combining DP inequalities, it is the intertwined structure of the independencies associated with different types of DP inequalities what requires their combination. To further highlight this point, in Appendix C we compare in more detail Figure 2A,B. For Figure 2B, the inequality of the type of Proposition 4 derived with Z=Z and $B_{0}=\left\{W_{1},W_{2}\right\}$ does not add causal inference power to the instrumental inequality derived with $Z=\{Z,W_{2}\}$ and $B_{0}=W_{1}$ that relies only on the DP inequality of conditional mutual information. The key difference is that in Figure 2A conditioning on $W_{2}$ is necessary to create the independence between *Z* and $Y_{1}$ , while in Figure 2B the independence $Z⊥Y_{1}|UXW_{1}$ also holds. This does not create the intertwined structure of $Z⊥Y_{1}|UXW_{1}W_{2}$ and $Z⊥Y_{2}|UXW_{1}$ , which require respectively the conditioning on $W_{2}$ and non conditioning on $W_{2}$ (see Appendix C for details).

In this section, we have examined how the DP inequality of conditional mutual information and of unique information can be used sequentially to introduce new observable information terms in the lower bound of an instrumental entropic inequality. Note that the chainlike instrumental entropic inequality of Proposition 3 accommodates the use of Proposition 4. Proposition 3 indicates potential partitions of Z into $Z^{\left[r\right]}=\left\{\emptyset ,Z_{1},\ldots ,Z_{r}\right\}$ , while Proposition 4 describes a procedure to derive observable information terms that can be introduced in parallel starting separately from different summands $I(Z_{j};U|B_{0},X,Z^{\left[j-1\right]})$ of the r.h.s. of Equation (14).

In the next section we will see that the sequential addition of observable information terms can be extended with a more general type of minInf DP inequalities. With Proposition 4, we have seen that the combination of the DP inequality of conditional mutual information and unique information allows sequentially including and then removing from the conditioning set variables that are required to create an independence between Z and $Y_{1}$ , but that preclude from creating an independence between Z and $Y_{2}$ . This is achieved because, while the DP inequality of conditional mutual information operates in the original joint distribution $P(Z,U,B_{0},X,Y)$ , the DP inequality of unique information operates within the family of distributions that only preserve $P(Z,U,B_{1},X,Y_{1}\backslash \bar{Y},Y_{2})$ and $P(Z,B_{0},X,Y_{1})$ . It is the exclusion of $B_{2}=B_{0}\backslash B_{1}$ and of $\bar{Y}$ from $P(Z,U,B_{1},X,Y_{1}\backslash \bar{Y},Y_{2})$ what allows exploiting an independence with only $B_{1}$ in the conditioning set instead of $B_{0}$ . With the same logic, further relaxations of which marginals are preserved will allow us to sequentially combine more DP inequalities.

As a last remark, so far the introduction of new types of instrumental entropic inequalities (Propositions 2 and 4) has been accompanied by the comparison to related standard intrumental entropic inequalities with multivariate instrumental sets. This was necessary to verify that the new entropic inequalities do provide additional causal inference power. The examples of Figure 1C and Figure 2A show that indeed additional causal inference power can be gained either because of the lack of validity of corresponding multivariate instrumental sets (Figure 1C), or because of the intertwinement between the conditioning sets that appear in different independencies, which requires the application of the unique information DP inequality (Figure 2A). In the next sections, we will not proceed in the same way, and instead we will exclusively focus in developing instrumental entropic inequalities that add more minInf information terms in the lower bound. The verification that this addition can further increase causal inference power follows from the same logic of these previous examples. Numerical examples will be provided in Appendix H to illustrate that the addition of more minInf terms together with unique information terms increases causal inference power. It is out of the scope of this work to provide a full taxonomy of when instrumental inequalities that exploit certain types of DP inequalities are subsumed by instrumental inequalities that only exploit a subset of those types of DP inequalities. Only in Appendix J, we derive a hierarchy between specific types of instrumental entropic inequalities with related instrumental sets.

### 3.4. Recursive Use of Data Processing Inequalities to Add Observable minInf Information Terms as Lower Bounds of Information Terms with Hidden Variables

We have identified the DP inequality of unique information as the key property that allows increasing causal inference power using unique information terms. This raises the question of whether analogous DP inequalities exist for other minInf information terms defined with other sets of constraints on the preserved marginals and if so, how to recursively use these DP inequalities to insert additional observable information terms into entropic inequalities. We now show that indeed there is such a DP inequality for a more general form of minInf information terms.

This section contains our core results of how to exploit minInf DP inequalities. We have used the instrumental entropic inequality to vertebrate our presentation, but in Section 3.6 we will describe a wider framework for the finding of new entropic inequalities and in Section 3.7 we will provide further examples of the applicability of the tools here developed. To help differentiate between general results and results specific of the instrumental inequality scenario, we continue to use a different notation of variables specific for the instrumental scenario, separate from the notation used for general results. We present a general DP inequality for minInf terms using the same notation of the DP inequalities of mutual information (Lemma 1) and unique information (Lemma 3). We then show how to iteratively combine minInf DP inequalities to add new observable terms into entropic inequalities. In Section 3.5, we will show how to apply these tools concretely to the instrumental scenario.

**Proposition 5 ** 
(Data processing inequality in predictor variables of minInf information terms preserving sets of marginals). *Let $\bar{Z}$ , D , $D^{\prime }$ , E , and $E_{2}$ be five nonoverlapping sets of variables. Consider a probability distribution $P(\bar{Z},D,D^{\prime },E,E_{2})$ and the family of distributions $\Delta P_{DD^{\prime }}$ that share the set of marginals $P(\bar{Z},D,D^{\prime },O_{1})$ and $P(\bar{Z},O_{i})$ for i=2,…,m , where $O^{\left[m\right]}=\left\{O_{0},O_{1},\ldots ,O_{m}\right\}$ is a collection of subsets $O_{i}\subseteq \left\{E,E_{2}\right\}$ and $O_{0}=\emptyset$ . If the distribution $P(\bar{Z},D,D^{\prime },E,E_{2})$ is such that $\bar{Z}{⊥}_{P}D^{\prime }|DO_{1}$ , then*

(19) $$\min_{Q\in \Delta P_{DD^{\prime }}}I_{Q}\left(\bar{Z};D,D^{\prime },E_{2}|E\right)=\min_{Q\in \Delta P_{D}}I_{Q}\left(\bar{Z};D,E_{2}|E\right)\geq \min_{Q\in \Delta P_{D^{\prime }}}I_{Q}\left(\bar{Z};D^{\prime },E_{2}|E\right),$$

*where $\Delta P_{D}$ is the family of distributions that preserve $P(\bar{Z},D,O_{1})$ and $P(\bar{Z},O_{i})$ for i=2,…,m , and $\Delta P_{D^{\prime }}$ is the family of distributions that preserve $P(\bar{Z},D^{\prime },O_{1})$ and $P(\bar{Z},O_{i})$ for i=2,…,m .*

**Proof. **  Given the chain rule of mutual information

(20) $$\begin{array}{cc} \min_{Q\in \Delta P_{DD^{\prime }}}I_{Q}\left(\bar{Z};D,D^{\prime },E_{2}|E\right)= & \min_{Q\in \Delta P_{DD^{\prime }}}\left[I_{Q}\left(\bar{Z};E_{2}|E\right)\right.+\\ \; & \;\left.I_{Q}\left(\bar{Z};D|E,E_{2}\right)+I_{Q}\left(\bar{Z};D^{\prime }|E,E_{2},D\right)\right], \end{array}$$

and

(21) $$\begin{array}{c} \min_{Q\in \Delta P_{D}}I_{Q}\left(\bar{Z};D,E_{2}|E\right)=\min_{Q\in \Delta P_{D}}\left[I_{Q}\left(\bar{Z};E_{2}|E\right)+I_{Q}\left(\bar{Z};D|E,E_{2}\right)\right]. \end{array}$$

Now consider a distribution that minimizes Equation (21), namely

(22) $$\begin{array}{c} Q^{*}\left(\bar{Z},D,E,E_{2}\right)\equiv \arg\min_{Q\in \Delta P_{D}}I_{Q}\left(\bar{Z};D,E_{2}|E\right). \end{array}$$

Construct $\bar{Q}\left(\bar{Z},D,D^{\prime },E,E_{2}\right)\equiv P\left(D^{\prime }|D,O_{1}\right)Q^{*}\left(\bar{Z},D,E,E_{2}\right)$ . Given that $Q^{*}\in \Delta P_{D}$ , it preserves the marginals $P(\bar{Z},O_{i})$ for i=2,…,m and $P(\bar{Z},D,O_{1})$ . Furthermore, $\bar{Q}$ by construction preserves $\bar{Z}{⊥}_{P}D^{\prime }|DO_{1}$ , which means that it preserves $P(\bar{Z},D,D^{\prime },O_{1})$ , and hence $\bar{Q}\in \Delta P_{DD^{\prime }}$ , since all other constraints to preserve marginals are the same in $\Delta P_{DD^{\prime }}$ and $\Delta P_{D}$ . Since the first two terms in the sum of Equation (20) do not depend on $D^{\prime }$ their minimization is the same in $\Delta P_{D}$ or $\Delta P_{DD^{\prime }}$ and $Q^{*}\left(\bar{Z},D,E,E_{2}\right)$ minimizes their sum, which is equal to the one in Equation (21). By construction of $\bar{Q}$ , the independence $\left\{\bar{Z},E,E_{2}\right\}\backslash O_{1}{⊥}_{\bar{Q}}D^{\prime }|DO_{1}$ holds and hence, using the weak union axiom of semi-graphoids for mutual information [25,43], also the independence $\bar{Z}{⊥}_{\bar{Q}}D^{\prime }|DEE_{2}$ holds. This means that the last term in the sum of Equation (20) is zero for $\bar{Q}$ . Therefore, $\bar{Q}$ minimizes the r.h.s. of Equation (20), which is equal to the r.h.s. of Equation (21), so that $\min_{Q\in \Delta P_{DD^{\prime }}}I_{Q}\left(\bar{Z};D,D^{\prime },E_{2}|E\right)$ is equal to $\min_{Q\in \Delta P_{D}}I_{Q}\left(\bar{Z};D,E_{2}|E\right)$ , with the minima reached by $\bar{Q}\left(\bar{Z},D,D^{\prime },E,E_{2}\right)$ and $Q^{*}\left(\bar{Z},D,E,E_{2}\right)$ , respectively. Furthermore, monotonicity of mutual information guarantees that information can only decrease when removing variable D from $\left\{D,D^{\prime }\right\}$ , namely $I_{\bar{Q}}\left(\bar{Z};D^{\prime },E_{2}|E\right)$ is smaller than or equal to $I_{\bar{Q}}\left(\bar{Z};D,D^{\prime },E_{2}|E\right)$ . Finally, $\min_{Q\in \Delta P_{D^{\prime }}}I_{Q}\left(\bar{Z};D^{\prime },E_{2}|E\right)$ by definition is smaller than or equal to $I_{\bar{Q}}\left(\bar{Z};D^{\prime },E_{2}|E\right)$ . □

Proposition 5 encompasses Lemmas 1 and 3 as subcases. The DP inequality of conditional mutual information is subsumed with m=1 , $E_{2}=\emptyset$ , and $O_{1}=E$ , such that $P(\bar{Z},D,D^{\prime },O_{1})$ corresponds to the joint original distribution. The DP inequality of unique information is subsumed with m=2 , $E_{2}=\emptyset$ , $O_{2}=E$ , $O_{1}\subset O_{2}$ . This results in a unique information $I(\bar{Z};D,D^{\prime }\backslash \backslash D_{2}|O_{1})$ , with $D_{2}=\left\{O_{2}\backslash O_{1}\right\}$ , as in Lemma 3.

Given this DP inequality for minInf information terms, we now describe how it can be used to iteratively add new observable information terms in a lower bound of a minInf information term containing as predictor hidden variables. We start with an example to gain some intuition of the procedure. For this purpose, we recap how the DP inequalities of mutual information and unique information are sequentially combined in Proposition 4, concretely in the example of Figure 2A. We then point out how a similar procedure can be used to sequentially combine more minInf DP inequalities, using Figure 2C as an example.

The first row of Table 1 summarizes how the relaxation of preserved marginals allows combining the DP inequalities of mutual information and unique information in Figure 2A to sequentially insert $Y_{1}$ and $Y_{2}$ . The key aspect of this relaxation is that only the variables involved in the independence $Z⊥Y_{2}|UXW_{1}$ are preserved in the marginal $P(Z,U,W_{1},X)$ that includes the hidden variable. This allows applying the unique information DP inequality to insert $Y_{2}$ , while Z⊥Y2|UXW1W2 does not allow applying the mutual information DP inequality. The second row of Table 1 summarizes the analogous procedure applied to Figure 2C to combine the DP inequalities of mutual information and unique information to sequentially insert $Y_{1}$ and $Y_{2}$ . A more detailed examination of the corresponding instrumental entropic inequality that holds for Figure 2C will be examined in Section 3.5. Here, our interest is to motivate that the same procedure of relaxation of the preserved marginals allows applying a third minInf DP inequality to insert $Y_{3}$ .

This is shown in the third row of Table 1. The preserved marginals $\{P\left(Z,U,Y_{2},W_{3},X\right),$ $P(Z,Y_{1},W_{2},W_{3},X)\}$ in the third column of (ii), which allow applying the DP inequality of unique information, are the departing set in the first column of (iii). Then a new relaxation of the marginals divides $P(Z,U,Y_{2},W_{3},X)$ into $\left\{P\left(Z,U,Y_{2},X\right),P\left(Z,Y_{2},W_{3},X\right)\right\}$ . This allows preserving in $P(Z,U,Y_{2},X)$ only the variables involved in the independence $Z⊥Y_{3}|UXY_{2}$ , while Z⊥Y3|UXW3Y2 . The other marginal $P(Z,Y_{1},W_{2},W_{3},X)$ is left unchanged during the relaxation. We then recognize in the structure of the marginals preserved after the iterative application of the relaxations the pattern of constraints of the families of distributions considered in Proposition 5, namely the fact that the hidden variables and conditioning variables involved in the subsequent conditional independence to be exploited are the only ones included together with $\bar{Z}$ in the marginal distribution that plays the role of $P(\bar{Z},D,O_{1})$ . Accordingly, the minInf DP inequality of Proposition 5 is used to insert $Y_{3}$ .

We now formalize how DP inequalities of minInf terms that are determined by sequential relaxations of the preserved marginals can be combined:

**Theorem 1 ** 
(Iterative addition of observable minInf information terms to lower bounds of unobservable minInf information terms). *Consider nonoverlapping sets of variables $\bar{Z}$ , E , and $\bar{U}$ with all observable except $\bar{U}$ hidden variables. For k≥1 , consider a nonempty collection of observable nonoverlapping sets of variables $A^{\left[k\right]}=\left\{A_{0},A_{1},\ldots ,A_{k}\right\}$ , with $A_{0}=\emptyset$ . Consider a collection ${\bar{Z}}^{\left[k\right]}=\left\{{\bar{Z}}_{0},{\bar{Z}}_{1},\ldots ,{\bar{Z}}_{k}\right\}$ and a collection ${\overset{ˇ}{Z}}^{\left[k\right]}=\left\{{\overset{ˇ}{Z}}_{0},{\overset{ˇ}{Z}}_{1},\ldots ,{\overset{ˇ}{Z}}_{k}\right\}$ such that ${\bar{Z}}_{0}=\bar{Z}$ , ${\overset{ˇ}{Z}}_{0}=\emptyset$ , and ${\overset{ˇ}{Z}}_{j}\subset {\bar{Z}}_{j-1}$ , ${\bar{Z}}_{j}\subseteq {\bar{Z}}_{j-1}\backslash {\overset{ˇ}{Z}}_{j}$ , for j=1,…,k . Consider a collection ${\bar{U}}^{\left[k\right]}=\left\{{\bar{U}}_{0},{\bar{U}}_{1},\ldots ,{\bar{U}}_{k}\right\}$ such that ${\bar{U}}_{0}={\bar{U}}_{1}=\bar{U}$ and ${\bar{U}}_{j}\subseteq {\bar{U}}_{j-1}$ , for j=1,…,k . Consider the collections of sets of variables ${\bar{B}}^{\left[k\right]}=\left\{{\bar{B}}_{0},{\bar{B}}_{1},\ldots ,{\bar{B}}_{k}\right\}$ and $C^{\left[k\right]}=\left\{C_{0},C_{1},\ldots ,C_{k}\right\}$ , with ${\bar{B}}_{0}=E$ , $C_{0}=\emptyset$ , and iteratively constructed such that ${\bar{B}}_{1}^{\prime }\subseteq \left\{A_{0},{\bar{B}}_{0}\right\}=E$ , ${\bar{B}}_{1}=\left\{{\bar{B}}_{1}^{\prime },{\overset{ˇ}{Z}}_{1}\right\}$ , $C_{1}=\left\{A_{0},{\bar{B}}_{0},{\overset{ˇ}{Z}}_{1}\right\}=\left\{E,{\overset{ˇ}{Z}}_{1}\right\}$ , and for j>1 , ${\bar{B}}_{j}^{\prime }\subseteq \left\{A_{j-1},{\bar{B}}_{j-1}\right\}$ , ${\bar{B}}_{j}=\left\{{\bar{B}}_{j}^{\prime },{\overset{ˇ}{Z}}_{j}\right\}$ , and $C_{j}=\left\{A_{j-1},{\bar{B}}_{j-1},{\overset{ˇ}{Z}}_{j}\right\}$ , so that ${\bar{B}}_{j}\subseteq C_{j}$ , for j=1,…,k . Consider a joint distribution $P(\bar{Z},\bar{U},A^{\left[k\right]},E)$ . Consider the family of distributions $\Delta P_{k-1}$ preserving $P({\bar{Z}}_{k-1},{\bar{U}}_{k-1},A_{k-1},{\bar{B}}_{k-1})$ and $P({\bar{Z}}_{j},C_{j})$ , for j=1,…,k−1 . Consider the family of distributions $\Delta P_{k}$ preserving $P({\bar{Z}}_{k},{\bar{U}}_{k},A_{k},{\bar{B}}_{k})$ and $P({\bar{Z}}_{j},C_{j})$ , for j=1,…,k . If ${\bar{Z}}_{k}{⊥}_{P}A_{k}|{\bar{U}}_{k}{\bar{B}}_{k}$ , then*

(23) $$\begin{array}{cc} \min_{Q\in \Delta P_{k-1}}I_{Q}\left({\bar{Z}}_{k-1};{\bar{U}}_{k-1}|E,A^{\left[k-1\right]},{\overset{ˇ}{Z}}^{\left[k-1\right]}\right)\geq & \min_{Q\in \Delta P_{k}}I_{Q}\left({\bar{Z}}_{k};A_{k}|E,A^{\left[k-1\right]},{\overset{ˇ}{Z}}^{\left[k\right]}\right)+\\ & \min_{Q\in \Delta P_{k}}I_{Q}\left({\bar{Z}}_{k};{\bar{U}}_{k}|E,A^{\left[k\right]},{\overset{ˇ}{Z}}^{\left[k\right]}\right). \end{array}$$

**Proof. **  The proof is provided in Appendix E. □

Theorem 1 provides a way to iteratively add additional observable terms at the lower bound of nonobservable information terms with hidden variables. A full understanding of how it proceeds can be gained with its proof. In the rest of this section, we highlight its main properties, describing the transition from the exploitation of independence ${\bar{Z}}_{k-1}{⊥}_{P}A_{k-1}|{\bar{U}}_{k-1}{\bar{B}}_{k-1}$ in iteration k−1 to the exploitation of ${\bar{Z}}_{k}{⊥}_{P}A_{k}|{\bar{U}}_{k}{\bar{B}}_{k}$ in iteration *k*. We start with the simplest scenario, in which ${\bar{Z}}_{j}=\bar{Z}$ , ${\bar{U}}_{j}=\bar{U}$ , and ${\overset{ˇ}{Z}}_{j}=\emptyset$ , for j=1,…,k . This case already allows appreciating the core of the recursiveness. It corresponds to the scenario in which the DP inequalities being used all have the same target variable $\bar{Z}$ and rely on the same set of hidden variables $\bar{U}$ . Given that ${\overset{ˇ}{Z}}_{j}=\emptyset$ for j=1,…,k , the iterative construction of ${\bar{B}}^{\left[k\right]}$ and $C^{\left[k\right]}$ can be simplified to ${\bar{B}}_{1}\subseteq \left\{A_{0},{\bar{B}}_{0}\right\}=E$ , $C_{1}=\left\{A_{0},{\bar{B}}_{0}\right\}=E$ , and for j>1 , ${\bar{B}}_{j}\subset \left\{A_{j-1},{\bar{B}}_{j-1}\right\}$ and $C_{j}=\left\{A_{j-1},{\bar{B}}_{j-1}\right\}$ . The auxiliary variables ${\bar{B}}_{j}^{\prime }$ are not needed when ${\overset{ˇ}{Z}}_{j}=\emptyset$ for j=1,…,k because their role is only to add ${\overset{ˇ}{Z}}_{j}$ in ${\bar{B}}_{j}=\left\{{\bar{B}}_{j}^{\prime },{\overset{ˇ}{Z}}_{j}\right\}$ . Furthermore, for j>1 , we have ${\bar{B}}_{j}\subset \left\{A_{j-1},{\bar{B}}_{j-1}\right\}$ instead of ${\bar{B}}_{j}\subseteq \left\{A_{j-1},{\bar{B}}_{j-1}\right\}$ because an equality ${\bar{B}}_{j}=\left\{A_{j-1},{\bar{B}}_{j-1}\right\}$ leads to applying a DP inequality in step j−1 with the independence $\bar{Z}{⊥}_{P}A_{j-1}|\bar{U}{\bar{B}}_{j-1}$ and in step *j* with $\bar{Z}{⊥}_{P}A_{j}|\bar{U}A_{j-1}{\bar{B}}_{j-1}$ . These two steps can then be merged in a new step j−1 that jointly adds $\left\{A_{j-1},A_{j}\right\}$ given $\bar{Z}{⊥}_{P}A_{j-1}A_{j}|\bar{U}{\bar{B}}_{j-1}$ , based on the *contraction* axiom of semi-graphoids [25,43].

For this simplest scenario, we now highlight the core of the recursiveness. The independencies of step k−1 and *k* are $\bar{Z}{⊥}_{P}A_{k-1}|\bar{U}{\bar{B}}_{k-1}$ and $\bar{Z}{⊥}_{P}A_{k}|\bar{U}{\bar{B}}_{k}$ , respectively. The family $\Delta P_{k-1}$ preserves $P(\bar{Z},\bar{U},A_{k-1},{\bar{B}}_{k-1})$ and $P(\bar{Z},C_{j})$ for j=1,…,k−1 , and given that $C_{k}=\left\{A_{k-1},{\bar{B}}_{k-1}\right\}$ , it hence preserves $P\left(\bar{Z},\bar{U},A_{k-1},{\bar{B}}_{k-1}\right)=P\left(\bar{Z},\bar{U},C_{k}\right)$ . In iteration k−1 , variables $A_{k-1}$ are introduced using Proposition 5 with $\bar{Z}{⊥}_{P}A_{k-1}|\bar{U}{\bar{B}}_{k-1}$ . Here the variables $\left\{Z,D,D^{\prime },O_{1},E,E_{2}\right\}$ of Proposition 5 are assigned as $\left\{\bar{Z},\bar{U},A_{k-1},{\bar{B}}_{k-1},\left\{E,A^{\left[k-2\right]}\right\},\emptyset \right\}$ . The family $\Delta P_{k-1}$ plays the role of $\Delta P_{DD^{\prime }}$ in Equation (19). When moving from $\Delta P_{k-1}$ to $\Delta P_{k}$ , the preservation of $P\left(\bar{Z},\bar{U},A_{k-1},{\bar{B}}_{k-1}\right)=P\left(\bar{Z},\bar{U},C_{k}\right)$ is loosen to the preservation of two of its marginals, namely $P(\bar{Z},\bar{U},{\bar{B}}_{k})$ and $P(\bar{Z},C_{k})$ . The first is a marginal because ${\bar{B}}_{k}\subseteq C_{k}$ . The second is a marginal because $\bar{U}$ is removed. Now $\bar{U}$ only appears in $P(\bar{Z},\bar{U},{\bar{B}}_{k})$ . The variables $A_{k}$ are introduced analogously to $A_{k-1}$ , using Proposition 5 now with $\bar{Z}{⊥}_{P}A_{k}|\bar{U}{\bar{B}}_{k}$ . Here $\left\{Z,D,D^{\prime },O_{1},E,E_{2}\right\}$ are assigned as $\left\{\bar{Z},\bar{U},A_{k},{\bar{B}}_{k},\left\{E,A^{\left[k-1\right]}\right\},\emptyset \right\}$ . The family $\Delta P_{k}$ plays the role of $\Delta P_{DD^{\prime }}$ in Equation (19). The second term at the r.h.s. of Equation (23) has the same form as the one at the l.h.s., replacing k−1 by *k*. Comparing the two independencies used in steps k−1 and *k*, ${\bar{B}}_{k}$ used in $\bar{Z}{⊥}_{P}A_{k}|\bar{U}{\bar{B}}_{k}$ is a subset of ${\bar{B}}_{k-1}$ used in $\bar{Z}{⊥}_{P}A_{k-1}|\bar{U}{\bar{B}}_{k-1}$ , except for the possible addition of variables from $A_{k-1}$ . This follows the same pattern already seen in Figure 2A with $Z⊥Y_{1}|UXW_{1}W_{2}$ and $Z⊥Y_{2}|UXW_{1}$ , where $A_{1}=\left\{Y_{1}\right\}$ , $A_{2}=\left\{Y_{2}\right\}$ , ${\bar{B}}_{1}=\left\{X,W_{1},W_{2}\right\}$ , and ${\bar{B}}_{2}=\left\{X,W_{1}\right\}$ , or in Figure 2B with $Z⊥Y_{1}|UXW_{1}W_{2}$ and $Z⊥Y_{2}|UXW_{1}Y_{1}$ , where $A_{1}$ , $A_{2}$ , and ${\bar{B}}_{1}$ are the same, and ${\bar{B}}_{2}=\left\{X,W_{1},Y_{1}\right\}$ .

**Figure A2:**
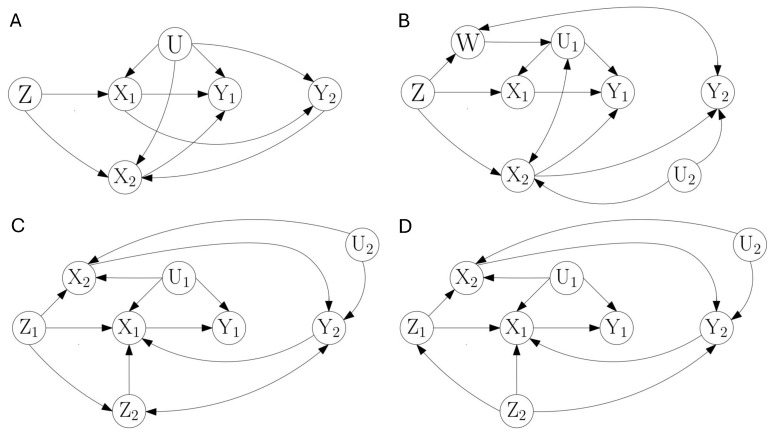
Examples for the application of Theorem 1 in the derivation of instrumental entropic inequalities of Proposition 6. All variables are observable except U hidden. These examples are described in Appendix F. (**A**) Causal structure related to Equation (A11). (**B**) Causal structure related to Equation (A12). (**C**) Causal structure related to Equation (A13). (**D**) Causal structure related to Equation (A14).

We now provide an overview of the rest of scenarios. Concrete examples are described in Section 3.5 and in Appendix F. These other scenarios comprise cases in which ${\bar{Z}}_{j}$ or ${\bar{U}}_{j}$ are not constant for j=1,…,k . The cases with non constant ${\bar{U}}_{j}$ , given that ${\bar{U}}_{j}\subseteq {\bar{U}}_{j-1}$ , correspond to cases in which some of the hidden variables $\bar{U}$ are marginalized before applying subsequent DP inequalities. This happens when in step *j* the variables ${\bar{U}}_{j-1}\backslash {\bar{U}}_{j}$ are colliders or descendants of colliders in paths that lead to ${\bar{Z}}_{j}PA_{j}|{\bar{U}}_{j-1}{\bar{B}}_{j}$ as opposed to ${\bar{Z}}_{j}{⊥}_{P}A_{j}|{\bar{U}}_{j}{\bar{B}}_{j}$ . An example will be shown in Figure A2B.

Similarly, if ${\overset{ˇ}{Z}}_{j}=\emptyset$ for j=1,…,k , the cases with non constant ${\bar{Z}}_{j}$ for some *j* correspond to cases in which some target variables are marginalized to apply subsequent DP inequalities. This is because the relations ${\overset{ˇ}{Z}}_{j}\subset {\bar{Z}}_{j-1}$ , ${\bar{Z}}_{j}\subseteq {\bar{Z}}_{j-1}\backslash {\overset{ˇ}{Z}}_{j}$ , for j=1,…,k simplify to ${\bar{Z}}_{j}\subseteq {\bar{Z}}_{j-1}$ when ${\overset{ˇ}{Z}}_{j}=\emptyset$ for j=1,…,k . This happens in step *j* when ${\bar{Z}}_{j-1}PA_{j}|\bar{U}{\bar{B}}_{j}$ , as opposed to ${\bar{Z}}_{j}{⊥}_{P}A_{j}|\bar{U}{\bar{B}}_{j}$ , because the variables ${\bar{Z}}_{j-1}\backslash {\bar{Z}}_{j}$ have active paths reaching $A_{j}$ . An example will be shown in Figure A2C.

Finally, the case in which ${\overset{ˇ}{Z}}_{j}\neq \emptyset$ for some *j* covers cases in which conditioning on ${\overset{ˇ}{Z}}_{j}\subset {\bar{Z}}_{j-1}$ is necessary to create the independence ${\bar{Z}}_{j}{⊥}_{P}A_{j}|\bar{U}{\bar{B}}_{j}$ , with ${\overset{ˇ}{Z}}_{j}\subseteq {\bar{B}}_{j}$ . Accordingly, in step *j* the variables ${\overset{ˇ}{Z}}_{j}$ are moved from target variables to conditioning variables using a chain rule of the information terms. An example will be shown in Figure A2D. Furthermore, the marginalization of some variables in $\bar{U}$ , the marginalization of some variables in $\bar{Z}$ , and the conditioning on some ${\overset{ˇ}{Z}}_{j}$ can co-occur in the same step *j*, leading to the final general formulation of Theorem 1. The commonality to all scenarios is that in Equation (23), while the term at the l.h.s. is not observable, the first term at r.h.s. does not depend on any hidden variable and hence leads to an observable term by relaxing the preservation of $P({\bar{Z}}_{k},{\bar{U}}_{k},A_{k},{\bar{B}}_{k})$ to $P({\bar{Z}}_{k},A_{k},{\bar{B}}_{k})$ . Furthermore, the second term of the r.h.s. has the same form as the term at the l.h.s., which means that Theorem 1 can be applied recursively.

### 3.5. Instrumental Entropic Inequalities with Sums of minInf Information Terms

We now show how to use Theorem 1 to create testable entropic inequalities from the nontestable inequalities of Proposition 3. Reexamining the terms $I(Z_{j};U|B_{0},X,Z^{\left[j-1\right]})$ , j=1,…,r , that appear in the r.h.s. of Equation (14), we see that each of these terms can be the starting point to iterate the addition of observable information terms using Theorem 1. The application of Theorem 1 to a nonempty subset of a partition $Z^{\left[r\right]}=\left\{\emptyset ,Z_{1},\ldots ,Z_{r}\right\}$ converts a nontestable instrumental entropic inequality from Proposition 3 into testable.

**Proposition 6 ** 
(Testable instrumental entropic inequalities from the iterative application of data processing inequalities to minInf information terms). *Consider nonoverlapping sets of variables Z , X , $B_{0}$ , and U , all observable except U hidden variables. Consider that the joint distribution of these variables is generated from a causal structure that creates the independence $Z⊥U|B_{0}$ , which leads to a nontestable entropic inequality of the form $H\left(X|B_{0}\right)\geq I\left(Z;X|B_{0}\right)+I\left(Z;U|B_{0},X\right)$ . Consider an exclusive partition in r parts of the instrumental set Z given by $Z^{\left[r\right]}=\left\{\emptyset ,Z_{1},\ldots ,Z_{r}\right\}$ , such that $I(Z;U|B_{0},X)$ is separated in the sum of r nonestimable information terms $I(Z_{k};U|B_{0},X,Z^{\left[k-1\right]})$ , k=1,…,r . Select $E_{k}=\left\{B_{0},X,Z^{\left[k-1\right]}\right\}$ . Consider a nonoverlapping subset ${\tilde{Z}}^{\left[q\right]}=\left\{\emptyset ,{\tilde{Z}}_{1},\ldots ,{\tilde{Z}}_{q}\right\}\subseteq Z^{\left[r\right]}$ , 0<q≤r , such that each ${\tilde{Z}}_{l}\in {\tilde{Z}}^{\left[q\right]}$ corresponds to a different $Z_{i}\in Z^{\left[r\right]}$ . Consider that for each $Z_{i}\in {\tilde{Z}}^{\left[q\right]}$ it is possible to iteratively apply Theorem 1 with an initial assignment of its inputs $\left\{\bar{Z},E,\bar{U}\right\}$ as $\left\{Z_{i},E_{i},U_{i}\right\}$ , with $U_{i}\subseteq U$ . Accordingly, for each $Z_{i}\in {\tilde{Z}}^{\left[q\right]}$ , it is possible to construct collections $A_{i}^{\left[n_{i}\right]}$ , ${\bar{Z}}_{i}^{\left[n_{i}\right]}$ , ${\overset{ˇ}{Z}}_{i}^{\left[n_{i}\right]}$ , ${\bar{U}}_{i}^{\left[n_{i}\right]}$ , ${\bar{B}}_{i}^{\left[n_{i}\right]}$ , and $C_{i}^{\left[n_{i}\right]}$ , with $n_{i}>0$ , which are associated with sets of independencies ${\bar{Z}}_{ij}{⊥}_{P}A_{ij}|{\bar{U}}_{ij}{\bar{B}}_{ij}$ , for $j=1,\ldots ,n_{i}$ , which are imposed by the causal structure. This leads to the testable instrumental entropic inequality*

(24) $$\begin{array}{c} H\left(X|B_{0}\right)\geq I\left(Z;X|B_{0}\right)+\sum_{Z_{i}\in {\tilde{Z}}^{\left[q\right]}}\sum_{j=1}^{n_{i}}\min_{Q\in \Delta P_{ij}}I_{Q}\left({\bar{Z}}_{ij};A_{ij}|E_{i},A_{i}^{\left[j-1\right]},{\overset{ˇ}{Z}}_{i}^{\left[j\right]}\right), \end{array}$$

*where each family of distributions $\Delta P_{ij}$ preserves $P({\bar{Z}}_{ij},A_{ij},{\bar{B}}_{ij})$ and $P({\bar{Z}}_{ik},C_{ik})$ , for k=1,…,j .*

**Proof. **  Proposition 6 follows directly from the iterative application of Theorem 1 to a subset of the nonobservable information terms in the sum of Equation (14). In each case, the theorem is applied starting from a different set of variables $\left\{\bar{Z},E,\bar{U}\right\}$ , namely $\left\{Z_{i},E_{i},U_{i}\right\}$ , where $Z_{i}\in Z^{\left[r\right]}$ corresponds to some ${\tilde{Z}}_{l}\in {\tilde{Z}}^{\left[q\right]}$ . The variables in U not included in $U_{i}$ are marginalized. Theorem 1 describes the properties that need to fulfill the collections $A_{i}^{\left[n_{i}\right]}$ , ${\bar{Z}}_{i}^{\left[n_{i}\right]}$ , ${\overset{ˇ}{Z}}_{i}^{\left[n_{i}\right]}$ , ${\bar{U}}_{i}^{\left[n_{i}\right]}$ , ${\bar{B}}_{i}^{\left[n_{i}\right]}$ , and $C_{i}^{\left[n_{i}\right]}$ . The requirements q>0 and $n_{i}>0$ ensure that at least one observable information term is added in the lower bound, such that a nontrivial entropic inequality is testable. The form of the resulting testable inequality is determined by which independencies are imposed by the causal structure of interest, that is, which sets of independencies ${\bar{Z}}_{ij}{⊥}_{P}A_{ij}|{\bar{U}}_{ij}{\bar{B}}_{ij}$ , for i=1,…,q , $j=1,\ldots ,n_{i}$ are combined to apply DP inequalities that add estimable information terms at the lower bound. □

The inequality of Proposition 6 encompasses the ones of Proposition 1, 2, and 4. It may be asked why the term $I(Z;X|B_{0})$ is always separated before starting to apply DP inequalities. In fact, in the case of Proposition 1 where the standard DP inequality is applied, this is not a differentiating factor, since the r.h.s. of Equation (1) is equal to $I(Z;X,Y|B_{0})$ . However, when a DP inequality is applied in combination with relaxations of the constraints on the marginals to be preserved, this changes. As elaborated after the proof of Theorem 1 in Appendix E, in order to obtain a lower bound as tight as possible the constraints on the marginals should always be as strong as possible, while loose enough to allow the application of the subsequent DP inequalities. Given that the term $I(Z;X|B_{0})$ is observable, a minimization after a relaxation of the constraints that would not preserve $P(Z,X,B_{0})$ results in an equal or smaller lower bound. This means that, to obtain the highest lower bound, DP inequalities should be applied starting with $I(Z;U|B_{0},X)$ , after the separation of $I(Z;X|B_{0})$ . Accordingly, we will further illustrate in Figure A2A that X and $B_{0}$ play the same role in the derivation of an estimable lower bound.

We now examine in detail the application of Proposition 6 to the example of Figure 2C. The causal structure of Figure 2C is analogous to the one of Figure 2A, with some differences: It contains an additional predictor $Y_{3}$ and a new conditioning variable $W_{3}$ . The conditioning variable $W_{1}$ has been removed for simplicity of the figure, but could be left as in Figure 2A with no qualitative effect in our reasoning. For simplicity of the explanation, we now focus on the construction of an instrumental entropic inequality with instrumental set Z={Z} and conditioning set $B_{0}=\left\{W_{2},W_{3}\right\}$ . We do so because this suffices to illustrate the iterative application of minInf DP inequalities with Proposition 6. See Appendix I for a more detailed analysis of this example. With Z={Z} and $B_{0}=\left\{W_{2},W_{3}\right\}$ , we apply Proposition 6 with $A^{\left[n\right]}=\left\{\emptyset ,\left\{Y_{1}\right\},\left\{Y_{2}\right\},\left\{Y_{3}\right\}\right\}$ , using the independencies $Z⊥Y_{1}|UXW_{2}W_{3}$ , $Z⊥Y_{2}|UXW_{3}$ , and $Z⊥Y_{3}|UXY_{2}$ . The derived entropic inequality is

(25) $$\begin{array}{cc} H(X|W_{2},W_{3})\geq & I\left(Z;X,Y_{1}|W_{2},W_{3}\right)+\min_{Q\in \Delta P_{2}}I_{Q}\left(Z;Y_{2}|W_{2},W_{3},X,Y_{1}\right)+\\ & \min_{Q\in \Delta P_{3}}I_{Q}\left(Z;Y_{3}|W_{2},W_{3},X,Y_{1},Y_{2}\right), \end{array}$$

where $\Delta P_{2}$ preserves the marginals $\left\{P\left(Z,W_{3},X,Y_{2}\right),P\left(Z,W_{2},W_{3},X,Y_{1}\right)\right\}$ and $\Delta P_{3}$ preserves the marginals $\left\{P\left(Z,X,Y_{2},Y_{3}\right),P\left(Z,W_{3},X,Y_{2}\right),P\left(Z,W_{2},W_{3},X,Y_{1}\right)\right\}$ . The second term in the r.h.s. is the minInf term that corresponds to $I(Z;Y_{2}\backslash \backslash \left\{W_{2},Y_{1}\right\}|W_{3},X)$ . Variable $Y_{1}$ is inserted thanks to $Z⊥Y_{1}|UXW_{2}W_{3}$ , using the standard DP inequality of Lemma 1. Variable $Y_{2}$ is inserted thanks to $Z⊥Y_{2}|UXW_{3}$ , using the DP inequality of unique information of Lemma 3. Finally, variable $Y_{3}$ is inserted thanks to $Z⊥Y_{3}|UXY_{2}$ , using a minInf DP inequality of the form of Proposition 5.

Our objective with this example was to illustrate the iterative insertion of estimable information terms in the lower bound. As mentioned above, an extended analysis of instrumental entropic inequalities for the causal structure of Figure 2C is presented in Appendix I. This extended presentation will cover alternative entropic inequalities that are derived with the multivariate instrumental set $Z=\{Z,W_{2},W_{3}\}$ , when using different partitions to create chainlike instrumental entropic inequalities of the form of Equation (14). Concretely, the inequality of Equation (25) is subsumed by the one of Equation (A17e). Appendix I provides further evidence of the increase in causal inference power thanks to the addition of minInf terms, since they allow obtaining tighter lower bounds thanks to the combination of conditional independencies that cannot be jointly used in a standard instrumental inequality.

This completes our extension of instrumental entropic inequalities, from the standard form reviewed in Proposition 1, to the minInf instrumental entropic inequalities of Proposition 6. We have used the specific scenario of instrumental inequalities to vertebrate the presentation of our core contributions, namely the theoretical derivation of DP inequalities for minInf information terms (Proposition 5) and the iterative procedure to combine them (Theorem 1). In the rest of our Results, we will more broadly show how to apply these tools to derive other entropic inequalities apart from instrumental inequalities.

While our main contribution focuses on the theoretical derivation of the properties of minInf terms that render them useful for causal structure learning, in Appendix G we also discuss their estimation. As we explain, this estimation constitutes a non-convex minimization problem [44] and a general implementation is out of the scope of this work. Nonetheless, in Lemma A2 we recast minInf terms separating a convex and non-convex component of the minimization problem. We use this approach to extend the numerical examples presented in Appendix D, which show the gain in causal inference power obtained with Proposition 4. In this way, in Appendix H we also provide numerical examples in which it is only thanks to the addition of a second minInf term, like in Equation (25), that a rejection is obtained when testing the entropic inequality.

### 3.6. The Region of minInf Shannon Entropy Cones

In previous sections we have derived entropic inequalities with increased causal inference power by introducing minInf DP inequalities. We here more broadly reformulate this derivation from a geometrical perspective. In a geometrical perspective of entropy [22], entropy values associated with a set of variables $\bar{V}=\left\{{\bar{V}}_{1},{\bar{V}}_{2},\ldots ,{\bar{V}}_{n}\right\}$ are represented as a point in a $R^{2^{n}}$ space. In more detail, given a joint distribution $P(\bar{V})$ , for the set of indices [n]={1,2,…,n} associated with the variables, an entropy value is obtained for each subset of indexes S⊆[n] . This entropy value corresponds to the joint entropy $H({\bar{V}}_{S})$ of the marginal probability distribution $P({\bar{V}}_{S})$ . Given that the *power set* of subsets of [n] contains $2^{n}$ subsets, a vector constructed with all entropy values $H({\bar{V}}_{S})$ lies in a $R^{2^{n}}$ space. The region in this space containing all points obtainable from probability distributions, the *entropy cone*, forms a convex cone (Theorem 15.5 in [22]), but has an unknown explicit characterization. However, an approximation of this region is given by the *Shannon entropy cone*, which includes all points that comply with the following linear inequality constraints:

(26a) $$\begin{array}{cc} \; & H(\emptyset )=0, \end{array}$$

(26b) $$\begin{array}{cc} \; & H\left({\bar{V}}_{T}\right)\geq H\left({\bar{V}}_{S}\right)\;\;\mathrm{if}\;\;S\subseteq T, \end{array}$$

(26c) $$\begin{array}{cc} \; & I({\bar{V}}_{S};{\bar{V}}_{T}|{\bar{V}}_{S\cap T})\geq 0, \end{array}$$

where *S* and *T* are two subsets of [n] . These inequalities are known as the *basic inequalities* [22,28] and can be expressed as linear inequalities only involving entropy terms, hence introducing constraints among different components of the entropy vectors. The basic inequalities impose requirements for any well-defined probability distribution, namely the nonnegativity of entropy (Equation (26a,b)), the monotonicity of entropy (Equation (26b)), and the nonnegativity of conditional mutual information (Equation (26c)), associated with the submodularity of entropy.

These basic inequalities are constraints that apply to any entropic vector created from a well-defined probability distribution. Furthermore, if a joint probability distribution of interest is generated under additional constraints, such as the compatibility with a certain causal structure, then the set of independencies induced by the causal structure adds extra equality constraints to the basic inequalities, namely in the form of conditional mutual information terms with zero values. In the presence of hidden variables, the cancelation of conditional mutual information terms involving the hidden variables is not verifiable. However, given the set combining the basic inequalities and the causally-induced equalities, it has been shown [14,24] that causally informative entropic inequalities, such as the standard instrumental entropic inequality, are derived by marginalization of the hidden variables to obtain inequalities that only involve observable variables. This marginalization has been algorithmically implemented using Fourier-Motzkin elimination, a standard linear programming algorithm for the elimination of variables from systems of inequalities [14].

The derivation of entropic inequalities with minInf terms can be formulated as an analogous marginalization problem, but starting from the region of a *minInf Shannon entropy cone*, which generalizes the region of the Shannon entropy cone from individual distributions to families of distributions sharing sets of constraints. In more detail, consider a minInf term

(27) $$\begin{array}{c} \min_{Q\in \Delta P}I_{Q}\left({\bar{V}}_{1};{\bar{V}}_{2}|{\bar{V}}_{3}\right), \end{array}$$

where ${\bar{V}}_{i}$ , i=1,…,3 are sets of variables, without specification of which variables are observable or hidden. The family of distributions ΔP is defined as preserving a set of marginals from a joint original distribution $P(\bar{V})$ , with $\left\{{\bar{V}}_{1},{\bar{V}}_{2},{\bar{V}}_{3}\right\}\subseteq \bar{V}$ . The minimum within ΔP determines a set of distributions, at least one, that reach the minimum. Select a distribution $Q^{*}\left({\bar{V}}_{1},{\bar{V}}_{2},{\bar{V}}_{3}\right)$ among the ones reaching the minimum and consider the region that its entropic vector can occupy. This region is restricted by basic inequalities (Equation (26)) and also by the constraints intrinsic to the definition of the minInf term in Equation (27). First, there is an additional constraint $I_{Q^{*}}\left({\bar{V}}_{1};{\bar{V}}_{2}|{\bar{V}}_{3}\right)\leq I_{P}\left({\bar{V}}_{1};{\bar{V}}_{2}|{\bar{V}}_{3}\right)$ , associated with the minimization, since P∈ΔP and $Q^{*}$ is a minimum. Second, there is a constraint $H_{P}\left({\bar{V}}_{S}\right)=H_{Q^{*}}\left({\bar{V}}_{S}\right)$ for any ${\bar{V}}_{S}$ that appears in one of the marginal distributions preserved in ΔP .

In the presence of additional constraints induced by a causal structure, the same constraints are imposed to *P* and $Q^{*}$ for those independencies associated with variables whose joint marginals are preserved. For example, for a causally-induced independence ${\bar{V}}_{1}⊥{\bar{V}}_{2}|{\bar{V}}_{3}$ , the constraints $I_{P}\left({\bar{V}}_{1};{\bar{V}}_{2}|{\bar{V}}_{3}\right)=0$ and $I_{Q^{*}}\left({\bar{V}}_{1};{\bar{V}}_{2}|{\bar{V}}_{3}\right)=0$ are imposed if $P({\bar{V}}_{1},{\bar{V}}_{2},{\bar{V}}_{3})$ is preserved in ΔP . Overall, the entropic vector associated with the original distribution *P* and the vector associated with $Q^{*}$ are coupled. Furthermore, the set of constraints that characterizes the accessible region for entropic vectors also includes the minInf DP inequalities. This is because the derivation of these DP inequalities results from the definitions in terms of the minimization operator (see proofs of Lemma 3 and Proposition 5), that is, the DP inequality holds specifically for distributions at the minimum. Without imposing the DP inequalities as constraints to bound the region accessible to entropic vectors, the entropic vector of $Q^{*}\left({\bar{V}}_{1},{\bar{V}}_{2},{\bar{V}}_{3}\right)$ could correspond to any distribution within ΔP fulfilling $I_{Q^{*}}\left({\bar{V}}_{1};{\bar{V}}_{2}|{\bar{V}}_{3}\right)\leq I_{P}\left({\bar{V}}_{1};{\bar{V}}_{2}|{\bar{V}}_{3}\right)$ , without corresponding to the minimum.

This type of coupling among entropic vectors does not uniquely result from constraints involving the original distribution *P*. Consider two families ΔP and $\Delta \tilde{P}$ , with $\Delta \tilde{P}\subseteq \Delta P$ , and a term $I_{Q}\left({\bar{V}}_{1};{\bar{V}}_{2}|{\bar{V}}_{3}\right)$ to be minimized. Consider distributions ${\tilde{Q}}^{*}$ and $Q^{*}$ that reach the minimum within $\Delta \tilde{P}$ and ΔP , respectively. Since $\Delta \tilde{P}\subseteq \Delta P$ , this means that there is a constraint $I_{{\tilde{Q}}^{*}}\left({\bar{V}}_{1};{\bar{V}}_{2}|{\bar{V}}_{3}\right)\geq I_{Q^{*}}\left({\bar{V}}_{1};{\bar{V}}_{2}|{\bar{V}}_{3}\right)$ . Furthermore, there are also constraints $H_{{\tilde{Q}}^{*}}\left({\bar{V}}_{S}\right)=H_{Q^{*}}\left({\bar{V}}_{S}\right)$ for any ${\bar{V}}_{S}$ that appears in the shared preserved marginals of ΔP and $\Delta \tilde{P}$ . The same happens with the additional constraints induced by a causal structure. If both ΔP and $\Delta \tilde{P}$ preserve the marginal distribution containing the variables associated with a causally-imposed conditional independence, this results in information terms with zero values, creating a further coupling between entropic vectors corresponding to distributions in the two families.

Overall, there is a coupling between entropic vectors, comprising the one of the original probability distribution and those of the probability distributions defined in terms of minimizations within families that preserve certain sets of marginals. The resulting set of constraints includes constraints of different types. First, the basic inequalities of Equation (26), which apply to all distributions. Second, inequalities related to the definition of the minInf terms within a family of distribution preserving a set of marginals. This includes equalities between entropies when two families of distributions share marginals that are preserved. It also includes inequalities between information terms when they result from the minimization within families that are one a subset of the other. Third, it includes causally-induced constraints. This includes equalities (information terms with zero value) that apply to the original distribution and to any minInf distribution that preserves the joint marginal of variables involved in a conditional independence. This implies the DP inequalities of minInf terms.

The region accessible to the vectors compatible with a certain causal structure can thus be characterized in two dual ways. Given the selection of *M* minInf distributions resulting from *M* minimizations, one possibility is to describe the region as a set of M+1 interdependent entropic vectors within the $R^{2^{n}}$ space of entropies. Another possibility is to define a $R^{\left(2^{n}\right)\left(M+1\right)}$ space in which the entropic vectors of the original joint distribution *P* and of the *M* additional joint distributions associated with the minInf terms are appended. The latter representation constructs the minInf Shannon entropy cone.

A formal characterization of the region of minInf Shannon entropy cones is beyond the aim of this paper. However, the considerations above suggest how an algorithmic entropic characterization of causal structures [14,24] can be extended to exploit the constraints that exist in minInf Shannon entropy cones. Since the constraints involving minInf terms also constitute a linear system of equalities and inequalities, the procedure used to derive testable inequalities by the marginalization of hidden variables [14] can be extended to include minInf terms. Note that the minimization operations involved in the identification of the minInf distributions are not to be solved as part of the linear system. They are reflected in the system by the inclusion of the constraints associated with the definition of the minInf terms. This guarantees that, after the marginalization, the reduced system will contain entropic inequalities that express relations between estimable minInf terms, which can then be tested. The minInf instrumental entropic inequalities introduced in previous sections are one example of inequalities that would be obtained with this algorithmic approach. The implementation of this extended procedure is left for future work.

### 3.7. Other Types of Entropic Inequalities with minInf Information Terms

The implementation of a procedure to algorithmically derive causally informative inequalities with minInf terms is out of the scope of this work. However, we here point to two other well-known types of causally informative entropic inequalities that can be extended thanks to the minInf DP inequalities introduced in Section 3.4. We do not aim to provide a full presentation of these entropic inequalities, but to reframe them in a form that allows appreciating their extensions.

The first type of inequalities that we extend is the Groups-Decomposition (GD) inequalities [25,26]. We keep the notation of [26] to facilitate the comparison. This type of inequalities relates the information that a collection of variables has about a set of target variables Y with a weighted sum of the information contained in different groups defined as subsets of that collection. Two subtypes of GD inequalities were introduced in [25]. The existence of an inequality of the first subtype (GD1) imposes certain conditions of independence between the groups and determines the weights based on the overlap between them. The second subtype (GD2) has no requirements of independence, but only applies to collections and groups that constitute ancestral sets, that is, sets including all ancestors of their members. Several extensions were introduced in [26], comprising a relaxation of the required conditions of independence for GD1 and more flexibility in the configuration of groups for GD2. These extensions also included a generalization to allow for conditioning sets and the use of the DP inequalities of Lemmas 1 and 3 to derive testable GD inequalities from collections containing hidden variables.

**Figure 3:**
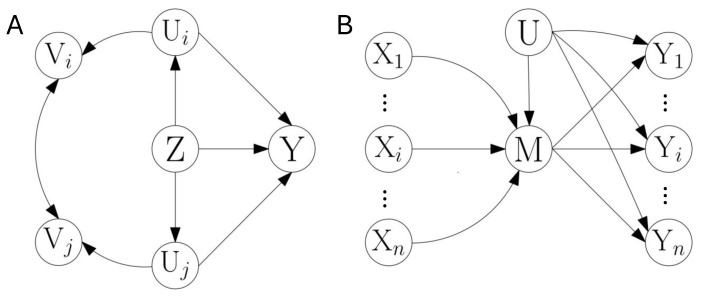
Other types of entropic inequalities with a form that allows applying minInf DP inequalities to add estimable information terms in the lower bound with the procedure developed in Section 3.4. (**A**) An example of causal structure associated with Groups-Decomposition (GD) inequalities [25,26]. The graph shows two representative groups $B_{i}=\left\{U_{i}\right\}$ , $B_{j}=\left\{U_{j}\right\}$ out of a collection $B_{\left[n\right]}=\left[B_{1},\ldots ,B_{n}\right]$ , with all groups having the same structure of connectivity. An entropic inequality of the form of Equation (28) holds. (**B**) Causal structure associated with the Information Causality (IC) inequality [23,31] in the case of classical systems. An entropic inequality of the form of Equation (29) holds.

We here present GD inequalities in a form that highlights how to apply the tools developed in Section 3.4, hence using minInf DP inequalities to increase their causal inference power. For simplicity, we restrict ourselves to GD1 inequalities, because the presentation of the second subtype is more mathematically heavy. We extend GD1 inequalities following Proposition 3 of [26], while an extension of GD2 analogously follows from their Theorem 2. For the purpose of this extension, we explicitly differentiate observable variables V and hidden variables U . The inequality considers a target set of variables Y and a conditioning set Z . It also considers a collection of variables $B_{\left[n\right]}=\left[B_{1},\ldots ,B_{n}\right]$ formed by *n* groups, possibly overlapping. Each group can contain observable and hidden variables, such that $B_{i}=\left\{V_{i},U_{i}\right\}$ . The GD1 inequality states that

(28) $$\begin{array}{cc} \; & H\left(Y|Z\right)\geq \sum_{i=1}^{n}\frac{1}{d_{B_{i}}}I\left(Y;B_{i}|Z\right)=\sum_{i=1}^{n}\frac{1}{d_{B_{i}}}\left[I\left(Y;V_{i}|Z\right)+I\left(Y;U_{i}|Z,V_{i}\right)\right], \end{array}$$

where $d_{B_{i}}-1$ is the number of groups that intersect with $B_{i}$ . The inequality holds if the groups fulfill the following conditions of independence: Given disjoint partitions $B_{i}=\left\{B_{i}^{\left(1\right)},B_{i}^{\left(2\right)}\right\}$ , $B_{i}^{\left(1\right)}⊥B_{j}^{\left(1\right)}\backslash B_{i}^{\left(1\right)}|Z$ and $B_{i}^{\left(2\right)}⊥B_{j}\backslash B_{i}^{\left(2\right)}|B_{i}^{\left(1\right)}Z$ , for all i≠j . For each group, the term $I(Y;V_{i}|Z)$ can be estimated, while the term $I(Y;U_{i}|Z,V_{i})$ is analogous to the terms with hidden variables that appear in Proposition 3. Ref. [26] used the DP inequalities of mutual information and unique information to derive tighter estimable lower bounds. An example of a causal structure for which this inequality is causally fulfilled is shown in Figure 3A. In this case, the structure of a Common Ancestors (CM) graph [24] is obtained after conditioning on *Z*, with all dependencies between observable variables mediated by hidden variables. A GD inequality exists selecting $B_{i}=B_{i}^{\left(1\right)}=\left\{U_{i}\right\}$ , such that $B_{i}^{\left(1\right)}⊥B_{j}^{\left(1\right)}|Z$ holds for all i≠j . The standard DP inequality is applied to each group, thanks to $Y⊥V_{i}|ZU_{i}$ , which leads to a testable GD inequality. Consider now the role of the terms $I(Y;U_{i}|Z,V_{i})$ if the structure of Figure 3A is embedded as part of a larger causal structure for which other minInf DP inequalities can be applied. In that case, analogously to Figure 1 and Figure 2, the terms $I(Y;U_{i}|Z,V_{i})$ can be the starting point to iteratively add estimable information terms at the lower bound of the inequality, with a procedure analogous to the one enabled by Propositions 3 and 6.

The second type of inequality to be generalized is the Information Causality (IC) inequality [23,31]. This inequality differs from the ones considered so far in that it contemplates a marginal scenario defined not only in terms of the presence of hidden variables, but also in terms of restrictions regarding which observable variables are jointly observable. The motivation of this marginal scenario is that the IC inequality was conceived to comprise also quantum systems. However, we here focus on its derivation for classical systems, since the consideration of quantum systems would require examining how to possibly adapt our results to the case in which Shannon entropy is substituted by von Neumann entropy [31]. In particular, we follow the generalization introduced by [31]. For classical systems, the derivation of this IC inequality can be understood in relation to the causal structure of Figure 3B. We have kept the notation used in [31] to facilitate a comparison with their derivations. The only exception is that we use *U* for the hidden variable, consistently with our previous derivations, while in [31] it corresponds to variable *B*. All variables $X=\{X_{1},\ldots ,X_{n}\}$ , $Y=\{Y_{1},\ldots ,Y_{n}\}$ , and *M* are observable, with *U* hidden. However, the marginal scenario is defined as imposing further constraints of observability, such that the variables in Y have mutually exclusive observability. Only marginal distributions of the form $p(X,M,Y_{i})$ are observable, for each $Y_{i}\in Y$ . Accordingly, for an inequality to be testable, it can only contain information terms estimable from these marginals. We now present the IC inequality in a form that highlights how it can be extended based on the tools developed in Section 3.4:

(29) $$\begin{array}{cc} \; & H\left(M\right)-H\left(X\right)+\sum_{i=1}^{n}H\left(X_{i}\right)\overset{\left(a\right)}{\geq }\left[I\left(X_{1};M\right)+\sum_{i=2}^{n}I\left(X_{i};X_{1},M\right)\right]+\\ & \left[I\left(X_{1};U|M\right)+\sum_{i=2}^{n}I\left(X_{i};U|X_{1},M\right)\right]\overset{\left(b\right)}{\geq }\left[I\left(X_{1};M\right)+\sum_{i=2}^{n}I\left(X_{i};X_{1},M\right)\right]+\\ & \left[I\left(X_{1};Y_{1}|M\right)+\sum_{i=2}^{n}I\left(X_{i};Y_{i}|X_{1},M\right)\right]+\left[I\left(X_{1};U|M,Y_{1}\right)+\sum_{i=2}^{n}I\left(X_{i};U|X_{1},M,Y_{i}\right)\right]. \end{array}$$

Note that the selection of $X_{1}$ is arbitrary and without loss of generality it can be replaced by any other variable in X . The detailed derivation of inequality (a) can be found in Equations (16)–(21) of [31] and follows from Lemma 2 in [24]. Apart from basic properties of entropy, the derivation relies on the causally-imposed independence X⊥U . On the other hand, inequality (b) is derived thanks to the DP inequalities of mutual information associated with $X_{1}⊥Y_{1}|UM$ and $X_{i}⊥Y_{i}|UMX_{1}$ , which provide observable lower bounds of the terms $I(X_{1};U|M)$ and $I(X_{i};U|X_{1},M)$ . More generally, these terms can be the starting point to iteratively add estimable information terms at the lower bound of the inequality, with a procedure analogous to the one enabled by Propositions 3 and 6. To highlight this, we have kept in the lower bound the nonestimable information terms that contain *U* and that are dropped in the final testable inequality of [31]. If the structure of Figure 3B is embedded as part of a larger causal structure such that other types of minInf DP inequalities can be applied, an iterative addition of observable information terms in the lower bound can proceed as in Proposition 6.

## 4. Discussion

In this work we have explored how causally informative entropic inequalities can be extended to contain minimum information (minInf) entropic terms. We have first examined how to combine the standard data processing (DP) inequality and a DP inequality for the maximum entropy unique information [26,29] to create new and tighter instrumental entropic inequalities [24]. In this way, we identified a procedure to recursively combine different types of DP inequalities to introduce additional observable terms in the lower bound of information terms that contain hidden variables. We then introduced a DP inequality for a general type of minInf information terms defined by information minimization within families of distributions that preserve sets of marginals shared with the original distribution. We have shown how to recursively apply these DP inequalities to exploit sets of independencies with different conditioning sets. We then have used this procedure to build minInf instrumental entropic inequalities that provide additional causal inference power. While our development of causally informative entropic inequalities uses as vector for its derivation the instrumental causal scenario, the procedure presented in Theorem 1 to recursively combine DP inequalities is general. We have exemplified this for two other types of entropic inequalities, namely the Groups-Decomposition inequality [25,26], and the Information Causality inequality [23,31].

More generally, we have also reframed the use of minInf entropic terms to derive causally informative entropic inequalities from a geometrical perspective [22]. Entropic inequalities can be systematically derived as a marginalization problem [14]. To derive causally informative inequalities that only involve entropic terms of the original distribution, this marginalization operates on the set of constraints that defines the Shannon entropy cone [22], in combination with the additional conditional independence constraints imposed by the causal structure. We have indicated that, in order to incorporate minInf entropic terms in this geometrical approach, Shannon entropy cones can be extended to not only contain entropic terms from the original distribution, but to jointly combine the minInf entropic terms associated with a set of families of distributions that share sets of marginals with it. The derivation of causally informative inequalities with minInf terms can then proceed analogously as a marginalization problem, now departing from a set of constraints that comprises those that the conditional independencies in the causal structure impose to the minInf entropic terms, such as the minInf DP inequalities we have derived. While we have conceptualized this procedure, its implementation in a linear programming algorithm [14,22] remains out of the scope of this work.

For the instrumental causal scenario, we have provided a range of examples of causal structures for which causal inference power is increased with the new tests, through the examples of the Figures in the main text and further examples in Appendix F and Appendix I. With an increasing number of causally informative inequality tests, an important question is how to determine minimal sets of tests that preserve the overall causal inference power. For this purpose, we have stated a criterion to determine when a new inequality test can add causal inference power to a set of tests (Appendix B). We have used this criterion to derive a hierarchy for instrumental inequalities with multivariate instrumental sets that encompasses the comparison of certain types of tests. On the other hand, we have also provided examples for which various minInf entropic inequalities are complementary among them, potentially providing each additional power.

Our contribution has focused on the derivation of causally informative minInf entropic inequalities for which the involved families of distributions are determined by constraints on shared marginals. Alternatively, the minInf terms could be determined comprising other types of constraints. Specifically for the unique information term developed within the decomposition of mutual information into redundant, unique, and synergistic components, alternative approaches [39,41,45] define unique information within families that combine other constraints. Future work should determine if alternative entropic inequalities are derived with these measures and the degree to which they contribute to increase causal inference power.

Our approach can also benefit the study of causal interactions in dynamical systems [14,46]. High-dimensional multivariate dynamical processes appear in many domains of interest, such as brain dynamics, e.g., [47,48,49] or econometrics [50,51]. For time-series, methods of causal inference both in the temporal [52] and spectral domain [53,54] predominantly rely on the detection of conditional independencies between observable signals, and hence are affected by hidden variables due to the partial observability of complex systems. Our treatment of the instrumental causal scenario suggests that our methods can help in these cases, given that concatenated instrumental-like causal motifs frequently appear in temporal dynamics, as represented for example by autoregressive moving-average models [52]. The unique information measure has already been applied to neural data, e.g., [55,56]. However, applicability of entropic inequalities with minInf terms to dynamical systems will require further study of the scalability of our methods to high-dimensional data. The applicability of entropic inequalities to time-series will also need to incorporate further assumptions such as stationarity [57] to make the approach operational.

Beyond applications to classical systems, it remains to be seen how to adapt the formulation of minInf entropic inequalities for causal inference in the quantum domain [16,31,58,59]. In our work, we have considered how to extend Information Causality inequalities [23] using minInf DP inequalities, but we focused on their formulation for classical systems [31]. Quantum mutual information defined in terms of von Neumann entropy [60] fulfills the standard DP inequality as well as the chain rule property, which is involved in the derivation of Theorem 1. Nonetheless, future work is required to pursue an adaptation to quantum systems.

In this work we have focused on causally informative entropic inequalities. We have mostly focused on the instrumental causal scenario to introduce new minInf entropic inequalities and examine them jointly with standard inequalities constructed with multivariate instrumental sets. More broadly, an important question regards the embedding of this type of causally informative entropic inequalities with other approaches to test the compatibility of causal structures with data [61,62,63]. The fact that the new inequalities appear as constraints only in the extended minInf Shannon entropy cones indicates that they cannot be reduced to constraints in the standard Shannon entropy cone of the original joint distribution. However, a separate question is how to construct minimal sets of inequality constraints with equivalent inference power, and how these sets would combine or prioritize the use of minInf tests and tests that operate in an inflated standard Shannon entropy cone [64]. The integration of different families of tests under the criterion that determines when the addition of a new test increases the causal inference power of a set of tests stands as a goal to determine the boundaries of distinguishably between causal structures from data.

## Figures and Tables

**Table 1:** Sequential relaxation of preserved marginals when combining minInf DP inequalities. (i) Combination of the DP inequality of mutual information and of unique information in Figure 2A. (ii) Combination of the DP inequality of mutual information and of unique information in Figure 2C. (iii) Combination with an additional minInf DP inequality in Figure 2C.

	Prior Use of a DP Inequality	Relaxation of Preserved Marginals	Subsequent Use of a DP Inequality
(i)	$P(Z,U,Y_{1},W_{1},W_{2},X)$	$\left\{P\left(Z,U,W_{1},X\right),P\left(Z,Y_{1},W_{1},W_{2},X\right)\right\}$	$\left\{P\left(Z,U,Y_{2},W_{1},X\right),P\left(Z,Y_{1},W_{1},W_{2},X\right)\right\}$
	$Z⊥Y_{1}|UXW_{1}W_{2}$		$Z⊥Y_{2}|UXW_{1}$
(ii)	$P(Z,U,Y_{1},W_{2},W_{3},X)$	$\left\{P\left(Z,U,W_{3},X\right),P\left(Z,Y_{1},W_{2},W_{3},X\right)\right\}$	$\left\{P\left(Z,U,Y_{2},W_{3},X\right),P\left(Z,Y_{1},W_{2},W_{3},X\right)\right\}$
	$Z⊥Y_{1}|UXW_{2}W_{3}$		$Z⊥Y_{2}|UXW_{3}$
(iii)	$\{P\left(Z,U,Y_{2},W_{3},X\right),$	$\{P\left(Z,U,Y_{2},X\right),P\left(Z,Y_{2},W_{3},X\right),$	$\{P\left(Z,U,Y_{3},Y_{2},X\right),P\left(Z,Y_{2},W_{3},X\right),$
	$P(Z,Y_{1},W_{2},W_{3},X)\}$	$P(Z,Y_{1},W_{2},W_{3},X)\}$	$P(Z,Y_{1},W_{2},W_{3},X)\}$
	$Z⊥Y_{2}|UXW_{3}$		$Z⊥Y_{3}|UXY_{2}$

**Figure A1:**
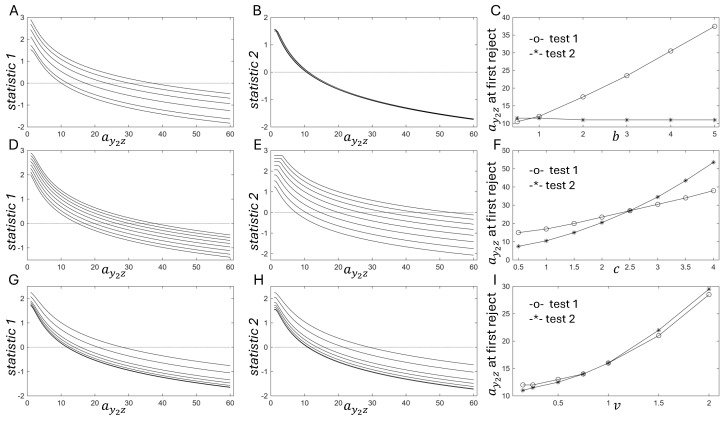
Examples of applications of entropic inequalities to reject the compatibility of causal structures with data. The fulfillment of inequalities in Equation (A4) is tested for a causal structure like in Figure 2A, with the addition of a connection $X↔W_{2}$ and connections $Z\to Y_{1}$ , $Z\to Y_{2}$ . Each row illustrates changes in the results of the entropic inequality tests for a concrete set of modifications of the default configuration, as it is described in the main text. The first column shows the values of the statistic of Test 1, corresponding to the difference between the l.h.s. and r.h.s. of Equation (A4a). The second column shows the values of the statistic of Test 2, the difference between the l.h.s. and r.h.s. of Equation (A4b). The third column shows the minimal strength of $a_{y_{2}z}$ that results in a negative statistic and hence in the violation of a test and the rejection of causal structures that causally impose its fulfillment. Note that the concrete configuration to which each line in the first and second column belongs is retrieved from the value of the parameters in the abscissas of the third column.

**Figure A3:**
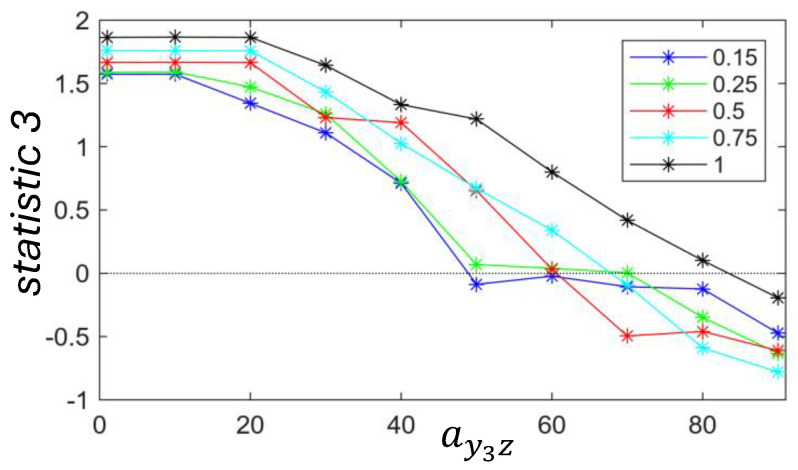
Examples of violations of the instrumental entropic inequality of Equation (25). Multivariate Gaussian systems are generated as described in Appendix H. The statistic of the test associated with Equation (A16) is displayed as a function of the strength $a_{y_{3}z}$ of the connection $Z\to Y_{3}$ for different standard deviations *v* of the exogenous noises of the variables.

## Data Availability

No datasets used. Code used can be provided upon request.

## Appendix A. Proofs of Monotonicity and Data Processing Inequality of the Unique Information

**Proof of Lemma** **2.** Consider the distribution $P_{DD^{\prime }}\equiv P\left(\bar{Z},D,D^{\prime },D_{2},O_{1}\right)$ and its marginal $P_{D}\equiv P\left(\bar{Z},D,D_{2},O_{1}\right)$ . Consider the family $\Delta P_{DD^{\prime }}$ of distributions that preserve $P(\bar{Z},D,D^{\prime },O_{1})$ and $P(\bar{Z},D_{2},O_{1})$ , and the family $\Delta P_{D}$ that preserves $P(\bar{Z},D,O_{1})$ and $P(\bar{Z},D_{2},O_{1})$ . Consider any distribution $Q_{DD^{\prime }}\in \Delta P_{DD^{\prime }}$ and its marginal $Q_{D}$ on $\left\{\bar{Z},D,D_{2},O_{1}\right\}$ . Then $Q_{D}\in \Delta P_{D}$ . By monotonicity of the mutual information, the information $I_{Q_{DD^{\prime }}}\left(\bar{Z};D|D_{2},O_{1}\right)$ is lower than or equal to $I_{Q_{DD^{\prime }}}\left(\bar{Z};D,D^{\prime }|D_{2},O_{1}\right)$ . Since $I_{Q_{DD^{\prime }}}\left(\bar{Z};D|D_{2},O_{1}\right)$ does not have $D^{\prime }$ as an argument, it is equal to $I_{Q_{D}}\left(\bar{Z};D|D_{2},O_{1}\right)$ . Since this holds for any distribution in $\Delta P_{DD^{\prime }}$ , it holds in particular for the distribution $Q_{DD^{\prime }}^{*}$ that minimizes $I(\bar{Z};D,D^{\prime }|D_{2},O_{1})$ in $\Delta P_{DD^{\prime }}$ . Since $Q_{D}^{*}$ belongs to $\Delta P_{D}$ , the minimum of $I(\bar{Z};D|D_{2},O_{1})$ in $\Delta P_{D}$ is equal to or smaller than $I_{Q_{D}^{*}}\left(\bar{Z};D|D_{2},O_{1}\right)$ and hence equal to or smaller than $I_{Q_{DD^{\prime }}^{*}}\left(\bar{Z};D,D^{\prime }|D_{2},O_{1}\right)$ . □

**Proof of Lemma** **3.** Let $P_{DD^{\prime }}\equiv P\left(\bar{Z},D,D^{\prime },E\right)$ , with $E=\{D_{2},O_{1}\}$ , be the original distribution of the variables and define $\Delta P_{DD^{\prime }}$ as the family of distributions on $\left\{\bar{Z},D,D^{\prime },E\right\}$ that preserve the two marginals $P(\bar{Z},D,D^{\prime },O_{1})$ and $P(\bar{Z},E)$ . Let $P_{D}\equiv P\left(\bar{Z},D,E\right)$ be the marginal of $P_{DD^{\prime }}$ and $\Delta P_{D}$ be the family of distributions that preserve the marginals $P(\bar{Z},D,O_{1})$ and $P(\bar{Z},E)$ . By definition of unique information (Equation (5))

(A1)
$$\begin{array}{cc} \; & I(\bar{Z};D,D^{\prime }\backslash \backslash D_{2}\left|O_{1}\right)\equiv \min_{Q_{DD^{\prime }}\in \Delta P_{DD^{\prime }}}I_{Q_{DD^{\prime }}}\left(\bar{Z};D,D^{\prime }|E\right)\overset{\left(a\right)}{=}\min_{Q_{DD^{\prime }}\in \Delta P_{DD^{\prime }}}\left[I_{Q_{DD^{\prime }}}\left(\bar{Z};D|E\right)+\right.\\ & \left.I_{Q_{DD^{\prime }}}\left(\bar{Z};D^{\prime }|D,E\right)\right]\overset{\left(b\right)}{=}\min_{Q_{DD^{\prime }}\in \Delta P_{DD^{\prime }}}\left[I_{Q_{D}}\left(\bar{Z};D|E\right)+I_{Q_{DD^{\prime }}}\left(\bar{Z};D^{\prime }|D,E\right)\right]. \end{array}$$

Equality (a) follows from the chain rule of mutual information. Equality (b) holds because $I_{Q_{DD^{\prime }}}\left(\bar{Z};D|E\right)$ does not depend on $D^{\prime }$ and can be calculated with $Q_{D}$ , marginalizing $Q_{DD^{\prime }}$ on $D^{\prime }$ . Note that $Q_{D}\in \Delta P_{D}$ . Since $I_{P_{DD^{\prime }}}\left(\bar{Z};D^{\prime }|D,O_{1}\right)=0$ , $P(\bar{Z},D,D^{\prime },O_{1})$ factorizes as $P\left(D^{\prime }|D,O_{1}\right)P\left(\bar{Z},D,O_{1}\right)$ . For any distribution ${\tilde{Q}}_{D}\in \Delta P_{D}$ , which preserves $P(\bar{Z},E)$ and $P(\bar{Z},D,O_{1})$ , a distribution can be constructed as ${\tilde{Q}}_{DD^{\prime }}\equiv P\left(D^{\prime }|D,O_{1}\right){\tilde{Q}}_{D}$ , such that ${\tilde{Q}}_{DD^{\prime }}\in \Delta P_{DD^{\prime }}$ , since ${\tilde{Q}}_{DD^{\prime }}$ continues to preserve $P(\bar{Z},E)$ , and $P(\bar{Z},D,D^{\prime },O_{1})$ is preserved by construction. Also by construction, $I_{{\tilde{Q}}_{DD^{\prime }}}\left(\bar{Z},D_{2};D^{\prime }|D,O_{1}\right)=0$ and hence $I_{{\tilde{Q}}_{DD^{\prime }}}\left(\bar{Z};D^{\prime }|D,E\right)=0$ for any ${\tilde{Q}}_{DD^{\prime }}$ created from any ${\tilde{Q}}_{D}\in \Delta P_{D}$ . In particular, this holds for the distribution ${\tilde{Q}}_{DD^{\prime }}^{*}$ constructed from ${\tilde{Q}}_{D}^{*}$ that minimizes $I_{{\tilde{Q}}_{D}}\left(\bar{Z};D|E\right)$ , which determines $I(\bar{Z};D\backslash \backslash D_{2}|O_{1})$ . The distribution ${\tilde{Q}}_{DD^{\prime }}^{*}$ minimizes the first term in the r.h.s. of Equation (A1) and, given the nonnegativity of mutual information, it also minimizes the second term, hence providing the minimum in $\Delta P_{DD^{\prime }}$ . Accordingly, $I(\bar{Z};D,D^{\prime }\backslash \backslash D_{2}|O_{1})$ $=I(\bar{Z};D\backslash \backslash D_{2}|O_{1})$ . The monotonicity of the unique information in its second argument (Lemma 2) leads to $I(\bar{Z};D,D^{\prime }\backslash \backslash D_{2}|O_{1})\geq I(\bar{Z};D^{\prime }\backslash \backslash D_{2}\left|O_{1}\right)$ . □

## Appendix B. Sets of Entropic Inequality Tests with Complementary Causal Inference Power

A set of causal inequality tests contains complementary inequalities if each inequality can potentially contribute to increase the causal inference power, that is, each test can potentially discard a causal structure not rejected by the other tests. We here formalize this idea:

**Remark A1** 
(Lack of additional causal inference power of an inequality for causal structures compatible with a set of independencies). *Consider a set of observable variables V that fulfill a set of testable conditional independencies $I_{V}$ . Consider a set of causal inequality tests $T_{V}$ that provide sufficient conditions to potentially reject some causal structure from the set $G(I_{V})$* of causal structures compatible with *$I_{V}$ . An inequality test $t_{V}\in T_{V}$ does not provide additional causal inference power if and only if: (i) All the causal structures in $G(I_{V})$ causally impose the fulfillment of the inequality in $t_{V}$ or (ii) The inequality is not causally imposed by all causal structures in $G(I_{V})$ but for each causal structure in $G(I_{V})$ that causally imposes the fulfillment of the inequality in $t_{V}$ there is another subset of inequality tests $T_{V}^{\prime }\subset T_{V}$ (not necessarily the same for all causal structures) such that the inequalities in $T_{V}^{\prime }$ are also causally imposed and the statistical rejection of $t_{V}$ from data is sufficient for the rejection of at least a $t_{V}^{\prime }\in T_{V}^{\prime }$ from data.*

Remark A1 indicates that a test $t_{V}$ can only increase causal inference power if it can potentially discard a causal structure that could not otherwise be discarded by other tests. This can occur in two ways. First, there is some causal structure for which no other inequality is causally imposed by that structure. In this case, $t_{V}$ is the only test that if rejected allows discarding the causal structure. Second, for all causal structures that impose the fulfillment of $t_{V}$ there is also another subset $T_{V}^{\prime }$ whose fulfillment is imposed by the causal structure. In this case, $t_{V}$ only adds power if it exists a causal structure, other than the ones for which $t_{V}$ is causally imposed, such that potentially no other inequality from $T_{V}^{\prime }$ is violated when tested in data generated with that causal structure, while $t_{V}$ is violated. On the other hand, if the violation of $t_{V}$ always leads to the violation of some other $t_{V}^{\prime }\in T_{V}^{\prime }$ , there is no context in which $t_{V}$ allows discarding a causal structure that is not already discarded by another test.

Note that Remark A1 does not consider the estimation properties of the information measures, and it is out of the focus of our work to address estimation considerations. For example, consider that $t_{V}$ has in its upper bound the sum of upper bounds from a subset $T_{V}^{\prime }$ of causally-imposed inequalities, and in its lower bound the sum of the corresponding lower bounds. Then the violation of $t_{V}$ implies the violation of at least a test $t_{V}^{\prime }\in T_{V}^{\prime }$ , and hence this type of test consisting on additions of tests never adds causal inference power for causal graphs that causally impose all inequalities in $T_{V}^{\prime }$ . This does not preclude the fact that, despite this lack of additional power, a test consisting on an addition of tests may in practice still be useful when considering the estimation properties of the measures, the size of the data set, and practical considerations about the false rejection rate and false acceptance rate that are desirable.

## Appendix C. A Further Comparison of Instrumental Inequalities in Figure B

We here analyze in further detail Figure 2B in order to provide an example in which, in contrast to Figure 2A, no additional causal inference power is gained combining the DP inequality of conditional mutual information and the one of unique information. As mentioned in Section 3.3, we compare the inequality resulting from Z=Z , $B_{0}=\left\{W_{1},W_{2}\right\}$ and the one resulting from $Z=\{Z,W_{2}\}$ , $B_{0}=\left\{W_{1}\right\}$ . The obtained inequalities are:

(A2a)
$$\begin{array}{cc} \; & H\left(X|W_{1},W_{2}\right)\geq I\left(Z;X|W_{1},W_{2}\right)+I\left(Z;Y_{1}|W_{1},W_{2},X\right)+I(Z;Y_{2}\backslash \backslash W_{2}\left|W_{1},X,Y_{1}\right), \end{array}$$

(A2b)
$$\begin{array}{cc} \; & H\left(X|W_{1}\right)\geq I\left(Z,W_{2};X|W_{1}\right)+I\left(Z,W_{2};Y_{1}|W_{1},X\right)+I\left(Z;Y_{2}|W_{1},X,Y_{1}\right). \end{array}$$

Inequality A2a corresponds to Equation (17), with Z=Z , $B_{0}=\left\{W_{1},W_{2}\right\}$ , $B_{1}=W_{1}$ , $B_{2}=W_{2}$ , and $\bar{Y}=\emptyset$ . Inequality A2b is derived with $Z=\{Z,W_{2}\}$ , $B_{0}=\left\{W_{1}\right\}$ , starting from $I(Z,W_{2};U|W_{1},X)$ instead of from $I(Z;U|W_{1},W_{2},X)$ . In Equation (A2a), the term with $Y_{1}$ is introduced thanks to $Z⊥Y_{1}|UXW_{1}W_{2}$ , while in Equation (A2b) the term with $Y_{1}$ is introduced thanks to $\left\{Z,W_{2}\right\}⊥Y_{1}|UXW_{1}$ . Both terms are obtained with the DP inequality of conditional mutual information. The difference appears in the derivation of the term with $Y_{2}$ . We have seen in the proof of Proposition 4 that in Equation (A2a) this term is obtained thanks to the DP inequality of unique information, removing $W_{2}$ from the conditioning set to exploit $Z⊥Y_{2}|UXW_{1}Y_{1}$ , oppositely to Z⊥Y2|UXW1W2Y1 . In contrast, in Equation (A2b), the term with $Y_{2}$ is obtained directly from the marginalization of $W_{2}$ in $I(Z,W_{2};U|W_{1},X,Y_{1})$ , which allows applying again the DP inequality of conditional mutual information to exploit $Z⊥Y_{2}|UXW_{1}Y_{1}$ . As expected, given the invariance of the upper bound to an exchange of variables between Z and $B_{0}$ , the same upper bound $H(X|W_{1},W_{2},Z)$ is obtained in Equation (A2a,b) after moving the first term of the r.h.s. to the l.h.s. On the other hand, $I(Z;Y_{1}|W_{1},W_{2},X)$ is smaller or equal than $I(Z,W_{2};Y_{1}|W_{1},X)$ by the chain rule of mutual information, and $I(Z;Y_{2}\backslash \backslash W_{2}|W_{1},X,Y_{1})$ is smaller or equal than $I(Z;Y_{2}|W_{1},X,Y_{1})$ by construction of the unique information (Equation (6)). Therefore, the inequality of Equation (A2b) is always violated when the one of Equation (A2a) is violated.

This analysis highlights that in Figure 2A it is the fact that $Z⊥Y_{1}|UXW_{1}W_{2}$ holds but Z⊥Y1|UXW1 what introduces the necessity to use the DP inequality of the unique information combined with the one of the conditional mutual information. Here, since $\left\{Z,W_{2}\right\}⊥Y_{1}|UXW_{1}$ holds, there is no need to condition on $W_{2}$ to introduce the term with $Y_{1}$ , and hence no need to remove $W_{2}$ from the conditioning set using the unique information DP inequality to exploit $Z⊥Y_{2}|UXW_{1}$ . Overall, $\left\{Z,W_{2}\right\}⊥Y_{1}|UXW_{1}$ and $Z⊥Y_{2}|UXW_{1}$ are combined through the marginalization of $W_{2}$ , while the conditioning sets in $Z⊥Y_{1}|UXW_{1}W_{2}$ and $Z⊥Y_{2}|UXW_{1}$ require the combination of the different types of DP inequalities.

## Appendix D. Estimation and Numerical Examples of Entropic Inequalities with Unique Information Terms

In Section 3.3 we have discussed the examples of causal structures of Figure 2A,B, which causally impose instrumental entropic inequalities of the form of Proposition 4. Here we provide some numerical examples of violations of the inequalities, that is, examples in which any causal structure that causally imposes their fulfillment can be discarded as the generative structure that underlies the observed variables.

Apart from the standard estimation of entropy and mutual information terms, a test of the form of Equation (17) also requires the estimation of the maximum entropy unique information. In general, the estimation of minInf information terms corresponds to a constrained minimization problem, where the constraints on the preservation of the marginals constitute a set of affine equality constraints. Therefore, whether the optimization problem is convex or non-convex depends on whether the mutual information term to be minimized is a convex function of the probability distributions within the family ΔP [65].

Specifically for the case of the unique information (Equation (5)), the mutual information minimization problem on $I(\bar{Z};D_{1}|O_{1},D_{2})$ can be recast as an entropy maximization problem since the constraints of ΔP impose the preservation of $P(\bar{Z},D_{1},O_{1})$ and $P(\bar{Z},E)$ , with $E=\{D_{2},O_{1}\}$ . Concretely, preserving $P(\bar{Z},E)$ makes the entropy $H(\bar{Z}|E)$ constant within ΔP , and hence minimizing $I(\bar{Z};D_{1}|O_{1},D_{2})$ corresponds to maximizing $H(\bar{Z}|E,D_{1})$ . This conditional entropy is concave on ΔP [29], and hence the constrained minimization of the mutual information is a convex optimization problem, which guarantees convergence towards a global minimum. Several software packages are available to numerically estimate the maximum entropy unique information [66] (https://github.com/dit/dit (accessed on 12 April 2026)), ref. [67] (https://github.com/Abzinger/MAXENT3D_PID (accessed on 12 April 2026)), or [55] (https://github.com/epiasini/SubPID/ (accessed on 12 April 2026)).

Apart from applications of convex optimization tools to estimate unique information terms, for some specific types of multivariate probability distributions, such as Gaussian distributions, expressions of the unique information have been analytically derived [68,69] in terms of standard mutual information terms. We will here focus on these cases to facilitate the examination of numerical examples. As we will further discuss in Appendix G, the estimation of other minInf terms that are not the unique information constitutes a non-convex optimization problem. By concentrating on Gaussian distributions we will provide some numerical examples also including other minInf terms, hence examining the effect of iteratively adding more terms to an entropic inequality, as it is done in Equation (25). We note that the focus of our work is the theoretical derivation of data processing inequalities and entropic inequalities with minInf information terms, and that the full implementation of numerical optimization tools to estimate these terms is beyond our scope.

For a multivariate Gaussian system with a univariate target variable $\bar{Z}$ it has been shown [68] that the maximum entropy unique information $I(\bar{Z};D_{1}\backslash \backslash D_{2}|O_{1})$ is determined in terms of the mutual information terms $I(\bar{Z};D_{1}|O_{1})$ and $I(\bar{Z};D_{2}|O_{1})$ . In more detail, for ΔP that preserves the marginals $P(\bar{Z},D_{1},O_{1})$ and $P(\bar{Z},E)$ , with $E=\{D_{2},O_{1}\}$ , in this case the unique information can be expressed as

(A3)
$$\begin{array}{c} I(\bar{Z};D_{1}\backslash \backslash D_{2}\left|O_{1}\right)=\min_{Q\in \Delta P}I_{Q}\left(\bar{Z};D_{1}|E\right)=\max\left\{I\left(\bar{Z};D_{1}|O_{1}\right)-I\left(\bar{Z};D_{2}|O_{1}\right),0\right\}, \end{array}$$

that is, $I(\bar{Z};D_{1}\backslash \backslash D_{2}|O_{1})$ is the additional information that $D_{1}$ has, or zero if $D_{2}$ has more information than $D_{1}$ . Furthermore, for Gaussian variables entropy terms are determined by the second-order moments of the distributions, e.g., [70]. Concretely, the terms that appear in an instrumental entropic inequality of the form of Equation (17) are such that the conditional entropy only depends on the determinant of the corresponding conditional covariance matrix and the information terms are determined by corresponding partial correlation coefficients [27].

To illustrate the additional causal inference power gained with entropic inequalities of the form of Proposition 4, we analyze a causal structure as in Figure 2A, with the addition of a hidden confounder $X↔W_{2}$ . For simplicity, $W_{1}=\emptyset$ is chosen, to avoid an additional conditioning variable. For this structure, we compare two entropic inequalities that are causally imposed:

(A4a)
$$\begin{array}{cc} H(X|Z)\geq & I(Z;Y_{2}|X) \end{array}$$

(A4b)
$$\begin{array}{cc} H(X|Z,W_{2})\geq & I\left(Z;Y_{1}|X,W_{2}\right)+I(Z;Y_{2}\backslash \backslash \left\{W_{2},Y_{1}\right\}\left|X\right)=\\ & I\left(Z;Y_{1}|X,W_{2}\right)+\max\left\{I\left(Z;Y_{2}|X\right)-I\left(Z;W_{2},Y_{1}|X\right),0\right\}. \end{array}$$

The inequality of Equation (A4a) is a standard entropic inequality that follows from Z⊥U and $Z⊥Y_{2}|UX$ . The inequality of Equation (A4b) is an entropic inequality of the form of Equation (17) that follows from $Z⊥U|W_{2}$ , $Z⊥Y_{2}|UX$ , and $Z⊥Y_{1}|UXW_{2}$ . We will refer to the tests associated with these inequalities as Test 1 and 2, respectively. The last expression at the r.h.s. of Equation (A4b) is specific for Gaussian variables, following Equation (A3). When $I(Z;Y_{2}|X)$ is bigger than $I(Z;W_{2},Y_{1}|X)$ the inequality of Equation (A4b) has the form

(A5)
$$\begin{array}{c} H\left(X|Z,W_{2}\right)\geq I\left(Z;Y_{2}|X\right)-I\left(Z;W_{2}|X\right). \end{array}$$

Comparing Equations (A4a) and (A5), we see that for Gaussian variables with a univariate Z the r.h.s. is always smaller in Test 2. It is for this reason that we here examine a causal structure with the additional connection $X↔W_{2}$ , since in Figure 2A $W_{2}⊥X|Z$ results in $H\left(X|Z,W_{2}\right)=H\left(X|Z\right)$ and Test 2 would always have less power than Test 1. Note that this relation between the tests is specific of the form of Test 2 in this Gaussian case.

In Figure A1 we compare the results of Tests 1 and 2 when additional connections $Z\to Y_{1}$ and $Z\to Y_{2}$ are added. The addition of the connection $Z\to Y_{2}$ leads to Z⊥Y2|UX , and hence Test 1 from Equation (A4a) is not causally fulfilled and can be violated. Similarly, either the addition of $Z\to Y_{2}$ or $Z\to Y_{1}$ can lead to the violation of Test 2 caused by Z⊥Y2|UX or Z⊥Y1|UXW2 .

We generate the variables as a system of linear equations with Gaussian noise. Given the large number of parameters in the system, we keep fixed the values of most parameters and examine concrete settings in which the strength of the connection $Z\to Y_{2}$ , determined by the coefficient $a_{y_{2}z}$ , is changed together with a single other parameter. In more detail, we set a default configuration in which all coefficients in the system are set to 1. The standard deviation of all hidden confounders is also set to 1, and their mean set to zero. Apart from *Z*, that has no parents, all other nodes are generated having also some exogenous independent noises, all of which are generated with mean zero and by default standard deviation 0.25. In each row of Figure A1 we explore different modifications of this default configuration. In all rows the coefficient $a_{y_{2}z}$ is changed from 1 to 50 with 0.5 increments. In the first row, we also modify the strength of the confounder $U^{\prime }$ in $X↔W_{2}$ , choosing $b\equiv a_{xu^{\prime }}=a_{w_{2}u^{\prime }}$ . The explored values appear in the abscissa of Figure A1C. In the second row, we set b=2 and modify the strength of connections Z→X , $Z\to W_{2}$ , and U→X , choosing $c\equiv a_{xz}=a_{w_{2}z}=a_{xu}$ . The explored values appear in the abscissa of Figure A1F. In the third row, the default configuration is modified only changing the standard deviation of the exogenous noises (*v*), which was fixed to 0.25. The explored values appear in the abscissa of Figure A1I.

Figure A1 provides numerical examples of how Test 2, the entropic inequality that includes the unique information term, does increase causal inference power. We have chosen configurations in which the strength of $a_{y_{1}z}=1$ is not enough by itself for a rejection in Test 2. This is reflected in the positive values of the tests when $a_{y_{2}z}$ is small, and indicates that for none of all the configuration studied Test 2 is rejected if removing the term $I(Z;Y_{2}\backslash \backslash \left\{W_{2},Y_{1}\right\}|X)$ from the r.h.s. of the inequality. Accordingly, all obtained negative values of Test 2, and hence the additional causal power, come from the addition of the unique information term. We can compare the rejections of Test 1 and 2 when increasing the strength of $a_{y_{2}z}$ . We see in Figure A1C that for any value of *b* higher than 1 Test 2 is more powerful than Test 1. Also in Figure A1F,I Test 2 is more powerful than Test 1 for some range of the explored configurations. In all these cases, causal information is gained for configurations for which Test 1 would not allow rejecting the causal structures that result in $Z⊥Y_{2}|UX$ , because the strength of $a_{y_{2}z}$ is not sufficiently high.

In Appendix H we will resume this analysis to illustrate how additional causal inference power can be gained incorporating additional minInf terms. For this purpose, in Appendix G we address the estimation of other minInf terms.

## Appendix E. Proof of Theorem 1

We here provide the proof of Theorem 1. As a preliminary, we introduce a chain rule inequality for minInf mutual information terms.

**Lemma A1** 
(Chain rule inequality for minInf mutual information terms). *Given sets of variables $V_{1}$ , $V_{2}$ , $V_{3}$ , and E , and a family of distributions ΔP preserving some marginals of the distribution $P(V_{1},V_{2},V_{3},E)$ ,*

(A6)
$$\begin{array}{c} \min_{Q\in \Delta P}I_{Q}\left(V_{1};V_{2},V_{3}|E\right)\geq \min_{Q\in \Delta P}I_{Q}\left(V_{1};V_{2}|E\right)+\min_{Q\in \Delta P}I_{Q}\left(V_{1};V_{3}|E,V_{2}\right). \end{array}$$

**Proof. ** The chain rule inequality follows from the chain rule equality that applies to all distributions Q∈ΔP and the fact that the sum of the minima is smaller than or equal to the minimum of the sum. □

Note that Lemma A1 holds independently of which is the set of marginals to be preserved in ΔP . The mutual information is symmetrical in its first and second argument, and the chain rule equality of mutual information is equally applied to the first or second argument. This symmetry implies that the chain rule inequality of Lemma A1 also holds when used to separate variables in the first or second argument. This is the case even if the definition of which marginals are preserved breaks the symmetry between the arguments, e.g., as it happens in the definition of the unique information, which identifies the first argument as the set of target variables.

We now prove Theorem 1.

**Proof of Theorem** **1.** We first consider the case of k=1 when ${\bar{B}}_{1}^{\prime }=\left\{A_{0},{\bar{B}}_{0}\right\}=E$ , which leads to ${\bar{B}}_{1}=\left\{E,{\overset{ˇ}{Z}}_{1}\right\}=C_{1}$ . The family $\Delta P_{0}$ preserves $P(\bar{Z},\bar{U},E)$ , given ${\bar{Z}}_{0}=\bar{Z}$ , ${\bar{U}}_{0}=\bar{U}$ . On the other hand, $\Delta P_{1}$ preserves $P({\bar{Z}}_{1},\bar{U},A_{1},E,{\overset{ˇ}{Z}}_{1})$ , given ${\bar{U}}_{1}=\bar{U}$ . Furthermore, $A^{\left[0\right]}=\emptyset$ and ${\overset{ˇ}{Z}}^{\left[0\right]}=\emptyset$ , and hence at the l.h.s. of Equation (23) for k = 1, $\min_{Q\in \Delta P_{0}}I_{Q}\left({\bar{Z}}_{0};{\bar{U}}_{0}|E,A^{\left[0\right]},{\overset{ˇ}{Z}}^{\left[0\right]}\right)=I\left(\bar{Z};\bar{U}|E\right)$ . Since $\left\{{\bar{Z}}_{1},{\overset{ˇ}{Z}}_{1}\right\}\subseteq \bar{Z}$ , the chain rule of mutual information guarantees that $I\left(\bar{Z};\bar{U}|E\right)\geq I\left({\bar{Z}}_{1};\bar{U}|E,{\overset{ˇ}{Z}}_{1}\right)$ . Subsequently, the standard DP inequality of Lemma 1 allows adding $A_{1}$ to $\bar{U}$ given the independence ${\bar{Z}}_{1}{⊥}_{P}A_{1}|\bar{U}{\bar{B}}_{1}$ , with ${\bar{B}}_{1}=\left\{E,{\overset{ˇ}{Z}}_{1}\right\}$ . Therefore, $I\left({\bar{Z}}_{1};\bar{U}|E,{\overset{ˇ}{Z}}_{1}\right)=I\left({\bar{Z}}_{1};\bar{U},A_{1}|E,{\overset{ˇ}{Z}}_{1}\right)$ . The chain rule of mutual information allows separating in a sum the terms $I({\bar{Z}}_{1};A_{1}|E,{\overset{ˇ}{Z}}_{1})$ and $I({\bar{Z}}_{1};\bar{U}|E,A_{1},{\overset{ˇ}{Z}}_{1})$ . Given that $\Delta P_{1}$ preserves $P({\bar{Z}}_{1},\bar{U},A_{1},E,{\overset{ˇ}{Z}}_{1})$ , these two terms correspond to the terms in the r.h.s. of Equation (23) for k = 1. We now consider the case of k=1 with ${\bar{B}}_{1}^{\prime }\subset \left\{A_{0},{\bar{B}}_{0}\right\}=E$ and the case of any k>1 . We follow three steps. Step k.1 involves intermediate families that allow moving from the preservation of $P({\bar{Z}}_{k-1},{\bar{U}}_{k-1},A_{k-1},{\bar{B}}_{k-1})$ , which enables the application of the DP inequality associated with ${\bar{Z}}_{k-1}{⊥}_{P}A_{k-1}|{\bar{U}}_{k-1}{\bar{B}}_{k-1}$ , to the preservation of $P({\bar{Z}}_{k},{\bar{U}}_{k},A_{k},{\bar{B}}_{k})$ , which allows applying the DP inequality associated with ${\bar{Z}}_{k}{⊥}_{P}A_{k}|{\bar{U}}_{k}{\bar{B}}_{k}$ . Step k.1 proceeds as follows: we compare the family $\Delta P_{k-1}$ that preserves $P({\bar{Z}}_{k-1},{\bar{U}}_{k-1},A_{k-1},{\bar{B}}_{k-1})$ and $P({\bar{Z}}_{j},C_{j})$ , for j=1,…,k−1 , with a family $\Delta P_{k-1}^{\prime }$ defined as preserving $P({\bar{Z}}_{k-1},{\bar{U}}_{k-1},{\bar{B}}_{k})$ , $P({\bar{Z}}_{k-1},C_{k})$ , and $P({\bar{Z}}_{j},C_{j})$ for j=1,…,k−1 . By construction, $C_{k}=\left\{A_{k-1},{\bar{B}}_{k-1},{\overset{ˇ}{Z}}_{k}\right\}$ and ${\overset{ˇ}{Z}}_{k}\subset {\bar{Z}}_{k-1}$ , so $P({\bar{Z}}_{k-1},C_{k})$ is a marginal of $P({\bar{Z}}_{k-1},{\bar{U}}_{k-1},A_{k-1},{\bar{B}}_{k-1})$ . Similarly, by construction ${\bar{B}}_{k}\subseteq C_{k}$ so $P({\bar{Z}}_{k-1},{\bar{U}}_{k-1},{\bar{B}}_{k})$ is also a marginal of $P({\bar{Z}}_{k-1},{\bar{U}}_{k-1},A_{k-1},{\bar{B}}_{k-1})$ . This means that $\Delta P_{k-1}\subseteq \Delta P_{k-1}^{\prime }$ , since the constraints of $\Delta P_{k-1}^{\prime }$ are a subset of the constraints of $\Delta P_{k-1}$ . Accordingly, the term at the l.h.s. of Equation (23) is such that

(A7)
$$\begin{array}{c} \min_{Q\in \Delta P_{k-1}}I_{Q}\left({\bar{Z}}_{k-1};{\bar{U}}_{k-1}|E,A^{\left[k-1\right]},{\overset{ˇ}{Z}}^{\left[k-1\right]}\right)\geq \min_{Q\in \Delta P_{k-1}^{\prime }}I_{Q}\left({\bar{Z}}_{k-1};{\bar{U}}_{k-1}|E,A^{\left[k-1\right]},{\overset{ˇ}{Z}}^{\left[k-1\right]}\right). \end{array}$$

We then further modify the constraints according to

(A8)
$$\begin{array}{cc} \; & \min_{Q\in \Delta P_{k-1}^{\prime }}I_{Q}\left({\bar{Z}}_{k-1};{\bar{U}}_{k-1}|E,A^{\left[k-1\right]},{\overset{ˇ}{Z}}^{\left[k-1\right]}\right)\overset{\left(a\right)}{\geq }\\ & \min_{Q\in \Delta P_{k-1}^{\prime }}I_{Q}\left({\bar{Z}}_{k},{\overset{ˇ}{Z}}_{k};{\bar{U}}_{k}|E,A^{\left[k-1\right]},{\overset{ˇ}{Z}}^{\left[k-1\right]}\right)\overset{\left(b\right)}{\geq }\\ \; & \min_{Q\in \Delta P_{k-1}^{\prime }}I_{Q}\left({\bar{Z}}_{k};{\bar{U}}_{k}|E,A^{\left[k-1\right]},{\overset{ˇ}{Z}}^{\left[k\right]}\right)\overset{\left(c\right)}{\geq }\min_{Q\in \Delta P_{k-1}^{\prime \prime }}I_{Q}\left({\bar{Z}}_{k};{\bar{U}}_{k}|E,A^{\left[k-1\right]},{\overset{ˇ}{Z}}^{\left[k\right]}\right). \end{array}$$

Step (a) holds because $\left\{{\bar{Z}}_{k},{\overset{ˇ}{Z}}_{k}\right\}\subseteq {\bar{Z}}_{k-1}$ and ${\bar{U}}_{k}\subseteq {\bar{U}}_{k-1}$ , so that the information between the subsets can only be smaller than or equal to the information between the sets. In step (b) , the chain rule inequality for minInf mutual information terms (Lemma A1) is used to move ${\overset{ˇ}{Z}}_{k}$ to the conditioning set. In step (c) , the constraints of $\Delta P_{k-1}^{\prime }$ on $P({\bar{Z}}_{k-1},{\bar{U}}_{k-1},{\bar{B}}_{k})$ and $P({\bar{Z}}_{k-1},C_{k})$ are loosed in the marginalized variables of ${\bar{Z}}_{k-1}\backslash \left\{{\bar{Z}}_{k},{\overset{ˇ}{Z}}_{k}\right\}$ and ${\bar{U}}_{k-1}\backslash {\bar{U}}_{k}$ , which now do not appear in the information term. This loosening results in a family $\Delta P_{k-1}^{\prime \prime }$ such that $\Delta P_{k-1}^{\prime }\subseteq \Delta P_{k-1}^{\prime \prime }$ with constraints on $P({\bar{Z}}_{k},{\bar{U}}_{k},{\bar{B}}_{k})$ and $P({\bar{Z}}_{k},C_{k})$ . Note that ${\overset{ˇ}{Z}}_{k}$ is not explicitly written as variables contained in these distributions because ${\overset{ˇ}{Z}}_{k}\subseteq {\bar{B}}_{k}\subseteq C_{k}$ . The latter constraint on $P({\bar{Z}}_{k},C_{k})$ can be grouped with $P({\bar{Z}}_{j},C_{j})$ , for j=1,…,k−1 , resulting in $P({\bar{Z}}_{j},C_{j})$ , for j=1,…,k . We then compare the constraints of $\Delta P_{k-1}^{\prime \prime }$ to the ones of the family $\Delta P_{k}$ . The constraints on $P({\bar{Z}}_{j},C_{j})$ , for j=1,…,k are common. $\Delta P_{k}$ also has a constraint on $P({\bar{Z}}_{k},{\bar{U}}_{k},A_{k},{\bar{B}}_{k})$ , which contains $A_{k}$ , as opposed to the constraint $P({\bar{Z}}_{k},{\bar{U}}_{k},{\bar{B}}_{k})$ of $\Delta P_{k-1}^{\prime \prime }$ . That is, overall step k.1 leads to a lower bound with an information term in which $A_{k}$ can be introduced thanks to the independence ${\bar{Z}}_{k}{⊥}_{P}A_{k}|{\bar{U}}_{k}{\bar{B}}_{k}$ . This insertion of $A_{k}$ is done in step k.2 :

(A9)
$$\begin{array}{c} \min_{Q\in \Delta P_{k-1}^{\prime \prime }}I_{Q}\left({\bar{Z}}_{k};{\bar{U}}_{k}|E,A^{\left[k-1\right]},{\overset{ˇ}{Z}}^{\left[k\right]}\right)=\min_{Q\in \Delta P_{k}}I_{Q}\left({\bar{Z}}_{k};{\bar{U}}_{k},A_{k}|E,A^{\left[k-1\right]},{\overset{ˇ}{Z}}^{\left[k\right]}\right). \end{array}$$

The minInf DP inequality of Proposition 5 is applied with variables $\left\{Z,D,D^{\prime },O_{1},E,E_{2}\right\}$ assigned as $\left\{{\bar{Z}}_{k},{\bar{U}}_{k},A_{k},{\bar{B}}_{k},\left\{E,A^{\left[k-1\right]},{\overset{ˇ}{Z}}^{\left[k\right]}\right\},\emptyset \right\}$ . $\Delta P_{k}$ plays the role of $\Delta P_{DD^{\prime }}$ in Equation (19) and $\Delta P_{k-1}^{\prime \prime }$ the role of $\Delta P_{D}$ . In the final step k.3 , the chain rule inequality for minInf information terms from Lemma A1 is used to separate the observable information term that does not contain ${\bar{U}}_{k}$ :

(A10)
$$\begin{array}{cc} \min_{Q\in \Delta P_{k}}I_{Q}\left({\bar{Z}}_{k};{\bar{U}}_{k},A_{k}|E,A^{\left[k-1\right]},{\overset{ˇ}{Z}}^{\left[k\right]}\right)\geq & \min_{Q\in \Delta P_{k}}I_{Q}\left({\bar{Z}}_{k};A_{k}|E,A^{\left[k-1\right]},{\overset{ˇ}{Z}}^{\left[k\right]}\right)+\\ & \min_{Q\in \Delta P_{k}}I_{Q}\left({\bar{Z}}_{k};{\bar{U}}_{k}|E,A^{\left[k\right]},{\overset{ˇ}{Z}}^{\left[k\right]}\right). \end{array}$$

The first term $\min_{Q\in \Delta P_{k}}I_{Q}\left({\bar{Z}}_{k};A_{k}|E,A^{\left[k-1\right]},{\overset{ˇ}{Z}}^{\left[k\right]}\right)$ only involves observable variables and an estimable information term is obtained relaxing the preservation of $P({\bar{Z}}_{k},{\bar{U}}_{k},A_{k},{\bar{B}}_{k})$ to the preservation of $P({\bar{Z}}_{k},A_{k},{\bar{B}}_{k})$ . The second term $\min_{Q\in \Delta P_{k}}I_{Q}\left({\bar{Z}}_{k};{\bar{U}}_{k}|E,A^{\left[k\right]},{\overset{ˇ}{Z}}^{\left[k\right]}\right)$ has the same form of the term at the l.h.s. of Equation (23), which is $\min_{Q\in \Delta P_{k-1}}I_{Q}\left({\bar{Z}}_{k-1};{\bar{U}}_{k-1}|E,A^{\left[k-1\right]},{\overset{ˇ}{Z}}^{\left[k-1\right]}\right)$ , but with *k* instead of k−1 . This completes iteration *k*. □

Overall, we can summarize the three steps of each iteration *k* in the following way. Step k.1 loosens the constraints on the marginals preserved in the family of distributions. This loosening is the minimum amount required so that the next conditional independence can be used. In fact, the same procedure could be applied with $C_{j}\subseteq \left\{A_{j-1},B_{j-1},{\overset{ˇ}{Z}}_{j}\right\}$ , but that would lead to weaker constraints $P({\bar{Z}}_{j},C_{j})$ for j=1,…,k in the minimization, and hence to an equal or smaller lower bound. Step k.2 corresponds to the application of the DP inequality of Proposition 5. It can be verified that in the scenarios included in Theorem 1, Proposition 5 is always applied with $E_{2}=\emptyset$ . Further generalizations with a nonempty $E_{2}$ are left for future work since they do not qualitatively add to the procedures here developed. Finally, step k.3 applies the chain rule inequality of minInf information terms (Lemma A1) to separate the observable information term.

## Appendix F. Examples of Applications of Theorem 1

In this section, we further examine scenarios comprised in the iterative application of Theorem 1 when used in Proposition 6. We start with the example of Figure A2A, which illustrates that X and the conditioning set $B_{0}$ play the same role in terms of the application of DP inequalities to derive an estimable lower bound. In Figure 2 we have seen examples in which variables belonging to $B_{0}$ are excluded at a certain iteration *k* from the set ${\bar{B}}_{k}$ that in Theorem 1 appears in ${\bar{Z}}_{k}{⊥}_{P}A_{k}|{\bar{U}}_{k}{\bar{B}}_{k}$ . For example, in Figure 2A, with $B_{0}=\left\{W_{1},W_{2}\right\}$ , we have derived the term $I(Z;Y_{2}\backslash \backslash \left\{W_{2},Y_{1}\right\}|W_{1},X)$ relaxing the preservation of $P(Z,U,Y_{1},W_{1},W_{2},X)$ to $P(Z,U,W_{1},X)$ , given that $Z⊥Y_{2}|UXW_{1}$ , while Z⊥Y2|UXW1W2Y1 . The same relaxation is applicable to exclude variables from X , like variables from $B_{0}$ . This is because in Proposition 6 the derivation of the estimable lower bound starts from $I(Z;U|B_{0},X)$ , and hence $\left\{B_{0},X\right\}$ jointly appear as part of all $E_{k}=\left\{B_{0},X,Z^{\left[k-1\right]}\right\}$ . Figure A2A shows an example in which an inequality is derived relaxing the preservation of $P(Z,U,Y_{1},X_{1},X_{2})$ to $P(Z,U,X_{1})$ , hence excluding $X_{2}\in X$ . In more detail, the inequality

(A11)
$$H\left(X_{1},X_{2}|Z\right)\geq I\left(Z;Y_{1}|X_{1},X_{2}\right)+I(Z;Y_{2}\backslash \backslash \left\{X_{2},Y_{1}\right\}\left|X_{1}\right)$$

is derived selecting the instrumental set Z={Z} , the conditioning set $B_{0}=\emptyset$ , and $X=\{X_{1},X_{2}\}$ . The upper bound is obtained with Z⊥U , and the terms in the lower bound are derived with $Z⊥Y_{1}|UX_{1}X_{2}$ and $Z⊥Y_{2}|UX_{1}$ , respectively. To introduce $Y_{2}$ , the minInf constraints are relaxed to exclude $\left\{X_{2},Y_{1}\right\}$ because they are a collider and a descendant of a collider in a path between *Z* and $Y_{2}$ .

Figure A2B shows an example in which the iterative application of DP inequalities requires the marginalization of some hidden variable. The inequality

(A12)
$$H\left(X_{1},X_{2}|W,Z\right)\geq I\left(Z;Y_{1}|X_{1},X_{2},W\right)+I(Z;Y_{2}\backslash \backslash \left\{X_{1},Y_{1},W\right\}\left|X_{2}\right)$$

is derived selecting Z={Z} , $B_{0}=\left\{W\right\}$ , $U=\{U_{1},U_{2}\}$ , and $X=\{X_{1},X_{2}\}$ . The upper bound is derived with $Z⊥\{U_{1},U_{2}\}|W$ . The estimable terms in the lower bound are derived thanks to $Z⊥Y_{1}|U_{1}U_{2}X_{1}X_{2}W$ and $Z⊥Y_{2}|U_{2}X_{2}$ , respectively. In particular, after the application of the first DP inequality the term $I(Z;U_{1},U_{2}|X_{1},X_{2},W,Y_{1})$ is obtained. A second DP inequality cannot be applied directly from this term without first marginalizing $U_{1}$ . This is because $\left\{W,X_{1},Y_{1},U_{1}\right\}$ cannot be in the conditioning set since *W* is a collider in a path between *Z* and $Y_{2}$ , and $\left\{X_{1},Y_{1},U_{1}\right\}$ are its descendants. The hidden variable $U_{1}$ is marginalized given that by monotonicity $I(Z;U_{2}|X_{1},X_{2},W,Y_{1})$ is equal to or smaller than $I(Z;U_{1},U_{2}|X_{1},X_{2},W,Y_{1})$ . The other variables $\left\{W,X_{1},Y_{1}\right\}$ are excluded from the conditioning set relaxing the minInf constraints from $P(Z,U_{2},Y_{1},W,X_{1},X_{2})$ to $P(Z,U_{2},X_{2})$ .

Figure A2C shows an example in which the iterative application of DP inequalities requires the marginalization of some variable from the instrumental set Z . The inequality

(A13)
$$H\left(X_{1},X_{2}|Z_{1},Z_{2}\right)\geq I\left(Z_{1},Z_{2};Y_{1}|X_{1},X_{2}\right)+I(Z_{1};Y_{2}\backslash \backslash \left\{X_{1},Y_{1}\right\}\left|X_{2}\right)$$

is derived selecting $Z=\{Z_{1},Z_{2}\}$ , $B_{0}=\emptyset$ , $U=\{U_{1},U_{2}\}$ , and $X=\{X_{1},X_{2}\}$ . The upper bound follows from $Z_{1}Z_{2}⊥U_{1}U_{2}$ . The terms in the lower bound are derived with DP inequalities associated with $Z_{1}Z_{2}⊥Y_{1}|U_{1}U_{2}X_{1}X_{2}$ and $Z_{1}⊥Y_{2}|U_{1}U_{2}X_{2}$ . After the application of the first DP inequality a term $I(Z_{1},Z_{2};U_{1},U_{2}|X_{1},X_{2},Y_{1})$ is obtained. Since $Z_{2}$ is not separable from $Y_{2}$ by conditioning on any subset of $\left\{U_{1},U_{2},X_{1},X_{2},Y_{1}\right\}$ , the term is marginalized to $I(Z_{1};U_{1},U_{2}|X_{1},X_{2},Y_{1})$ . Subsequently, $\left\{X_{1},Y_{1}\right\}$ are excluded from conditioning by relaxing the minInf constraints, given that they are a collider or descendant of a collider in paths between $Z_{1}$ and $Y_{2}$ .

Finally, Figure A2D shows an example in which the iterative application of DP inequalities requires conditioning on some variable from the instrumental set Z , as opposed to the example of Figure A2C in which variable $Z_{2}\in Z$ was marginalized. The causal structure in Figure A2D is the same as in Figure A2C except that $Z_{2}$ is a noncollider instead of a collider in $Z_{1}-Z_{2}-Y_{2}$ . The inequality

(A14)
$$H\left(X_{1},X_{2}|Z_{1},Z_{2}\right)\geq I\left(Z_{1},Z_{2};Y_{1}|X_{1},X_{2}\right)+I(Z_{1};Y_{2}\backslash \backslash \left\{X_{1},Y_{1}\right\}\left|X_{2},Z_{2}\right)$$

is again derived selecting $Z=\{Z_{1},Z_{2}\}$ , $B_{0}=\emptyset$ , $U=\{U_{1},U_{2}\}$ , and $X=\{X_{1},X_{2}\}$ . The upper bound follows from $Z_{1}Z_{2}⊥U_{1}U_{2}$ and the first DP inequality is applied with $Z_{1}Z_{2}⊥Y_{1}|U_{1}U_{2}X_{1}X_{2}$ . However, now the second DP inequality is associated with $Z_{1}⊥Y_{2}|U_{1}U_{2}X_{2}Z_{2}$ . After the application of the first DP inequality the term $I(Z_{1},Z_{2};U_{1},U_{2}|X_{1},X_{2},Y_{1})$ is obtained. As in Figure A2C, no other DP inequality can be applied to $\left\{Z_{1},Z_{2}\right\}$ , since $Z_{2}$ is adjacent to $Y_{2}$ . Contrarily to Figure A2C, now $Z_{2}$ cannot be marginalized but needs to be moved to conditioning in order to separate $Z_{1}$ and $Y_{2}$ . Accordingly, the chain rule is applied to $I(Z_{1},Z_{2};U_{1},U_{2}|X_{1},X_{2},Y_{1})$ to separate $I(Z_{2};U_{1},U_{2}|X_{1},X_{2},Y_{1})$ and $I(Z_{1};U_{1},U_{2}|X_{1},X_{2},Y_{1},Z_{2})$ . The first term is dropped, and the second term is used to apply the DP inequality associated with $Z_{1}⊥Y_{2}|U_{1}U_{2}X_{2}Z_{2}$ after relaxing the preservation of the marginals to exclude $\left\{X_{1},Y_{1}\right\}$ from the conditioning set.

Overall, in Figure A2 we have provided additional examples that illustrate how instrumental entropic inequalities can be derived from Proposition 6 by relaxing the preservation of marginals that include a subset of X (Figure A2A), by marginalizing on a subset of the hidden variables U (Figure A2B), by marginalizing on a subset of the instrumental set Z (Figure A2C), or by moving part of Z to the conditioning set (Figure A2D). For all these examples, it can be verified that the instrumental entropic inequalities of Equations (A11)–(A14) provide additional causal inference power according to the criterion of Remark A1. For the sake of space, a detailed verification of this criterion is not presented, in particular because our objective here was to further illustrate the versatility within Theorem 1 to build sequences ${\bar{Z}}^{\left[n\right]}$ , ${\bar{B}}^{\left[n\right]}$ , and ${\bar{U}}^{\left[n\right]}$ associated with independencies ${\bar{Z}}_{k}{⊥}_{P}A_{k}|{\bar{U}}_{k}{\bar{B}}_{k}$ for k=1,…,n .

## Appendix G. Estimation of minInf Information Terms

As discussed in Appendix D, the maximum entropy unique information is a special case of a minInf information term in that its estimation constitutes a convex optimization problem. In general, the estimation of minInf information terms requires non-convex optimization techniques. To see this, consider an observable term $\min_{Q\in \Delta P_{k}}I_{Q}\left({\bar{Z}}_{k};A_{k}|E,A^{\left[k-1\right]},{\overset{ˇ}{Z}}^{\left[k\right]}\right)$ as they appear in Theorem 1, where $\Delta P_{k}$ preserves the marginals $P({\bar{Z}}_{k},A_{k},{\bar{B}}_{k})$ and $P({\bar{Z}}_{j},C_{j})$ , for j=1,…,k . We can compare this with the specific case of the unique information as discussed in Appendix D, for which the term to be minimized is $I_{Q}\left(\bar{Z};D_{1}|E\right)$ , with ΔP that preserves $P(\bar{Z},D_{1},O_{1})$ and $P(\bar{Z},E)$ . The key property that renders the estimation of the unique information a convex optimization problem is that preserving $P(\bar{Z},E)$ fixes $H(\bar{Z}|E)$ constant within ΔP , so that minimizing $I_{Q}\left(\bar{Z};D_{1}|E\right)$ corresponds to maximizing $H_{Q}\left(\bar{Z}|E,D_{1}\right)$ , which is concave in ΔP . However, in general, when minimizing $I_{Q}\left({\bar{Z}}_{k};A_{k}|E,A^{\left[k-1\right]},{\overset{ˇ}{Z}}^{\left[k\right]}\right)$ , the constraints of $\Delta P_{k}$ are not such that $H_{Q}\left({\bar{Z}}_{k}|E,A^{\left[k-1\right]},{\overset{ˇ}{Z}}^{\left[k\right]}\right)$ is constant in $\Delta P_{k}$ , since $P({\bar{Z}}_{k},E,A^{\left[k-1\right]},{\overset{ˇ}{Z}}^{\left[k\right]})$ is not one of the marginals comprised in $P({\bar{Z}}_{k},A_{k},{\bar{B}}_{k})$ and $P({\bar{Z}}_{j},C_{j})$ , for j=1,…,k . Therefore, while the constraints on the preservation of the marginals always constitute a set of affine equality constraints, the mutual information term to be minimized is not a convex function of the probability distributions within the family of distributions $\Delta P_{k}$ [65].

Accordingly, in general the estimation of minInf information terms requires non-convex optimization methods [44,71,72]. The form of the mutual information objective function as a difference of entropies suggests that the implementation may benefit from methods developed for successive convex approximation, specifically for differences of convex functions [73]. An alternative approach relies on the use of copula methods to construct minimum information joint distributions [74,75], and further work would need to explore how to expand their use for the set of constraints of the minInf terms of the form of Proposition 5. More generally, the determination of information terms defined in non-convex optimization problems is common in network information theory [35]. Non-convex optimization problems appear in multi-terminal communication channels such as Broadcast channels [76], Gray-Wyner networks [77], or Interference channels [78], as well as in problems of confidential and secure communication (e.g., [79]). We expect that the data processing inequalities we have derived can find applications in other domains of information theory, and benefit from estimation methods developed in those domains. However, a full implementation to estimate minInf information terms is beyond the scope of this work, which focuses on the theoretical derivation of data processing inequalities and entropic inequalities with minInf terms. Nonetheless, to advance in their estimation, we here reexpress the definition of minInf terms of the sort that appear in Theorem 1 with a formulation that separates a convex and a non-convex component of the minimization problem. Based on this formulation, in Appendix H we resume the numerical analysis of examples with Gaussian systems, showing that the addition of other minInf terms together with the unique information can further increase causal inferential power.

**Lemma A2** 
(MinInf information terms as minimal unique information terms). *Consider a mutual information term $I_{Q}\left({\bar{Z}}_{k};A_{k}|E,A^{\left[k-1\right]},{\overset{ˇ}{Z}}^{\left[k\right]}\right)$ defined as in Theorem 1, and to be minimized within the family of distributions $\Delta P_{k}$ that preserves the marginals $P({\bar{Z}}_{k},A_{k},{\bar{B}}_{k})$ and $P({\bar{Z}}_{j},C_{j})$ , for j=1,…,k , with the collections ${\bar{Z}}^{\left[k\right]}$ and $C^{\left[k\right]}$ constructed as in Theorem 1. The minimization problem can be reexpressed as the minimization of a unique information term:*

(A15)
$$\begin{array}{c} \min_{Q\in \Delta P_{k}}I_{Q}\left({\bar{Z}}_{k};A_{k}|E,A^{\left[k-1\right]},{\overset{ˇ}{Z}}^{\left[k\right]}\right)=\min_{\bar{Q}\in \Delta P_{k}}I_{\bar{Q}}({\bar{Z}}_{k};A_{k}\backslash \backslash \left\{\left\{E,A^{\left[k-1\right]},{\overset{ˇ}{Z}}^{\left[k\right]}\right\}\backslash {\bar{B}}_{k}\right\}\left|{\bar{B}}_{k}\right), \end{array}$$

*where $\bar{Q}\left(A_{k},{\bar{Z}}^{\left[k\right]},C^{\left[k\right]}\right)$ has marginals $\bar{Q}\left({\bar{Z}}_{k},A_{k},{\bar{B}}_{k}\right)$ equal to $P({\bar{Z}}_{k},A_{k},{\bar{B}}_{k})$ and $\bar{Q}\left({\bar{Z}}^{\left[k\right]},C^{\left[k\right]}\right)$ that preserves all the marginals $P({\bar{Z}}_{j},C_{j})$ , for j=1,…,k .*

**Proof. ** By construction, $C_{1}=\left\{E,{\overset{ˇ}{Z}}_{1}\right\}$ . Also by construction, $C_{j}=\left\{A_{j-1},{\bar{B}}_{j-1},{\overset{ˇ}{Z}}_{j}\right\}$ . This means that all the variables in the conditioning set $\left\{E,A^{\left[k-1\right]},{\overset{ˇ}{Z}}^{\left[k\right]}\right\}$ appear at least in one of the preserved marginals $P({\bar{Z}}_{j},C_{j})$ , for j=1,…,k . On the other hand, $A_{k}$ only appears in the marginal $P({\bar{Z}}_{k},A_{k},{\bar{B}}_{k})$ . Therefore, the fulfillment of the constraints for a distribution $\bar{Q}\left(A_{k},{\bar{Z}}^{\left[k\right]},C^{\left[k\right]}\right)$ can be separated into $\bar{Q}\left({\bar{Z}}_{k},A_{k},{\bar{B}}_{k}\right)$ being equal to $P({\bar{Z}}_{k},A_{k},{\bar{B}}_{k})$ and the other constraints imposed to the marginal $\bar{Q}\left({\bar{Z}}^{\left[k\right]},C^{\left[k\right]}\right)$ . We can hence separate the minimization within $\Delta P_{k}$ in two steps. First, a minimization that involves the selection of a concrete marginal $\bar{Q}\left({\bar{Z}}^{\left[k\right]},C^{\left[k\right]}\right)$ compatible with $P({\bar{Z}}_{j},C_{j})$ , for j=1,…,k . Subsequently, the minimization operates among the distributions $\bar{Q}\left({\bar{Z}}_{k},A_{k},E,A^{\left[k-1\right]},{\overset{ˇ}{Z}}^{\left[k\right]}\right)$ compatible with the marginals $\bar{Q}\left({\bar{Z}}_{k},A_{k},{\bar{B}}_{k}\right)=P\left({\bar{Z}}_{k},A_{k},{\bar{B}}_{k}\right)$ and the predetermined $\bar{Q}\left({\bar{Z}}_{k},E,A^{\left[k-1\right]},{\overset{ˇ}{Z}}^{\left[k\right]}\right)$ . Note that also by construction, given ${\bar{B}}_{j}^{\prime }\subseteq \left\{A_{j-1},{\bar{B}}_{j-1}\right\}$ and ${\bar{B}}_{j}=\left\{{\bar{B}}_{j}^{\prime },{\overset{ˇ}{Z}}_{j}\right\}$ , then ${\bar{B}}_{k}\subseteq \left\{E,A^{\left[k-1\right]},{\overset{ˇ}{Z}}^{\left[k\right]}\right\}$ . Therefore, the preservation of the two marginals $\bar{Q}\left({\bar{Z}}_{k},A_{k},{\bar{B}}_{k}\right)$ and $\bar{Q}\left({\bar{Z}}_{k},E,A^{\left[k-1\right]},{\overset{ˇ}{Z}}^{\left[k\right]}\right)$ has the form of the constraints that define a unique information. Concretely, following Equation (5), the form of a unique information term is recovered with the assignments of $\bar{Z}$ as ${\bar{Z}}_{k}$ , $D_{1}$ as $A_{k}$ , $D_{2}$ as $\left\{E,A^{\left[k-1\right]},{\overset{ˇ}{Z}}^{\left[k\right]}\right\}\backslash {\bar{B}}_{k}$ , and $O_{1}$ as ${\bar{B}}_{k}$ , such that $P(\bar{Z},D_{1},O_{1})$ of Equation (5) corresponds to $\bar{Q}\left({\bar{Z}}_{k},A_{k},{\bar{B}}_{k}\right)$ and $P(\bar{Z},D_{2},O_{1})$ corresponds to $\bar{Q}\left({\bar{Z}}_{k},E,A^{\left[k-1\right]},{\overset{ˇ}{Z}}^{\left[k\right]}\right)$ . □

## Appendix H. Numerical Examples with Additional minInf Information Terms

We now resume the analysis of Appendix D to numerically study the entropic inequality of Equation (25), associated with Figure 2C. We use Lemma A2 to rewrite Equation (25) and we also express the unique information terms specifically for Gaussian variables:

(A16)
$$\begin{array}{cc} \; & H\left(X|Z,W_{2},W_{3}\right)\geq I\left(Z;Y_{1}|W_{2},W_{3},X\right)+\min_{Q\in \Delta P_{2}}I_{Q}\left(Z;Y_{2}|W_{2},W_{3},X,Y_{1}\right)+\\ & \min_{Q\in \Delta P_{3}}I_{Q}\left(Z;Y_{3}|W_{2},W_{3},X,Y_{1},Y_{2}\right)\overset{\left(a\right)}{=}I\left(Z;Y_{1}|W_{2},W_{3},X\right)+I(Z;Y_{2}\backslash \backslash \left\{W_{2},Y_{1}\right\}\left|W_{3},X\right)+\\ & \min_{\bar{Q}\in \Delta P_{3}}I_{\bar{Q}}(Z;Y_{3}\backslash \backslash \left\{W_{2},W_{3},Y_{1}\right\}\left|X,Y_{2}\right)\overset{\left(b\right)}{=}I\left(Z;Y_{1}|W_{2},W_{3},X\right)+\max\left\{I(Z;Y_{2}|W_{3},X)-\right.\\ & I\left(Z;W_{2},Y_{1}|W_{3},X\right)\left.,0\right\}+\min_{\bar{Q}\in \Delta P_{3}}\max\left\{I_{\bar{Q}}\left(Z;Y_{3}|X,Y_{2}\right)-I_{\bar{Q}}\left(Z;W_{2},W_{3},Y_{1}|X,Y_{2}\right),0\right\}, \end{array}$$

where $\Delta P_{2}$ preserves the marginals $\left\{P\left(Z,W_{3},X,Y_{2}\right),P\left(Z,W_{2},W_{3},X,Y_{1}\right)\right\}$ and $\Delta P_{3}$ preserves the marginals $\left\{P\left(Z,X,Y_{2},Y_{3}\right),P\left(Z,W_{3},X,Y_{2}\right),P\left(Z,W_{2},W_{3},X,Y_{1}\right)\right\}$ . Equality (a) uses Lemma A2 to reexpress the second minInf term as the minimization of a unique information estimated on distributions $\bar{Q}\in \Delta P_{3}$ that factorize as $\bar{Q}=P\left(Z,X,Y_{2},Y_{3}\right)Q\left(W_{2},W_{3},Y_{1}|Z,X,Y_{2},Y_{3}\right)$ and preserve the marginals $P(Z,W_{3},X,Y_{2})$ and $P(Z,W_{2},W_{3},X,Y_{1})$ . Equality (b) expresses the unique information terms specifically for Gaussian variables, given their form in Equation (A3).

We proceed analogously to Appendix D, simulating variables generated with a system that conforms to the causal structure of Figure 2C, but with additional direct connections $Z\to Y_{1}$ , $Z\to Y_{2}$ , and $Z\to Y_{3}$ . We also keep the connection $X↔W_{2}$ so that other than for the inclusion of the new observable variables $W_{3}$ and $Y_{3}$ , the generative process of the observable variables is the same as in Appendix D. If not for the connections $Z\to Y_{1}$ , $Z\to Y_{2}$ , and $Z\to Y_{3}$ , the system would causally fulfill the inequality of Equation (25), but these connections can lead to violations. We again generate the variables as a system of linear equations with Gaussian noise. Following the same strategy of Appendix D, we keep fixed the values of most parameters and examine concrete settings in which the strength of the connection $Z\to Y_{3}$ changes, as determined by the coefficient $a_{y_{3}z}$ . In more detail, we again select a default configuration in which the standard deviation of all hidden confounders is set to 1, and their mean set to zero. Again all nodes apart from *Z* are generated having also some exogenous independent noises, all of which are generated with mean zero and standard deviation *v*. All coefficients associated with the connections are again by default set to 1, if not indicated otherwise. For the connections exclusive of Figure 2C in comparison to Figure 2A, we set $a_{w_{3}z}=a_{w_{3}u^{\prime \prime }}=a_{y_{3}u^{\prime \prime }}=2$ , where $U^{\prime \prime }$ is the hidden confounder in $W_{3}↔Y_{3}$ .

Since in this Appendix we are interested in examining the additional causal inference power gained with the extra minInf term associated with $Y_{3}$ in Equation (A16), we focus on configurations for which the strength of both $Z\to Y_{1}$ and $Z\to Y_{2}$ is not enough to violate the inequality. We fixed $a_{y_{1}z}=a_{y_{2}z}=1$ and verified that a violation does not occur due to these connections. Accordingly, we here examine configurations in which in Equation (A16) the sum of $I(Z;Y_{1}|W_{2},W_{3},X)$ and $I(Z;Y_{2}\backslash \backslash \left\{W_{2},Y_{1}\right\}|W_{3},X)$ at the r.h.s. is smaller than the upper bound $H(X|Z,W_{2},W_{3})$ , so that it is the additional minInf term associated with $Y_{3}$ the one that determines whether a violation occurs. Apart from exploring different strengths of $a_{y_{3}z}$ , we extend the analysis of Figure A1G–I, and examine configurations with different values of the standard deviation *v* of the exogenous noises.

To find the minimum of $\bar{Q}\in \Delta P_{3}$ we proceed by exploring the space of joint distributions compatible with the preservation of the marginals $\{P\left(Z,X,Y_{2},Y_{3}\right),P\left(Z,W_{3},X,Y_{2}\right),$ $P(Z,W_{2},W_{3},X,Y_{1})\}$ . This preservation is reflected in fixed entries of the covariance matrix, which for Gaussian variables determines all information terms. Given the symmetry of the covariance matrix, the preservation of these marginals results in 5 remaining degrees of freedom to explore joint distributions within $\Delta P_{3}$ . We sampled joint distributions with valid covariance matrices covering the range of the non-fixed entries of the matrix with a grid of 50 samples along each of the degrees of freedom, hence probing in the order of $3\times 10^{8}$ joint distributions. Figure A3 shows the results of testing the inequality of Equation (25) across configurations with varying $a_{y_{3}z}$ and *v*. As in Figure A1, we display the test statistic corresponding to the upper bound minus the lower bound of the inequality, such that a violation occurs for negative values.

Figure A3 shows that violations of the inequality occur with an increasing strength of $Z\to Y_{3}$ . The terms $I(Z;Y_{1}|W_{2},W_{3},X)$ and $I(Z;Y_{2}\backslash \backslash \left\{W_{2},Y_{1}\right\}|W_{3},X)$ , as well as the upper bound $H(X|Z,W_{2},W_{3})$ , are constant to changes in $a_{y_{3}z}$ , and hence the decrease of the statistic and the occurrence of negative values is due to $\min_{Q\in \Delta P_{3}}I_{Q}\left(Z;Y_{3}|W_{2},W_{3},X,Y_{1},Y_{2}\right)$ . This illustrates that the addition of new minInf terms in the instrumental entropic inequality provides additional causal inference power.

Nonetheless, the lesser smoothness of the curves in Figure A3 compared to Figure A1 reflects the difficulty to estimate minInf terms in general. In Lemma A2, we have shown how to separate convex and non-convex parts of this estimation, and here we have restricted the simulations to multivariate Gaussian variables in order to benefit from the known form of unique information in these systems [68]. In our simulations the family of distributions to be explored is characterized by five degrees of freedom of the covariance matrix, making an exhaustive exploration of the family manageable, although computationally expensive. More broadly, non-convex optimization methods need to be adopted for the estimation of minInf terms, as described in Appendix G. The main contribution of this work has been the theoretical development of how minInf terms can be used to increase the causal inference power of entropic inequalities. The derived minInf data processing inequalities open a line of research to extend further types of entropic inequalities beyond the ones here considered. In order to make these new inequalities applicable in general, future work will also need to develop optimization methods that allow a reliable estimation of minInf terms.

Apart from the specific challenge of estimating minInf information terms due to the non-convexity of the minimization problem, the estimation from finite data sets is expected to involve additional difficulties ubiquitous for information-theoretic measures. While bias-correction methods have been widely studied for the standard measures [80,81], only recently bias-correction methods have been studied for the maximum entropy unique information [56]. To apply entropic inequalities with minInf terms to systems with many variables or variables with large cardinality, the analysis of bias corrections will need to be extended. Nonetheless, even for complex graphs that include many nodes, causally informative entropic inequalities may imply only subsets of nodes and therefore still be implementable.

## Appendix I. Complementary Instrumental Entropic Inequalities in Figure 2C

We here extend the characterization of causally-fulfilled instrumental entropic inequalities in the causal structure of Figure 2C. In Section 3.5, we examined the inequality of Equation (25) as an example of application of Proposition 6 for which three DP inequalities are iteratively applied, starting from $I(Z;U|X,W_{2},W_{3})$ , with Z={Z} and $B_{0}=\left\{W_{2},W_{3}\right\}$ . We here instead examine instrumental entropic inequalities derived with $Z=\{Z,W_{2},W_{3}\}$ and $B_{0}=\emptyset$ .

In general, to characterize all existing instrumental entropic inequalities, we would need to apply Proposition 6 with all possible partitions $Z^{\left[r\right]}$ for Proposition 3. However, our objective here is not to fully characterize the concrete case of Figure 2C, but to exemplify in more detail how to derive complementary instrumental inequalities with Proposition 6. For this reason, we focus on partitions $Z^{\left[r\right]}$ that use the chain rule to decompose $Z=\{Z,W_{2},W_{3}\}$ separating individual variables sequentially. This results in six possible partitions, as shown in the second column of Table A1. Columns three to five show the three nonestimable information terms resulting from each partition. For each of them, we indicate associated independencies that allow applying DP inequalities to derive estimable lower bounds. The resulting instrumental entropic inequalities are given below, with the indexes of the inequalities mapping the indexes of rows in Table A1:

**Table A1:** Properties associated with the instrumental entropic inequalities presented in Appendix I, which are causally fulfilled by the causal structure of Figure 2C. The label of each row maps to subequations in Equation (A17). The second column indicates the order in which the chain rule is applied in Proposition 3, starting from $Z=\{Z,W_{2},W_{3}\}$ and $B_{0}=\emptyset$ . Columns three to five provide the information terms in the chain decomposition and associated independencies that allow adding estimable information terms by applying Proposition 6.

(a)	$\left\{\left\{Z\right\},\left\{W_{2}\right\},\left\{W_{3}\right\}\right\}$	I(Z;U|X)	$I(W_{2};U|X,Z)$	$I(W_{3};U|X,Z,W_{2})$
		$Z⊥\{Y_{2},Y_{3}\}|UX$	∅	∅
(b)	$\left\{\left\{Z\right\},\left\{W_{3}\right\},\left\{W_{2}\right\}\right\}$	I(Z;U|X)	$I(W_{3};U|X,Z)$	$I(W_{2};U|X,Z,W_{3})$
		$Z⊥\{Y_{2},Y_{3}\}|UX$	∅	∅
(c)	$\left\{\left\{W_{2}\right\},\left\{Z\right\},\left\{W_{3}\right\}\right\}$	$I(W_{2};U|X)$	$I(Z;U|X,W_{2})$	$I(W_{3};U|X,Z,W_{2})$
		$W_{2}⊥Y_{3}|UX$	$Z⊥\left\{Y_{1}Y_{3}\right\}|UXW_{2}$	∅
			$Z⊥Y_{2}|UXY_{3}$	
(d)	$\left\{\left\{W_{3}\right\},\left\{Z\right\},\left\{W_{2}\right\}\right\}$	$I(W_{3};U|X)$	$I(Z;U|X,W_{3})$	$I(W_{2};U|X,Z,W_{3})$
		$W_{3}⊥Y_{2}|UX$	$Z⊥Y_{2}|UXW_{3}$	∅
			$Z⊥Y_{3}|UXY_{2}$	
(e)	$\left\{\left\{W_{2}\right\},\left\{W_{3}\right\},\left\{Z\right\}\right\}$	$I(W_{2};U|X)$	$I(W_{3};U|X,W_{2})$	$I(Z;U|X,W_{2},W_{3})$
		$W_{2}⊥Y_{3}|UX$	$W_{3}⊥Y_{1}|UXW_{2}$	$Z⊥Y_{1}|UXW_{2}W_{3}$
			$W_{3}⊥Y_{2}|UX$	$Z⊥Y_{2}|UXW_{3}$
				$Z⊥Y_{3}|UXY_{2}$
(f)	$\left\{\left\{W_{3}\right\},\left\{W_{2}\right\},\left\{Z\right\}\right\}$	$I(W_{3};U|X)$	$I(W_{2};U|X,W_{3})$	$I(Z;U|X,W_{2},W_{3})$
		$W_{3}⊥Y_{2}|UX$	$W_{2}⊥Y_{3}|UX$	$Z⊥Y_{1}|UXW_{2}W_{3}$
				$Z⊥Y_{3}|UXW_{2}Y_{1}$
				$Z⊥Y_{2}|UXY_{3}$

$$\begin{array}{c} H(X|Z,W_{2},W_{3})\geq \end{array}$$

(A17a)
$$\begin{array}{c} \;\;\geq I(Z;Y_{2},Y_{3}|X) \end{array}$$

(A17b)
$$\begin{array}{c} \;\;\geq I(Z;Y_{2},Y_{3}|X) \end{array}$$

(A17c)
$$\begin{array}{c} \;\;\geq I\left(W_{2};Y_{3}|X\right)+I\left(Z;Y_{1}Y_{3}|X,W_{2}\right)+I(Z;Y_{2}\backslash \backslash \left\{W_{2},Y_{1}\right\}\left|X,Y_{3}\right) \end{array}$$

(A17d)
$$\begin{array}{c} \;\;\;\;\geq I\left(W_{3};Y_{2}|X\right)+I\left(Z;Y_{2}|X,W_{3}\right)+I(Z;Y_{3}\backslash \backslash W_{3}\left|X,Y_{2}\right) \end{array}$$

(A17e)
$$\begin{array}{c} \;\;\geq I\left(W_{2};Y_{3}|X\right)+I\left(W_{3};Y_{1}|X,W_{2}\right)+I(W_{3};Y_{2}\backslash \backslash \left\{W_{2},Y_{1}\right\}\left|X\right)+\\ \;\;\;\;I\left(Z;Y_{1}|X,W_{2},W_{3}\right)+I(Z;Y_{2}\backslash \backslash \left\{W_{2},Y_{1}\right\}\left|X,W_{3}\right)+ \end{array}$$

(A17f)
$$\begin{array}{c} \min_{Q\in \Delta P_{3}}I_{Q}\left(Z;Y_{3}|X,W_{2},W_{3},Y_{1},Y_{2}\right)\\ \;\;\geq I\left(W_{3};Y_{2}|X\right)+I(W_{2};Y_{3}\backslash \backslash W_{3}\left|X\right)+I\left(Z;Y_{1}|X,W_{2},W_{3}\right)+\\ \;\;\;\;\;\;\;I(Z;Y_{3}\backslash \backslash W_{3}\left|X,W_{2},Y_{1}\right)+\min_{Q\in \Delta P_{3}^{\prime }}I_{Q}\left(Z;Y_{2}|X,W_{2},W_{3},Y_{1},Y_{3}\right), \end{array}$$

where $\Delta P_{3}$ preserves $P(Z,X,W_{2},W_{3},Y_{1})$ , $P(Z,X,W_{3},Y_{2})$ , and $P(Z,X,Y_{2},Y_{3})$ , while $\Delta P_{3}^{\prime }$ preserves $P(Z,X,W_{2},W_{3},Y_{1})$ , $P(Z,X,W_{2},Y_{1},Y_{3})$ , and $P(Z,X,Y_{2},Y_{3})$ . Note that the upper bound is common to all inequalities. Each subequation should be read as comparing the l.h.s. with each individual r.h.s. with no order between the r.h.s. of the different subequations. Partitions (a) and (b) result in the same instrumental entropic inequality. It can be verified that the resulting five different inequalities of Equation (A17) provide complementary causal inference power. The inequality of Equation (A17e) subsumes the inequality of Equation (25). This is seen straightforwardly moving $I(Z,X|W_{2},W_{3})$ from the r.h.s. to the l.h.s. of Equation (25). Altogether, this further analysis illustrates that Proposition 6 allows deriving sets of instrumental entropic inequalities that exploit different combinations of independencies present in the causal structure, hence providing additional causal inference power to the standard instrumental entropic inequalities.

## Appendix J. A Hierarchy Between Instrumental Entropic Inequalities Using Multivariate Instrumental Sets

We here consider the relation between instrumental entropic inequalities constructed with a multivariate instrumental set Z and instrumental entropic inequalities constructed by using as instruments only subsets of Z . We focus on a more restricted scenario than the one of Remark A1. Instead of considering all causal structures $G(I_{V})$ compatible with an available set of testable conditional independencies $I_{V}$ , we consider a scenario in which, using a concrete set of hidden independencies, two specific types of inequalities are to be compared in their causal inference power to discard a single causal structure of interest:

**Proposition A1** 
(A hierarchy of instrumental entropic inequalities). *Consider nonoverlapping sets of variables Z , $B_{0}$ , X , and U , all observable except U hidden variables. Consider that the causal structure of interest whose compatibility with data is to be tested is such that it creates an independence $Z⊥U|B_{0}$ , so that Z is a multivariate instrumental set. Consider a nonoverlapping partition $Z=\{Z_{1},Z_{2},Z_{3},Z_{4}\}$ , with $Z_{1}$ nonempty, and $Z_{2}$ , $Z_{3}$ , and $Z_{4}$ possibly empty. Consider that the causal structure also creates a set of m independencies that allow the use of *DP* inequalities by recursively applying Theorem 1 with initial inputs $\bar{Z}=Z_{1}$ and $E=\{B_{0},X,Z_{2}\}$ , resulting in the introduction of estimable information terms with observable variables $A^{\left[m\right]}$ . In this case, an instrumental entropic inequality derived applying Theorem 1 with instruments $Z_{1}$ , and departing from $I(Z_{1};U,X|B_{0},Z_{2},Z_{3})$ , does not add causal inference power to the instrumental inequality derived applying Theorem 1 with the whole instrumental set Z and departing from $I(Z;U,X|B_{0})$ .*

**Proof. ** Given that the causal structure of interest fulfills $Z⊥U|B_{0}$ , the *weak union* axiom of semi-graphoids [25,43] guarantees that it also fulfills $Z_{1}⊥U|B_{0}Z_{2}Z_{3}$ . The instrumental inequality developed using $Z⊥U|B_{0}$ has as upper bound the entropy $H(X|Z,B_{0})$ , while the inequality developed using $Z_{1}⊥U|B_{0}Z_{2}Z_{3}$ , with $B_{0}^{\prime }=\left\{B_{0},Z_{2},Z_{3}\right\}$ , has as upper bound the entropy $H(X|Z\backslash Z_{4},B_{0})$ . The observable lower bounds are obtained applying the DP inequalities departing from $I(Z;U|B_{0},X)$ and from $I(Z_{1};U|B_{0},X,Z_{2},Z_{3})$ , respectively. Since the DP inequalities rely on independencies that are applied following Theorem 1 with $\bar{Z}=Z_{1}$ and $E=\{B_{0},X,Z_{2}\}$ , this means that no variable from $\left\{Z_{3},Z_{4}\right\}$ appears in the conditioning set of those independencies. Therefore, before the iterative insertion of observable terms, $I(Z;U|B_{0},X)$ is marginalized to $I(Z_{1},Z_{2};U|B_{0},X)$ . Furthermore, since $\bar{Z}=Z_{1}$ , this means that $Z_{2}$ only appears in the conditioning sets of the independencies used in the DP inequalities, and hence the departing term is further reduced to $I(Z_{1};U|B_{0},X,Z_{2})$ . We now compare this departing term $I(Z_{1};U|B_{0},X,Z_{2})$ used when Z is the instrumental set, and the departing term $I(Z_{1};U|B_{0},X,Z_{2},Z_{3})$ , used when $Z_{1}$ is the instrumental set. If $Z_{3}$ is empty, then the lower bound obtained from $I(Z_{1};U|B_{0},X,Z_{2},Z_{3})$ is the same as the one obtained from $I(Z_{1};U|B_{0},X,Z_{2})$ . In this case, since the upper bound $H(X|Z\backslash Z_{4},B_{0})$ is equal to or higher than $H(X|Z,B_{0})$ , the entropic inequality derived with instrumental set $Z_{1}$ does not add causal inference power. If $Z_{3}\neq \emptyset$ , then in $I(Z_{1};U|B_{0},X,Z_{2},Z_{3})$ the variables $Z_{3}$ are part of the conditioning set but do not appear in any of the conditioning sets of the conditional independencies associated with the DP inequalities. This means that to apply the first DP inequality in the first iteration of Theorem 1 the marginals preserved are relaxed, separating $Z_{3}$ in order to obtain the marginal $P({\bar{Z}}_{1},{\bar{U}}_{1},A_{1},{\bar{B}}_{1})$ , with ${\bar{Z}}_{1}\subseteq Z_{1}$ . That is, the marginal $P({\bar{Z}}_{1},C_{1})$ preserved jointly with $P({\bar{Z}}_{1},{\bar{U}}_{1},A_{1},{\bar{B}}_{1})$ if starting from $I(Z_{1};U|B_{0},X,Z_{2})$ , is replaced by $P({\bar{Z}}_{1},C_{1}^{\prime })$ with $C_{1}^{\prime }=\left\{C_{1},Z_{3}\right\}$ when starting from $I(Z_{1};U|B_{0},X,Z_{2},Z_{3})$ . After the first iteration the series of preserved marginals $P({\bar{Z}}_{j},C_{j})$ is the same as $P({\bar{Z}}_{j},C_{j}^{\prime })$ , that is, $C_{j}^{\prime }=C_{j}$ for j>1 , since they are determined from $P({\bar{Z}}_{1},{\bar{U}}_{1},A_{1},{\bar{B}}_{1})$ recursively constructing them as $C_{j}=\left\{A_{j-1},{\bar{B}}_{j-1},{\overset{ˇ}{Z}}_{j}\right\}$ . Lemma A3 (see below) guarantees that each resulting observable term added to the lower bound with the application of each DP inequality will be equal or smaller starting from $I(Z_{1};U|B_{0},X,Z_{2},Z_{3})$ than starting from $I(Z_{1};U|B_{0},X,Z_{2})$ . Assembling the upper and lower bounds, the upper bound obtained using the whole instrumental set Z is smaller than or equal to the one obtained using the subset $Z_{1}$ . On the other hand, each term in the lower bound is higher or equal with Z , which means that the lower bound is higher or equal. Altogether, the instrumental inequality constructed with Z will always be violated when the one constructed with $Z_{1}$ is violated. Therefore, the test with $Z_{1}$ does not provide additional causal inference power. □

Note that Proposition A1 compares the power of two specific types of tests derived with an instrumental set Z or its subsets. Concretely, the comparison regards tests derived using the same set of hidden independencies to apply DP inequalities. On the other hand, this hierarchy does not preclude from other tests with an instrumental set $Z_{1}\subset Z$ to add causal inference power. If with instrumental set Z in iteration *j* a certain independence ${\bar{Z}}_{j}⊥A_{j}|{\bar{U}}_{j}{\bar{B}}_{j}$ , with ${\bar{Z}}_{j}\subseteq Z_{1}$ is exploited, alternatively, using as instrumental set $Z_{1}$ another independence ${\bar{Z}}_{j}^{\prime }⊥A_{j}^{\prime }|{\bar{U}}_{j}^{\prime }{\bar{B}}_{j}^{\prime }Z_{3j}$ could be exploited, with ${\bar{Z}}_{j}^{\prime }\subseteq Z_{1}$ and $Z_{3j}\subseteq Z_{3}$ . Contrarily to the case in which the same independencies are applied, now the inserted estimable information terms added in the lower bound are not comparable with Lemma A3.

We now present Lemma A3, which is used in the proof above. For the sake of space, instead of stating the property in Lemma A3 in general terms and then showing its application in Proposition A1, we directly present it as applied in the proof.

**Lemma A3** 
(Decrease of information through conditioning for minInf mutual information terms preserving only marginals). *Consider nonoverlapping sets of variables Z , $B_{0}$ , X , and U , all observable except U hidden variables. Consider that $Z⊥U|B_{0}$ . Consider a nonoverlapping partition $Z=\{Z_{1},Z_{2},Z_{3},Z_{4}\}$ , with $Z_{1}$ nonempty, and $Z_{2}$ , $Z_{3}$ , and $Z_{4}$ possibly empty. Consider a set of independencies that allows constructing instrumental entropic inequalities by recursively applying Theorem 1 with initial inputs $\bar{Z}=Z_{1}$ and $E=\{B_{0},X,Z_{2}\}$ . Consider a derived estimable term $\min_{Q\in \Delta P_{k}}I_{Q}\left({\bar{Z}}_{k};A_{k}|E,A^{\left[k-1\right]},{\overset{ˇ}{Z}}^{\left[k\right]}\right)$ , where $\Delta P_{k}$ preserves $P({\bar{Z}}_{k},A_{k},{\bar{B}}_{k})$ and $P({\bar{Z}}_{j},C_{j})$ , for j=1,…,k . Consider another estimable term derived with Theorem 1 using the same set of independencies but starting with $\bar{Z}=Z_{1}$ and $E^{\prime }=\left\{B_{0},X,Z_{2},Z_{3}\right\}$ , with the form $\min_{Q\in \Delta P_{k}^{\prime }}I_{Q}\left({\bar{Z}}_{k};A_{k}|E^{\prime },A^{\left[k-1\right]},{\overset{ˇ}{Z}}^{\left[k\right]}\right)$ , where $\Delta P_{k}^{\prime }$ preserves $P({\bar{Z}}_{k},A_{k},{\bar{B}}_{k})$ and $P({\bar{Z}}_{j},C_{j}^{\prime })$ , with $C_{j}^{\prime }=C_{j}$ for j=2,…,k and $C_{1}^{\prime }=\left\{C_{1},Z_{3}\right\}$ . The minInf terms are such that*

(A18)
$$\begin{array}{c} \min_{Q\in \Delta P_{k}}I_{Q}\left({\bar{Z}}_{k};A_{k}|E,A^{\left[k-1\right]},{\overset{ˇ}{Z}}^{\left[k\right]}\right)\geq \min_{Q\in \Delta P_{k}^{\prime }}I_{Q}\left({\bar{Z}}_{k};A_{k}|E^{\prime },A^{\left[k-1\right]},{\overset{ˇ}{Z}}^{\left[k\right]}\right). \end{array}$$

**Proof. ** Consider a distribution that minimizes the l.h.s. of Equation (A18)

(A19)
$$\begin{array}{c} Q^{*}\left({\bar{Z}}_{k},A_{k},E,A^{\left[k-1\right]},{\overset{ˇ}{Z}}^{\left[k\right]}\right)\equiv \arg\min_{Q\in \Delta P_{k}}I_{Q}\left({\bar{Z}}_{k};A_{k}|E,A^{\left[k-1\right]},{\overset{ˇ}{Z}}^{\left[k\right]}\right). \end{array}$$

For this distribution, the information $I_{Q^{*}}\left({\bar{Z}}_{k};A_{k}|E,A^{\left[k-1\right]},{\overset{ˇ}{Z}}^{\left[k\right]}\right)$ does not depend on $E^{\prime }\backslash E=Z_{3}$ . A joint distribution with $Z_{3}$ can be created as $\bar{Q}\equiv P\left(Z_{3}|{\bar{Z}}_{1},C_{1}\right)Q^{*}\left({\bar{Z}}_{k},A_{k},E,A^{\left[k-1\right]},{\overset{ˇ}{Z}}^{\left[k\right]}\right)$ , with ${\bar{Z}}_{1}$ and $C_{1}$ identified by the constraint of $\Delta P_{k}$ on the marginal $P({\bar{Z}}_{1},C_{1})$ . The decomposition of information into unique information and redundancy [29] can then be applied to $\bar{Q}$ , obtaining the conditional unique information (Equation (5))

(A20)
$$\begin{array}{c} I_{\bar{Q}}({\bar{Z}}_{k};A_{k}\backslash \backslash Z_{3}\left|E,A^{\left[k-1\right]},{\overset{ˇ}{Z}}^{\left[k\right]}\right)=\min_{Q\in \Delta \bar{Q}}I_{Q}\left({\bar{Z}}_{k};A_{k}|E^{\prime },A^{\left[k-1\right]},{\overset{ˇ}{Z}}^{\left[k\right]}\right), \end{array}$$

where by construction $E^{\prime }=\left\{E,Z_{3}\right\}$ , and $\Delta \bar{Q}$ is the family of distributions that preserve $\bar{Q}\left({\bar{Z}}_{k},A_{k},E,A^{\left[k-1\right]},{\overset{ˇ}{Z}}^{\left[k\right]}\right)$ and $\bar{Q}\left({\bar{Z}}_{k},E^{\prime },A^{\left[k-1\right]},{\overset{ˇ}{Z}}^{\left[k\right]}\right)$ . This unique information is by construction (Equation (6)) smaller than or equal to the l.h.s. of Equation (A18). We now compare it with the term in the r.h.s. of Equation (A18). The conditional information minimized is the same. $\Delta \bar{Q}$ preserves $\bar{Q}\left({\bar{Z}}_{k},A_{k},E,A^{\left[k-1\right]},{\overset{ˇ}{Z}}^{\left[k\right]}\right)$ , which by construction is $Q^{*}\left({\bar{Z}}_{k},A_{k},E,A^{\left[k-1\right]},{\overset{ˇ}{Z}}^{\left[k\right]}\right)$ . Since $Q^{*}$ belongs to the family $\Delta P_{k}$ , this constraint implies preserving $P({\bar{Z}}_{k},A_{k},{\bar{B}}_{k})$ and $P({\bar{Z}}_{j},C_{j})$ , for j=1,…,k . Furthermore, $\Delta \bar{Q}$ preserves $\bar{Q}\left({\bar{Z}}_{k},E^{\prime },A^{\left[k-1\right]},{\overset{ˇ}{Z}}^{\left[k\right]}\right)$ , which given the construction of $\bar{Q}$ as $\bar{Q}=P\left(Z_{3}|{\bar{Z}}_{1},C_{1}\right)Q^{*}$ implies the preservation of the marginal $P({\bar{Z}}_{1},C_{1},Z_{3})$ = $P({\bar{Z}}_{1},C_{1}^{\prime })$ . Accordingly, the constraints of $\Delta P_{k}^{\prime }$ are superseeded by the constraints of $\Delta \bar{Q}$ and hence the information term can be further minimized within $\Delta P_{k}^{\prime }$ in the r.h.s. of Equation (A18). □
